# Multi-locus sequence typing (MLST) of non-fermentative Gram-negative bacilli isolated from bloodstream infections in southern Poland

**DOI:** 10.1007/s12223-017-0550-7

**Published:** 2017-09-22

**Authors:** Agnieszka Chmielarczyk, Monika Pobiega, Dorota Romaniszyn, Jadwiga Wójkowska-Mach

**Affiliations:** Department of Microbiology, Jagiellonian University Medical College, 18 Czysta Street, 31-121 Krakow, Poland

**Keywords:** Non-fermentative gram negative bacilli, Bloodstream infection, MLST

## Abstract

Non-fermentative Gram-negative bacilli are now one of the most important causes of severe infections in Polish hospitals. Acinetobacter species are serious concern because of the high prevalence of multi-drug resistance among strains. Resistance profiles for 53 Gram-negative non-fermentative blood isolates were done. MLST was carried out using 44 strains representing the most commonly isolated species: A. baumannii, *P. aeruginosa*, and S. maltophilia. MLST revealed that all 22 A. baumannii belonged to sequence type (ST) 2. The *P. aeruginosa* isolates belonged to 10 different STs. Four S. maltophilia isolates matched STs present in the database (ST4, ST15, ST116, ST142), seven isolates showing novel sequence types. Among *P. aeruginosa* and S. maltophilia PFGE confirmed the genetical variety of strains.

**Electronic supplementary material:**

The online version of this article (10.1007/s12223-017-0550-7) contains supplementary material, which is available to authorized users.

## Introduction

Infections, including bloodstream infections (BSI), caused by non-fermentative Gram-negative bacteria are an emerging problem in hospital settings, especially when caused by multi-drug resistant (MDR) strains. The most commonly isolated bacterial species are *Acinetobacter baumannii* and *Pseudomonas aeruginosa*, with infections caused by these species significantly outnumbering those caused by other species such as *Stenotrophomonas maltophilia*. Gram-negative bacteria are ubiquitous in the environment, but MDR *A.baumannii* adapts extremely quickly to the hospital environment, and may cause outbreaks (Wybo et al. 2007; Baang et al. 2012).

According to World Health Organization data, the number of BSI caused by *P. aeruginosa* is increasing: from 2009 to 2010 about 3.8% of BSI were caused by *P. aeruginosa*, compared with about 3% from 1992 to 1997. Interestingly, 17% of pneumonia cases were caused by *P. aeruginosa* in 2010, which was lower than in 1997 (21%) (Sievert et al. 2013). In addition, the mortality rate for BSI caused by *P. aeruginosa* is quite high (about 30%), with *P. aeruginosa* being identified as a risk factor for mortality in BSI cases (Pena et al. 2012a, b). In China, the mortality rate is even higher, with about 40% of BSI caused by *P. aeruginosa* resulting in death (Kang et al. 2003).

In a national survey of hospital laboratories, *A. baumannii* infections accounted for only 1.3% of healthcare-associated BSI (Wisplinghoff et al. 2004). However, other studies have indicated an increase in the number of reported BSI caused by *A. baumannii*, such as one study in patients from military medical facilities in Iraq, Kuwait, and Afghanistan (Centers for Disease Control and Prevention 2004). S. *maltophilia* is naturally resistant to many antimicrobial agents, including carbapenems, what may be the cause of treatment failure in infections linked to this bacterium. Therefore, the overall mortality rate of *S. maltophilia* BSI is very high, and ranges from 21% to 50% (Araoka et al. 2010; Senol et al. 2002; Garazi et al. 2012). One study found that risk factors associated with *S. maltophilia* bacteremia included use of carbapenems and antipseudomonal cephalosporins, and the isolation of *S. maltophilia* within 30 days of treatment (Hotta et al. 2014).

Data from a Spanish national survey conducted in 2012 showed that about 20% of bacterial strains isolated from BSI are MDR, 20% of which are carbapenem-resistant (Pena et al. 2012a, b). Therefore, the aim of this study was to analyze the molecular epidemiology and resistance profiles of Gram-negative non-fermentative bacteria isolated from patients hospitalized as a result of BSI in southern Poland, with a particular focus on multi-locus sequence typing (MLST).

## Material and methods

### Study population

Consecutive, non-repetitive non-fermentative Gram-negative bacillus isolates from hospitalized patients with BSI were collected from seven hospitals in southern Poland. Patients were hospitalized in three intensive care units, four internal, and three nephrology or urology wards. Isolates were received by the Department of Microbiology at Jagiellonian University Medical College, and were collected in collaboration with two other laboratories 1st January and 31st December, 2013. Patient information, including age, sex, and place of hospitalization, was also collected.

This work was approved by the Bioethics Committee of Jagiellonian University Medical College (no. KBET/312/B/2012 and KBET/362/B/2012). All data were anonymized prior to analysis.

### Bacterial isolates

Specimens were collected from patients at the onset of symptoms of infection. During the study period, microbiological examination was performed on 1721 blood samples, with a total of 53 Gram-negative non-fermentative isolates tested. Identification of microorganisms was performed using the semi-automated Phoenix system (Becton-Dickinson Diagnostic System, France) according to standard methods.

### Susceptibility testing

Susceptibility testing was performed using the semi-automated Phoenix NMIC/ID-204 system (Becton-Dickinson) according to the manufacturer’s instructions. Antimicrobial susceptibility was assessed according to the current European Committee on Antimicrobial Susceptibility Testing guidelines (EUCAST clinical breakpoint tables v. 5.0; http://www.eucast.org/clinical_breakpoints/, accessed 25.09.2015) and the results were considered as resistant (R) and susceptible (S). Resistant and intermediate strains were grouped together as drug resistant. For ampicillin-sulbactam and tetracycline, antimicrobial susceptibility was assessed according to the Clinical Laboratory Standards Institute guidelines.

For *P.aeruginosa* 13 antimicrobials were tested (gentamicin, tobramycin, amikacin, netilmicin, imipenem, meropenem, ciprofloxacin, levofloxacin, piperacillin/tazobactam, ceftazidime, cefepime, aztreonam, colistin). For *A.baumannii* 15 antimicrobials were tested (gentamicin, tobramycin, amikacin, netilmicin, imipenem, meropenem, ciprofloxacin, levofloxacin, piperacillin/tazobactam, ceftazidime, cefepime, trimethoprim/sulfamethoxazole, ampicillin/sulbactam, colistin, tetracycline). For *S. maltophilia* only trimethoprim-sulfamethoxazole and ceftazidime were tested. (Supplementary Table S1).

Different patterns of resistance, including extensively drug resistant (XDR) and multi-drug resistant (MDR) were defined according to Magiorakos et al. (2012): (1) bacteria non-susceptible to at least one agent in three or more antimicrobial categories were considered as MDR strains; (2) bacteria non-susceptible to at least one agent in all antimicrobial categories, except two or fewer categories were considered as XDR strains.

### Multi-locus sequence typing

MLST was carried out as described previously using 44 strains representing the three most commonly isolated species: *A.baumannii* (Diancourt et al. 2010), *P.aeruginosa* (Curran et al. 2004), and *S.maltophilia* (Kaiser et al. 2009). Sequencing was performed by Genomed SA (Warsaw, Poland), and the resulting sequences were analyzed using ChromasPro 1.4 software (Technelysium Pty Ltd., Australia). MLST data were then compared with the database (http://pubmlst.org) to identify sequence types.

### Pulsed field gel-electrophoresis

Analysis of the genetic similarity between *P. aeruginosa* strains or between *S. maltophilia* strains (separately within species) was performed using pulsed field gel electrophoresis (PFGE) in accordance with a previously published protocol (Shueh et al. 2013). Restriction enzyme digestion of *P. aeruginosa* strains was performed with 25 U of *Spe* I enzyme in Tango buffer and *S. maltophilia* strains were digested by *Xba* I enzyme in Tango buffer (Thermo-Scientific, USA). Electrophoresis was conducted in a CHEFIII PFGE unit applying the parameters: for *P.aeruginosa*- initial pulse 1 s, final pulse 35 s, run time 23 h, voltage 6 V/cm; for *S.maltophilia*- block 1: initial pulse 1 s, final pulse 15 s, run time 10 h, voltage 6 V/cm, block2: initial pulse 15 s, final pulse 60s, run time 11 h, voltage 6 V/cm. Gel Compar II 6.5 (AppliedMaths, Belgium) was used for cluster analysis using the Dice coefficient and unweighted pair group method with arithmetic mean.

Isolates with more than 90% similarity were clustered together as identical.

### Repetitive PCR (rep-PCR)

Epidemiological typing of *A. baumannii* strains from ICUs was performed by repetitive-PCR typing using DiversiLab system (bioMerieux, France) as previously described (Chmielarczyk et al. 2016). Isolates that clustered ≥91.3% were considered related. Results of *A.baumannii* typing were already published.

## Results

Of the 53 patients with BSI, 40 (74.1%) were men. The median age was 64 years (1st and 3rd quartile:40 and 75 years, respectively). The majority of patients were hospitalized in intensive care units (*n* = 30, 56.5%), followed by nephrology or urology wards (*n* = 14, 26.5%) and internal medicine wards (*n* = 9, 17.0%).

*A. baumannii* was the most frequently (*n* = 22, 41.5%), followed by *P. aeruginosa* (*n* = 11, 20.7%) and *S. maltophilia* (n = 11, 20.7%). Other species identified at much lower frequencies included *Achromobacter denitrificans* (*n* = 5, 9.4%), *Acinetobacter lwoffii* (n = 1, 1.9%), *Acinetobacter ursingii* (n = 1, 1.9%), *Comamonas testosterone* (n = 1, 1.9%), and *Ochrobactrum anthropi* (n = 1, 1.9%). The prevalence rates of the non-fermentative Gram-negative bacterial species in blood were: *A. baumannii*, 1.3%; *P. aeruginosa*, 0.6%; *S. maltophilia*, 0.6%; and others (pooled), 0.4%. In addition, multiple pathogens were isolated from the blood samples of five patients. These samples contained: *A.baumannii* together with coagulase-negative *Staphylococcus*; *A.baumannii* together with *Enterococcus faecium*; *A. ursingii* together with *S.maltophilia*; *S.maltophilia* together with coagulase-negative *Staphylococcus*; and *C. testosterone* together with *Achromobacter denitrificans*.

The vast majority of *A.baumannii* isolates (*n* = 19, 86.3%) were XDR, while the remaining isolates were MDR. Fourteen *A.baumannii* strains (63.6%) were resistant to all tested antibiotics except colistin. Among the *P. aeruginosa* isolates, only one was XDR, one was MDR, and all remaining isolates were susceptible to the tested antimicrobials. All *S.maltophilia* isolates were susceptible to sulfamethoxazole/trimethoprim and resistant to ceftazidime. Detailed results of resistance are in Supplementary Table S1.

MLST revealed that all 22 *A.baumannii* strains from the BSI samples belonged to sequence type (ST) 2. Seventeen of these isolates came from one hospital ward. The *P.aeruginosa* isolates belonged to 10 different STs. Only four *S.maltophilia* isolates matched STs present in the database (ST4, ST15, ST116, ST142), with all other isolates showing novel sequence types. Five of these isolates had an identical allele pattern, four of which were isolated from patients on a single unit (Table 1). The detailed nucleotide sequences that were obtained by MLST, are a part of the Supplementary Material.

**Table 1:** Alleles and sequence types Gram negative non-fermentative strains

Species and strain number	Ward	Alleles							Sequence type (ST)
*Acinetobacter baumannii*		*cpn60*	*fusA*	*gltA*	*pyrG*	*recA*	*rplB*	*rpoB*	
17 isolated strains	ICU1	2	2	2	2	2	2	2	2
2 isolated strains	ICU2	2	2	2	2	2	2	2	2
No. 53	Urology1	2	2	2	2	2	2	2	2
No. 480	Urology2	2	2	2	2	2	2	2	2
No. 835	Internal1	2	2	2	2	2	2	2	2
*Pseudomonas aeruginosa*		*acs*	*aro*	*gua*	*mut*	*nuo*	*pps*	*trp*	
No. 17	Urology1	3	104	4	36	71	44	53	27
No. 253	Urology1	38	11	3	13	1	2	4	235
No. 408	ICU1	17	5	12	3	14	4	7	244
No. 429	ICU1	114	73	32	3	4	50	1	396
No. 520	Internal2	22	20	11	3	3	3	7	348
No. 725	ICU1	31	5	57	13	1	40	3	137
No. 764	ICU1	28	3	94	13	4	1	10	644
No. 779	ICU1	14	5	10	7	4	13	7	260
No. 783	ICU1	4	4	12	16	1	6	3	253
No. 786	ICU1	4	4	12	16	1	6	3	253
No. 892	ICU1	17	3	5	4	4	4	3	966
*Stenotrophomonas maltophilia*		*atpD*	*gapA*	*guaA*	*mutM*	*nuoD*	*ppsA*	*recA*	
No. 3	Urology1	76	68	7	7	80	93	74	novel 1
No. 14	Internal3	76	68	7	7	80	93	74	
No. 19	Urology1	76	68	7	7	80	93	74	
No. 22	Urology1	76	68	7	7	80	93	74	
No. 25	ICU3	80	89	43	73	72	98	79	142
No. 129	Urology1	76	68	105	67	80	93	74	novel 2
No. 318	Urology1	3	1	84	58	25	82	6	116
No. 327	Urology1	76	68	7	7	80	93	74	novel 1
No. 435	ICU1	6	1	39	19	76	33	78	novel 3
No. 6840	ICU1	1	4	7	7	28	19	6	4
No. 11865	ICU1	10	29	21	21	32	32	10	15

**Fig. 1:**
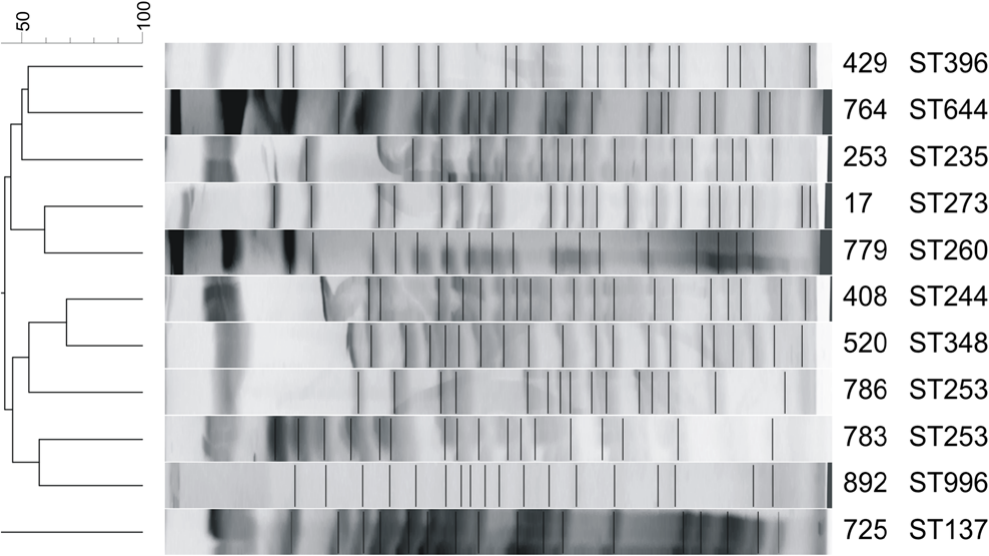
Pulsed-field gel electrophoresis dendrogram of *Stenotrophomonas maltophilia* strains generated by Gel Compar II software (Dice coefficient, tolerance 0%, optimization 1%) together with sequence types (ST)

**Figure 2.**
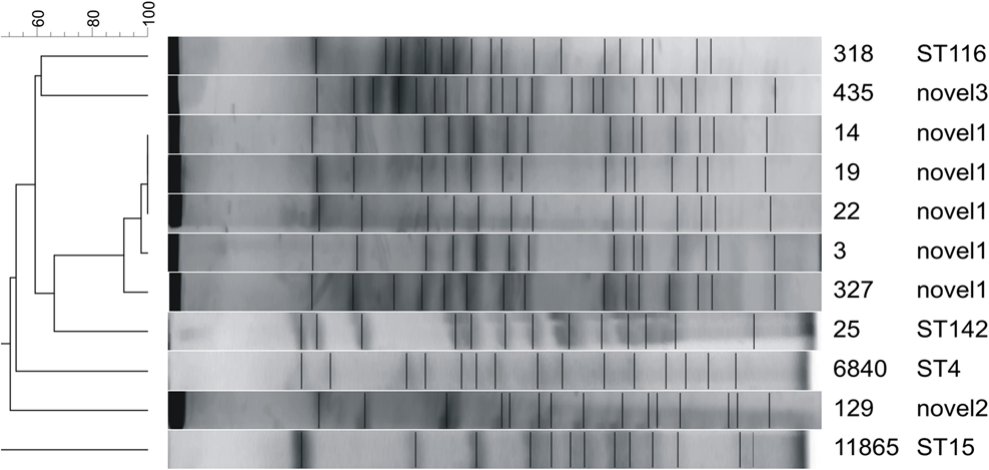
Pulsed-field gel electrophoresis dendrogram of *Pseudomonas aeruginosa* strains generated by Gel Compar II software (Dice coefficient, tolerance 0%, optimization 1%) together with sequence types (ST).

PFGE typing of *P.aeruginosa* strains has shown very high heterogenity among the strains. Pulsotypes showed similarity between 50 and 65%. Two strains of *P. aeruginosa* with the same sequence type (ST253) were characterized by different pulsotypes (Fig. 1). It was different in case of *S.maltophilia* strains, where 5 strains showed similarity at the level of 90%. These strains had also the same allele pattern in the MLST method and, what is more, four of them were derived from the patients hospitalized on the same single unit (Fig. 2).

In rep-PCR sixteen strains of *A.baumannii* (72.7%) belonged to European clone II (EUII) assigned, three strains belonged to other clones and 3 strains have not been tested (only strains from ICU were been tested). This data was previously published (Chmielarczyk et al. 2016).

## Discussion

The prevalence of BSI caused by non-fermentative Gram-negative bacilli has increased in recent years (Rattanaumpawan et al. 2013). *A. baumannii* and *P. aeruginosa* remain the predominant species, but other species are now frequently being identified (Livermore et al. 2008; Vidal et al. 2003). This may be the result of better identification techniques, or changes in the local epidemiology in the hospital wards.

The dissemination of carbapenem-resistant *A. baumannii* in hospitals worldwide is understood as a clonal process: the strains are transmitted through patients and it may cause outbreaks (Munoz-Price et al. 2010). The remarkably high prevalence of *A.baumannii* is partly the result of clonal spreading, as all isolated strains in the current study belonged to ST2. We have previously confirmed (by rep-PCR with DiversiLab system) the dominance in southern Poland of international clone 2,which is ubiquitous in Europe (Chmielarczyk et al. 2016). Epidemiological studies are important for monitoring XDR *A. baumannii* strains. Apart from such typing methods as MLST and rep-PCR, whole genome sequencing (WGS) is the promising technique for strain typing and outbreak investigation. WGS seems to be more discriminatory than band-based techniques however still is very expensive, and requires an additional person skilled in bioinformatics (Fitzpatrick et al. 2016).

Of serious concern is the phenotype of this our clone, with strains of this type mainly only susceptible to colistin. Unfortunately, we have no data regarding the mortality rates of patients, nor the use of central venous catheters, which is needed for more in-depth study of XDR *A. baumannii*. In the ICU the most frequent empirically prescribed antibiotics are carbapenems. The major limitation of using tygecycline in treatment of BSIs is the low concentration that this substance achieves in serum. Therefore, the most adequate in salvage therapy in the case of XDR *A.baumannii* or *P.aeruginosa* would be colistin in monotherapy or in combination therapy with other agents (Martis et al. 2014; Dimopoulos et al. 2015).

*P. aeruginosa* was isolated from BSI in the current study at a frequency only half that of *A. baumannii*, which is not consistent with other studies: *P. aeruginosa* is generally more common than *A. baumannii* (Picot-Guéraud et al. 2015; Sligl et al. 2015; Hotta et al. 2014). There was also a higher level of diversity among the *P. aeruginosa* isolates, with only two isolates showing the same ST (ST253), both of which were isolated from patients hospitalized on the same ward. Only one *P. aeruginosa* isolate belonged to globally disseminated XDR ST235 clone type. The PFGE method confirmed the genetic diversity among *P. aeruginosa* strain*s*.

Our study also identified a relatively high number of *S. maltophilia* (20.7% of the total number of isolates) and *A. denitrificans* (9.4%) isolates. These prevalence rates were much higher than in a previous study by Rattanaumpawan et al. (5.4% and 0%, respectively), but similar to results obtained by Hotta et al. (*S. maltophilia* prevalence of 18.6%). Five of the *S. maltophilia* isolates had the same novel ST, and four of these isolates came from the same ward. What is more, they were characterized by identical pulsotypes according to PFGE method. We therefore assume that horizontal transmission was responsible for these infections. All *S. maltophilia* isolates were susceptible to sulfamethoxazole/trimethoprim, but some previous studies have suggested that levofloxacin should be used as an alternative treatment of BSI caused by *S. maltophilia* (Cho et al. 2014).

Interestingly, while other Gram-negative bacillus species were identified as the cause of BSI in the current study, none of these isolates were MDR. *A. denitrificans* rarely causes infectious disease in humans, and is mainly associated with immunocompromised patients.

An outbreak caused by *C. testosteroni* affecting hematological patients resulted from contamination of a disinfectant dispenser in a hospital in France in 2007 have been reported (Siebor et al. 2007), but no other infectious outbreaks caused by *C. testosteroni* have been reported. In addition, there is currently no MLST protocol for this species.

Analysis of the local epidemiology and antimicrobial susceptibility patterns of bacteria causing BSI may aid in the development of empiric therapy guidelines, infection surveillance programs, and improved patient outcomes.

## Conclusions

Non-fermentative Gram-negative bacilli are now one of the most important causes of severe infections in Polish hospitals. *Acinetobacter* species are more commonly isolated from BSI in Poland than in other countries, which is of serious concern because of the high prevalence of multi-drug resistance among *A. baumannii* strains. Treatment options are severely limited for these bacteria, which are a known risk factor for increased patient mortality. The MLST technique (as well as rep-PCR) allows confirmation of horizontal transfer of *Acinetobacter* strains, and may aid in the identification of a common source of infection. Among *P. aeruginosa*, both typing methods (MLST and PFGE) showed the genetical variety of strains. While there is no one method guaranteed to reduce the number of infections caused by Gram-negative bacilli, in the case of *Acinetobacter*, appropriate infection control and hand hygiene may be effective.

## Electronic supplementary material

Supplementary Table S1 (DOCX 34 kb)

ESM 1 (DOCX 14 kb)

ESM 2 (DOCX 39 kb)

ESM 3 (DOCX 31 kb)
