# Supplementary material for: Multi-locus sequence typing (MLST) of non-fermentative Gram-negative bacilli isolated from bloodstream infections in southern Poland
Source: Folia Microbiol (Praha). 2017 Sep 22;63(2):191–6. doi: 10.1007/s12223-017-0550-7 (PMC5805803; doi:10.1007/s12223-017-0550-7)
Supplement: Supplementary file 1 — (DOCX 34 kb) [file 12223_2017_550_MOESM1_ESM.docx]

Supplementary Table. Antimicrobial resistance among Gram negative non-fermentative strains.

| **Acinetobacter baumannii** | | | |  |  |  |  |  |  |  |  |  |  |  |  |  |  |  |  |  |  |  |
| --- | --- | --- | --- | --- | --- | --- | --- | --- | --- | --- | --- | --- | --- | --- | --- | --- | --- | --- | --- | --- | --- | --- |
| **No** | **ward** | **patients age** | **sex** | **MLST** | **gentamicin** | **tobramycin** | **amikacin** | **netilmicin** | **imipenem** | **meropenem** | **ciprofloxacin** | **levofloxacin** | **piperacillin/ tazobactam** | **ceftazidime** | **cefepime** | **trimethoprim/ sulfamethoxazole** | **ampicillin/ sulbactam** | **colistin** | **tetracycline** | **colistin MIC** | **MDR or XDR** | **DiversiLab** |
| 53 | urology 1 | 24 | F | ST2 | R | R | R | R | S | S | R | R | S | R | R | R | S | S | R | 0.5 | MDR | nd |
| 156 | ICU 1 | 81 | F | ST2 | R | R | R | R | R | R | R | R | R | R | R | R | R | S | R | 0.5 | XDR | EUII |
| 306 | ICU 1 | 94 | F | ST2 | R | R | R | R | R | R | R | R | R | R | R | R | S | S | R | 0.38 | XDR | EUII |
| 308 | ICU 1 | 58 | M | ST2 | R | R | R | R | R | R | R | S | R | R | R | R | R | S | R | 0.5 | XDR | EUII |
| 374 | ICU 1 | 81 | M | ST2 | R | R | R | R | R | R | R | R | R | R | R | R | R | S | R | 1 | XDR | EUII |
| 388 | ICU 1 | 33 | M | ST2 | R | R | R | R | R | R | R | R | R | R | R | R | R | S | R | 0.38 | XDR | other clone |
| 411 | ICU 1 | 86 | F | ST2 | R | R | R | R | R | R | R | R | R | R | R | R | R | S | R | 0.38 | XDR | EUII |
| 415 | ICU 1 | 39 | M | ST2 | R | R | R | R | R | R | R | R | R | R | R | R | R | S | R | 0.75 | XDR | EUII |
| 422 | ICU 1 | 64 | M | ST2 | R | R | R | R | R | R | R | R | R | R | R | R | R | S | R | 1 | XDR | EUII |
| 440 | ICU1 | 75 | F | ST2 | R | R | R | R | S | S | R | R | R | R | R | R | S | S | R | 1 | MDR | EUII |
| 480 | urology 2 | 43 | F | ST2 | S | R | R | R | R | R | R | R | R | R | R | R | R | S | R | 0.25 | XDR | nd |
| 741 | ICU1 | 80 | F | ST2 | R | R | R | R | R | R | R | R | R | R | R | R | R | S | R | 1 | XDR | EUII |
| 744 | ICU1 | 84 | M | ST2 | R | R | R | R | R | R | R | R | R | R | R | R | R | S | R | 1 | XDR | EUII |
| 745 | ICU1 | 41 | M | ST2 | R | R | R | R | R | R | R | R | R | R | R | R | S | S | R | 1 | XDR | EUII |
| 767 | ICU1 | 70 | M | ST2 | R | R | R | R | R | R | R | R | R | R | R | R | R | S | R | 0.75 | XDR | EUII |
| 770 | ICU1 | 73 | F | ST2 | R | S | S | R | S | R | R | R | R | R | R | R | S | S | R | 1 | MDR | EUII |
| 780 | ICU1 | 42 | M | ST2 | R | R | R | R | R | R | R | R | R | R | R | R | R | S | R | 1 | XDR | other clone |
| 835 | internal 1 | 84 | M | ST2 | R | R | R | R | R | R | R | R | R | R | R | R | R | S | R | 1 | XDR | nd |
| 896 | ICU1 | 42 | M | ST2 | R | R | R | R | R | R | R | R | R | R | R | R | R | S | R | 0.5 | XDR | other clone |
| 898 | ICU1 | 64 | M | ST2 | R | R | R | R | R | R | R | R | R | R | R | R | R | S | R | 1.5 | XDR | EUII |
| 926 | ICU 2 | 31 | M | ST2 | R | R | R | R | R | R | R | R | R | R | R | R | R | S | R | 1 | XDR | EUII |
| 932 | ICU 2 | 78 | M | ST2 | R | R | R | R | R | R | R | R | R | R | R | R | S | S | R | 1 | XDR | EUII |

| **Pseudomonas aeruginosa** | | | |  |  |  |  |  |  |  |  |  |  |  |  |  |  |  |  |  |
| --- | --- | --- | --- | --- | --- | --- | --- | --- | --- | --- | --- | --- | --- | --- | --- | --- | --- | --- | --- | --- |
| **No** | **ward** | **patients age** | **sex** | **MLST** | **gentamicin** | **tobramycin** | **amikacin** | **netilmicin** | **imipenem** | **meropenem** | **ceftazidime** | **cefepime** | **ciprofloxacin** | **levofloxacin** | **piperacillin/ tazobactam** | **aztreonam** | **colistin** | **colistin MIC** | **MDR or XDR** | **MBL** |
| 17 | urology 1 | 69 | F | ST273 | S | R | S | S | S | S | S | S | S | R | S | S | S | 1 |  |  |
| 253 | urology 1 | 65 | M | ST235 | R | R | R | R | S | S | R | R | R | R | R | R | S | 1 | XDR |  |
| 408 | ICU 1 | 42 | M | ST244 | S | S | S | S | R | R | S | S | S | S | R | S | S | 1.5 |  | MBL |
| 429 | ICU 1 | 39 | M | ST396 | S | S | S | S | S | S | S | S | S | S | S | S | S | 1 |  |  |
| 520 | Internal 2 | 3 | M | ST348 | S | S | S | S | S | S | S | S | S | S | S | S | S | 1.5 |  |  |
| 725 | ICU 1 | 88 | F | ST137 | S | S | S | S | S | S | S | S | S | S | S | S | S | 1 |  |  |
| 764 | ICU 1 | 49 | M | ST644 | S | S | S | S | S | S | S | S | S | S | S | S | S | 1.5 |  |  |
| 779 | ICU 1 | 53 | M | ST260 | R | R | R | R | S | S | R | R | R | R | S | R | S | 1.5 | MDR |  |
| 783 | ICU 1 | 71 | M | ST253 | S | S | S | S | S | S | S | S | S | S | S | S | S | 1.5 |  |  |
| 786 | ICU 1 | 70 | M | ST253 | S | S | S | S | S | S | S | S | S | S | S | S | S | 1.5 |  |  |
| 892 | ICU 1 | 53 | M | ST966 | S | S | S | S | R | R | S | S | R | S | S | S | S | 2 |  | MBL |

| **Stenotrophomonas maltophilia** | | | | |  |  |
| --- | --- | --- | --- | --- | --- | --- |
| **No** | **ward** | **patients age** | **sex** | **MLST** | **trimethoprim/ sulfamethoxazole** | **ceftazidime** |
| 3 | urology 1 | 88 | M | 76;68;7;7;80;93;74 | S | R |
| 14 | internal 3 | 92 | M | 76;68;7;7;80;93;74 | S | R |
| 19 | urology 1 | 85 | M | 76;68;7;7;80;93;74 | S | R |
| 22 | urology 1 | 29 | F | 76;68;7;7;80;93;74 | S | R |
| 25 | ICU 3 | 72 | M | ST142 (80;89;43;73;72;98;79) | S | R |
| 129 | Urology 1 | 77 | M | 76;68;105;67;80;93;74 | S | R |
| 318 | Urology 1 | 47 | M | ST116 (3;1;84;58;25;82;6) | S | R |
| 327 | urology 1 | 52 | M | 76;68;7;7;80;93;74 | S | R |
| 435 | ICU 1 | 22 | M | 6;1;39;19;95;33;78 | S | R |
| 6840 | ICU 1 | 67 | M | ST4 1;4;7;7;28;19;6 | S | R |
| 11865 | ICU 1 | 74 | M | ST15 (10;29;21;21;32;32;10) | S | R |

Legend: ICU- intensive care unit, MLST- multi locus sequence type, ST- sequence type, F- female, M-male, S-susceptible, R-resistant, MDR- multidrug resistant, XDR- extensively drug resistant, MBL- metallo –beta-lactamase, MIC- minimal inhibitory concentration, EUII – European clone II.
