# Supplementary material for: Multi-locus sequence typing (MLST) of non-fermentative Gram-negative bacilli isolated from bloodstream infections in southern Poland
Source: Folia Microbiol (Praha). 2017 Sep 22;63(2):191–6. doi: 10.1007/s12223-017-0550-7 (PMC5805803; doi:10.1007/s12223-017-0550-7)
Supplement: Supplementary file 2 — (DOCX 14 kb) [file 12223_2017_550_MOESM2_ESM.docx]

**Nucleotide sequences of *Acinetobacter baumannii***

**Strain no. 53, 156, 306, 308, 374,388, 411, 415, 422, 440, 480, 741, 744, 745, 767, 770, 780, 835, 896, 898, 926, 932 - all ST2**

*cpn60 allel 2*

CTCAAGCAATTTTAAATGAAGGTATCAAATCAGTAACTGCTGGTATGAACCCAATGGATTTAAAACGCGGTATCGACATTGCAGTAAAAACTGTAGTTGAAAATATCCGTTCTATTGCTAAACCAGCTGATGATTTCAAAGCAATTGAACAAGTAGGTTCAATCTCTGCTAACTCTGATACTACTGTTGGTAAACTTATTGCTCAAGCAATGGAAAAAGTAGGTAAAGAAGGCGTAATCACTGTAGAAGAAGGCTCAGGCTTCGAAGACGCATTAGACGTTGTAGAAGGTATGCAGTTTGACCGTGGTTATATCTCTCCGTACTTTGCAAACAAACAAGATACTTTAACTGCTGAACTTGAAAATCCGTTCATCCTTCTTGTTGATAAAAAAATCAGCAACATTCGTGAATTGATTTCTGTTTTAGAAGCAGTTGCTAAAACTGGTAAACCACTTCTT

*fusA allel 2*

ATGCAGGTAAAACAACTACAACTGAACGTATTTTGTTCTACACAGGTGTATCTCACAAAATTGGTGAAGTACACGACGGTGCAGCAACAATGGACTGGATGGAACAAGAGCAAGAGCGTGGTATTACAATTACCTCTGCTGCAACAACTTGTTTCTGGTCTGGTATGGGTAACCAATTCCCACAACACCGTATCAACGTAATTGATACACCGGGACACGTTGACTTCACAATCGAAGTTGAGCGTTCTATGCGTGTTCTTGACGGTGCTTGCATGGTTTACTGTGCAGTTGGTGGTGTACAGCCTCAGTCTGAAACTGTATGGCGTCAGGCTAACAAATATAAAGTGCCTCGTTTAGCATTCGTGAACAAGATGGACCGTACTGGTGCAAACTTCTTCCGTGTTGTTGAACAAATGAAAACACGTCTTGGTGCGAATCCTGTGCCAATCGTTGTGCCAATCGGTGCTGAAGACACATTCACTGGTGTAGTTGACCTTATCGAAATGAAGGCAATTATCTGGGATGAAGCTTCTCAAGGTATGAAGTTTGAATACGGCGAGATTCCAGCTGACCTAGTTGATACTGCTCAAGAATGGCGTACAAACATGGTTGAAGCTGCTGCTGAAGCTTCTGAAGAGTTAATGGACAAATACCTTGAAGAGGGTGATCTTTCTAAAGAAGACATCATCGCAGGTTTACGTGCTCGTACATTAGCTTCTGAAATCCAAGTAATGCTTTGTGGTTCTGCATTCAAGAACAAAGGTGT

*glt allel 2*

GCACATTAGGTCCCGATGTAATCGACGTTAAAGATGTATTGGCCTCAGGTCACTTTACTTTTGATCCTGGTTTTATGGCGACAGCTTCATGCGAGTCTAAAATCACATTTATCGATGGTGACAAAGGTATTTTATTACACCGCGGTTACCCGATTGACCAGTTAGCGACTCAAGCAGACTACCTTGAAACTTGTTATTTATTATTAAATGGCGAGTTACCAACTGCTGAACAAAAAGTTGAGTTCGATGCGAAAGTTCGTGCTCATACTATGGTTCATGATCAAGTTAGCCGTTTCTTCAATGGTTTCCGTCGTGATGCTCACCCTATGGCAATCATGGTTGGTGTAGTAGGCGCATTATCTGCTTTCTATCACAACAACCTTGACATTGAAGACATCAACCACCGCGAAATTACTGCGATTCGTTTGATTGCTAAAATTCCAACGCTTGCTGCTTGGAGCTACAAATATACTGTAGGTCAGCCATTCATCTATCCACGTAATGACTTAAATTACGCGGAAAACTTCTTACACATGATGTTTGCAACTCCTGCAGACCGTGACTACAAAGTAAACCCTGTTCTTGCTCGTGCAATGGATCGTATCTTTACGCTTCATGCTGACCACGAACAAAACGCGTCTACTTCTACAGTTCGACTTGCTGGTTCTACTGGTGCGAACCCATATGCGTGTATCTCTGCTG

*pyrG allel 2*

CATCACTAGGTAAAGGTATTTCTGCTGCTTCTGTTGCTGCACTTTTGGAAGCTCGTGGCTTAAAAGTCACAATGGTTAAAATGGATCCTTATATTAATGTCGATCCAGGGACAATGAGCCCATTCCAGCATGGTGAAGTTTTTGTTACCGAAGATGGTGCAGAAACAGATCTGGATCTGGGTTATTACGAACGTTTCTTACGTCGCGCGAAAATGACCAAACTAAACAACTTCACTAGTGGTCGTGTATATCAAGACGTTTTAAATAAAGAGCGTCGTGGTGATTACTTAGGTGGTACAGTTCAGGTTATTCCTCATATTACCGACAATATTAAAGAACGTGTACTCCGCGCGGGCGAAGGCTATGATGTTGCTATCGTAGAGATCGGTGGTACCGTGGGTGACATCGA

*recA allel2*

TGGTAAAACTACAATGACATTGCAAGCAATTGCTCAATGTCAAAAATCTGGTGGTACATGTGCCTTCATTGATGCTGAGCACGCCCTAGACCCTCAATATGCACGCAAACTTGGTGTAGATATTGATAACCTACTTGTTTCACAACCCGACAACGGTGAGCAAGCACTTGAAATTGCTGACATGCTTGTCCGTTCAGGCGCAATTGATTTAATCGTTGTGGACTCGGTAGCTGCACTTACCCCTAAAGCAGAAATCGAAGGTGAGATGGGTGACTCTCATATGGGTCTACAAGCACGTCTTATGAGCCAGGCACTTCGTAAAATTACGGGTAATGCTAAACGTTCAAACTGTATGGTTATCTTCATTAACCAGATTCGTATGAAAATTGGTGTAATGTTTGGCA

*rplB allel 2*

TTGAATACGATCCTAACCGTACAGCGCATATTGCATTATTGAAATATGCTGACGGTGAGCGTCGTTATATCATTGCGCCTAAAGGCTTACGTGCTGGTGATAAAGTACAATCTGGTAACGATGCTCCAATTCGTCCAGGTAACTGTTTACCACTTCGTAACATGCCAATCGGTTCTACACTTCATAACGTTGAACTTAAAATCGGTAAAGGTGCTCAATTAGCACGTTCTGCTGGTGCTTCAGTTCAATTGTTGGGTCGTGATGGTTCTTACGCAATCATTCGTCTTCGTTCAGGCGAAATGCGTAAAGTACACGTTGAATGCCGCGCTGTAATTGGTGAAGTTTCTAACCAAGAAAACAACCTTCGCTCATTAGGTAAAGCTGGTGCTGCACGCTGGCGTGGTGTTCGTCCTACCGTACGTGGTATGGCGATGAACCCGATTGATCACCCGC

*rpoB allel 2*

AAGCCAAGCAGAAACAGATAACTTGTCTCCACAAGATTTGATTAATGCAAAACCAGTTGCTGCTGCAATCAAAGAATTCTTTGGTTCAAGCCAGTTATCTCAGTTTATGGACCAAAACAACCCATTATCTGAGATTACACATAAACGTCGTGTATCTGCGCTTGGTCCTGGTGGTTTAACACGTGAACGTGCAGGCTTCGAAGTACGTGACGTACACCAAACTCACTATGGTCGTGTTTGTCCAATTGAAACTCCTGAAGGTCCAAACATTGGTTTGATCAACTCGCTTTCTGTATACGCAAAAGCGAATGACTTCGGTTTCTTGGAAACACCATACCGCAAAGTTGTAGATGGTCGTGTAACTGATGATGTTGAATATTTATCTGCAATTGAAGAAGTAGGTACTGTTATTGCACAGGCCGACTCTGCTGTAGATAAAGATGGCAACTTAACAGAAGAATTCGTTTCTGTTCGTCATCAAGGTGAATTCGTACGTATGCCGCCTGAAAAAGTAACGCATATGGACGTTTCTGCACAGCAGGTAGTATCTGTTGCTGCATCACTTATTCCATTCCTTGAACACGATGACGCAAACCGTGCGCTCATGGGTTCAAACATGCAACGTCAGGCAGTTCCTACTTTACGTGCGGATAAACCGCTTGTAGGTACAGGTATGGAAGCGAACGTTGCACGTGACTCTGGTGTGTGTGTAATCGCAAACCGTGGCGGTGTAATTGAATATGTAGATGCTTCTCGTATCGTTATTCGTGTAAACGAAGATGAAATGGTTGCAGGTGAGGCAGGTGTAGATATCTATAACCTCATCAAATATACGCGTTCAAACCAAAATACTTGTATTAACCAAAATGTTATCGTGAACTTGGGCGACAAAGTTGCTCGTGGTGACATCTTGGCAGACGGTCCGTCAACAGACATGGGTGAAC
