# Supplementary material for: Multi-locus sequence typing (MLST) of non-fermentative Gram-negative bacilli isolated from bloodstream infections in southern Poland
Source: Folia Microbiol (Praha). 2017 Sep 22;63(2):191–6. doi: 10.1007/s12223-017-0550-7 (PMC5805803; doi:10.1007/s12223-017-0550-7)
Supplement: Supplementary file 3 — (DOCX 39 kb) [file 12223_2017_550_MOESM3_ESM.docx]

**Nucleotide sequences of Pseudomonas aeruginosa**

**Strain no. 17 ST 273**

*asc allel 104*

TGCCCCTTGATGTCGTGCTGCATGCCGACCACCGCCGCCTCGGCGACCTTCGGGTGGGCGACCATCGCGCTTTCCACCTCGGCGGTGCCCATGCGGTGGCCGGAGACGTTGAGCACGTCGTCGACCCGCCCGGTGATCCAGTAGTAGCCGTCCTCGTCGCGGCGCGCGCCGTCGCCGGTGAAGTACATGCCCTTGAAGGTCTTGAAGTAGGTGTCGACGAAGCGGTCATGGTCGCCGAACAGGGTCCGCGCCTGGCCCGGCCAGGAGTCGAGGATCACCAGGTTGCCCTCGGCGGCGCCCTCGATCAGGTTGCCGAGGTTGTCCACCAGTGCCGGGACCACGCCGAAGAACGGCTTGGCCGCGGAGCCCGGCTTCATCGCGTGGGCGCCCGGCAGCGGGGTCATCAGGCAGGCGCCGGTCTCGGTCTGCCACCAGGTGTCGACGATCGGGCAGCGCGACTGGCCGACGGTCTCGTAGTACCACTGCCAGGCTTCCGGGTTGATCGGCTCGCCCACCGAACCGAGCAGACGCAGGCTGGAACCGTCGGCACCGGCCACCGCCGCCTTGCCTTCGGCCATCATCGCGCGGATCGCGGTCGGCGCGGTGTAGAGGATGTTAACCTTGTGCTTGTCGATGATCTTCGCCACGCGGGTCACGTCGGGGTAGTTCGGTACGCCCTCGAACAGAATGGTGGTGGCGCCGTTGGCCAACGGGCCATAGACGATGTAGGTGTGGCCGGTGACCCAGCCGATGTCGGCGGTGCACCAGTAGACTTCGCCCGGACGGTAGTCGAAGACCCGCTCGTGGGTCAGCGAGGCG

*aro allel 4*

GGGGCTATGACTGGAAACCCGAGCCGGGCAGCGAGGAAGCGCGCCTGACCGAGTATTTCCTCGCCGACCGCGATTGGCTCGCCGGCCAGCCCTGACCCGCCCGCAGGGGACGAGTCCGCTCGTCCCGTCCCACCCCGGCAAAGGAACGACCCATGGACCGCTACTGCGTATTCGGCAACCCCATCGGCCACAGCAAGTCGCCGCTGATCCACCGCCTGTTCGCCGAGCAGACCGGCGAGGCGCTGGTCTATGACGCGCAGCTGGCGCCGCTGGACGATTTCCCCGGGTTCGCCCGGCGCTTCTTCGAGCAGGGCAAGGGCGCCAATGTCACCGTGCCGTTCAAGGAAGAGGCCTATCGTCTGGTGGACGAATTGAGCGAGCGGGCCACCCGGGCCGGGGCGGTGAACACCCTGATCCGCCTGGCCGACGGTCGCCTGCGCGGCGACAACACCGACGGCGCGGGCTTGCTGCGGGACCTGACGGCGAACGCCGGGGTCGAGCTGCGCGGCAAGCGGGTTCTCCTGCTCGGCGCCGGCGGTGCGGTGCGCGGGGTGCTCGAACCCTTCCTCGGCGAGTGCCCGGCGGAGTTGCTGATCGCCAACCGCACGGCGCGGAAGGCCGTGGACCTGGCCGAGCGATTCGCCGATCTCGGCGCGGTGCGCGGCTGCGGTTTCGCCGAGGTCGAAGGGCCTTTCGACCTGGTCGTCAACGGCACCTCGGCCAGTCTTGCCGGCGACGTGCCGCCGCTGGCGCAGAGCGTGATCGAGCCCGGCCGTACCGTCTGCTACGACATGATGTATGCCAAGGAACCGACCGCCTTCAACCGCTGGGCCGCCGAACGCGGTGCGGCGCGTACCCTGGATGGCCTGGGCATGCTGGTGGAGCAGGCCGCCGAGGCATTCTTCCTCTGGCGCGGCGTGCGTCCTGCCTCGGCGCCAGTGTTGGAGACGCTGCGCCGACAGTTGGCAACTGTCTGAGTTGTCAGGCGGCATTTGAGACGTACGTCTCAAGTTGGCTTCTATCTGACATGTAGGAATCACAAAACCGGG

*gua allel 36*

GGTGGATGAGCCACGGCGACAAGGTCACCGAGATGCCGGCCGGCTTCCACATCCTGGCCAGCACCCCGAGCTGCCCGATCGCCGCCATGGCCGACGATGCCCGCGCCTACTACGGCGTGCAATTCCACCCGGAAGTCACCCACACCAAGCAGGGCCTGCGCATTCTCTCGCGCTTCGTCCTCGACATCTGCGGTTGCGCCGCGCTGTGGACCCCCTCGAACATCGTCGACGACGCCATCGCCACCGTGCGCGCCCAGGTCGGTTCCTCCAAGGTCCTGCTAGGCCTCTCCGGCGGCGTGGACTCCTCGGTGGTCGCCGCGCTGCTGCACAAGGCCATCGGCGACCAACTGACCTGCGTGTTCGTCGACAACGGCCTGCTGCGCCTGCACGAAGGCGACCAGGTGATGGCCATGTTCGCCGAGAACATGGGCGTGAAGGTGATCCGCGCCAACGCCGAGGACAAGTTCCTCGGCCGCCTGGCCGGCGTCGCCGACCCGGAAGAGAAGCGCAAGATCATCGGCCGCACCTTCATCGAAGTCTTCGACGAAGAAGCCACCAAGCTGCAGGACGTGAAGTTCCTCGCCCAGGGCACCATCTACCCCGACGTGATCGAGTCGGCCGGCGCCAAGACCGGCAAGGCCCACGTGATCAAGTCGCACCACAACGTCGGCGGCCTGCCGGAGGACATGCAGTTCGAACTGGTCGAGCCGCTGCGCGAACTGTTCAAGGACGAAGTGCGCAAGATCGGCCTGGAGCTGGGCCTGCCCTACGACATGGTCTACCGCCACCCGTTCCCCGGCCCGGGCCTGGGCGTGCGCATCCTCGGCGAGGTGAAGAAGGAGTACGCCGACCTGCTGCGCCAGGCCGACCACATCTTCATCGAGGAACTGCGCGCCTTCGACTGGTACCACAAGACCAGCCA

*mut allel 71*

GTGAGGTGGTCGAGCGTCCCGCCTCGGTGGCCAAGGAACTCCTGGAAAACAGCCTTGACGCCGGTTCCCGGCGCATTGATGTGGAGGTCGAGCAGGGCGGCATCAAGTTGCTGCGAGTGCGCGACGACGGTCGCGGCATCCCCGCCGACGACCTGCCGCTGGCCCTGGCTCGCCACGCCACCAGCAAGATCCGCGAGCTGGAAGACCTGGAGCGGGTGATGAGCCTCGGCTTCCGTGGCGAGGCGCTGGCCTCGATCAGCTCGGTAGCGCGCCTGACCATGACCTCGCGTACCGCCGACGCCGGCGAAGCCTGGCAGGTGGAAACCGAGGGCCGCGACATGCAGCCGCGGGTACAGCCGGCGGCGCACCCGGTGGGGACCAGCGTCGAGGTTCGCGACCTGTTCTTCAACACCCCGGCGCGGCGCAAGTTCCTGCGTGCCGAGAAGACCGAGTTCGACCATCTGCAGGAAGTCATCAAGCGCCTGGCGCTGGCCCGTTTCGACGTGGCTTTCCACCTGCGCCACAACGGCAAGACCATCTTCGCCCTGCACGAGGCGCGAGACGAGCTGGCCCGCGCGCGGCGGGTCGGCGCGGTGTGCGGCCAGGCATTCCTCGAGCAGGCGCTGCCGATCGAGGTCGAGCGCAACGGCCTGCACCTGTGGGGCTGGGTCGGCTTGCCGACCTTCTCCCGCAGCCAGCCGGACCTGCAGTACTTCTATGTGAACGGGCGCATGGTGCGCGACAAGCTGGTCGCCCACGCGGTGCGCCAGGCTTATCGCGACGTGCTGTACAACGGCCGGCATCCGACCTTCGTGCTGTTCTTCGAAGTCGATCCGGCGGTGGTGGACGTCAACGTGCACCCGACCAAGCACGAAGTTCGCTTCCGTGACAGCCGGATGGTCCATGACTTCCTCTATGGCACCC

*nuo allel 19*

ATATAGGTGCCCAGGTACAGCAGGTGGTTCAGGATACGGAAGAACTCCGCCATCATGATCCGGATCACGTCGACCCGCTGGGGCACCTTGATCCCGGCGAGCTTCTCCACCGAGAGTACGTAGGGCAGGTTGTTCATCACCCCGCCGAGGTAGTCGATGCGGTCGGTGTAGGGGATGAAACTGTGCCAGGACTGGCGCTCGGCCATCTTCTCGGCGCCGCGGTGGTGGTAGCCGATCTCCGGGACGCAGTCGATGATCTCCTCGCCGTCCAGTTGCAGGATGATGCGGAACGCGCCGTGGGCGGACGGGTGGTTCGGGCCGAGGTTGAGGAACATGTAGTCCTCGTTCTCGCCGTGGCGCTTCATGCCCCAGTCTTCCGGCTTGAAGCGCAGGGCCTCCTGCTCCAGGTCCTGCTTGGCGGCGGACAGGGAGTAGGGATCGAACTCGGTGGCGCGCGCCGGGTAGTCCTTGCGCAGCGGATGGCCCTGCCAGGTCGGCGGCATCAGCATGCGGGTCAGGTGCGGGTGGCCGGTGAAGGTGATGCCGTACATGTCCCACACTTCGCGCTCGTACCAGTTGGCGTTGGGCCAGTACGGG

*pps allel 4*

GTCGCTCCGGTCAAGGTAGTGGACGTCGATCGTGCCGATCGCGCCCGCTTCGCCCTGAGCGACGCCGAGGTCACCGAGCTGGCCAAGCAGGCCATGATCATCGAGAAGCACTATGGCCGCCCGATGGACATCGAATGGGCCAAGGACGGTGACGACGGCAAGCTGTACATCGTCCAGGCACGCCCGGAAACCGTGAAGAGCCGCGCCAGCGCCACGGTCATGGAGCGCTACCTGCTGAAAGAGAAGGGGACCGTCCTGGTGGAAGGGCGTGCCATCGGCCAGCGCATCGGTGCCGGTCCGGTCAAGGTGATCAACGACGTGTCGGAAATGGACAAGGTCCAACCGGGTGACGTCCTGGTCTCCGACATGACCGACCCGGACTGGGAGCCGGTGATGAAGCGCGCCAGCGCCATCGTCACCAACCGCGGCGGGCGTACCTGCCACGCGGCGATCATCGCTCGCGAACTGGGCATCCCGGCGGTGGTCGGTTGCGGCAACGCCACCCAGATCCTGCAGGATGGCCAGGGGGTGACCGTTTCCTGTGCCGAAGGCGATACCGGCTTCATCTTCGAAGGCGAACTCGGTTTCGATGTGCGCAAGAACTCGGTCGACGCCATGCCCGACCTTCCGTTCAAGATCATGATGAACGTCGGCAATCCCGATCGCGCTTTCGATTTCGCCCAGTTGCCGAACGAAGGCGTGGGCCTGGCCCGCCTCGAATTCATCATCAACCGCATGATCGGCGTGCACCCCAAGGCATTGCTGAACTTCGCCGGTCTGCCGGCGGACATCAAGGAAAGCGTGGAGAAGCGCATCGCCGGCTATCCCGATCCGGTCGGCTTCTACGTCGAGAAGCTGGTGGAAGGCATCAGCACCCTGGCCGCGGCGTTCTGGCCGAAGAAGGTCATCGTGCGCCTGTCCGACTTCAAGTCCAATGAGTACGCCA

*trp allel 53*

GGTCGTGAGCCGGCGTTTCGTGCCAGCTTCACCCGCGAGGACTATGAAAACGCGGTAGGAAGGATCAAGGACTACATCCTGGCCGGCGACTGCATGCAGGTGGTGCCGTCGCAGCGCATGTCCATCGAATTCAAGGCGGCGCCCATCGACCTGTACCGCGCGCTGCGCTGTTTCAATCCGACGCCCTACATGTACTTCTTCAACTTCGGCGACTTCCATGTCGTGGGCAGCTCGCCGGAGGTGCTGGTACGGGTCGAGGATGGCCTGGTGACGGTGCGCCCGATCGCCGGTACCCGTCCGCGCGGGATCAACGAAGAGGCCGACCTGGCGCTGGAGCAGGATCTGCTGTCGGACGCCAAGGAGATCGCCGAGCACCTGATGCTGATCGACCTGGGGCGCAACGACGTGGGGCGGGTGTCCGATATCGGCGCGGTGAAGGTCACCGAAAAAATGGTGATCGAACGTTACTCCAACGTCATGCACATCGTGTCCAACGTCACCGGGCAATTGCGCGAGGGGCTCAGCGCGATGGACGCGCTGCGGGCGATTCTGCCGGCGGGCACTCTATCCGGCGCGCCGAAGATCCGCGCCATGGAGATCATCGACGAACTGGAGCCGGTCAAGCGTGGAGTCTACGGCGGCGCGGTCGGCTACCTGGCATGGAACGGCAACATGGACACCGCCATTGCCATCCGCACCGCGGTGATCAAGAACGGTGAACTCCACGTGCAGGCCGGCGGCGGTATCGTTGCCGACTCGGTGCCCGCGCTGGAGTGGGAAGAAACCATCAACAA

**Strain no. 253 ST 235**

*acs allel 38*

CCCCAGAACGCCACGCCGCGMGGCGCACACCATTCTGTTGGCCCGCTGGCCAACGGCGCCACCACCATTCTGTTCGAGGGCGTGCCGAACTACCCCGACGTGACCCGCGTGGCGAAGATCATCGACAAGCACAAGGTCAACATCCTCTACACCGCGCCGACCGCGATCCGCGCGATGATGGCCGAAGGCAAGGCGGCGGTGGCCGGTGCCGACGGTTCCAGCCTGCGTCTGCTCGGTTCGGTGGGCGAGCCGATCAACCCGGAAGCCTGGCAGTGGTACTACGAGACCGTCGGCCAGTCGCGCTGCCCGATCGTCGACACCTGGTGGCAGACCGAGACCGGCGCCTGCCTGATGACCCCGCTGCCGGGCGCCCACGCGATGAAGCCGGGCTCCGCGGCCAAGCCGTTCTTCGGCGTGGTTCCGGCACTGCACCGCCGAGGTGGAAAGCGCGATGGTCGCCCACCCGAAGGTCGCCGAGGCGGCGGTGGTCGGCATGCAGCACGACATCAAGGGGCAGGG

*aro allel 11*

GGGGCTTATTGCTGGAAACCCGAGCCGGGCAGCGAGGAAGCGCGCCTGACCGAGTATTTCCTCGCCGACCGCGATTGGCTCGCCGGCCAGCCCTGACCCGCCCGCAGGGGACGAGTCCGCTCGTCCCGTCCCACCCCGGCAAAGGAACGACCCATGGACCGCTATTGCGTATTCGGCAACCCCATCGGCCACAGCAAGTCGCCGCTGATCCACCGCCTGTTCGCCGAGCAGACCGGCGAGGCGCTGGTCTATGACGCGCAGCNGGCGCCGCTGGACGATTTCCCCGGGTTCGCCCGGCGCTTCTTCGAGCAGGGCAAGGGCGCCAATGTCACCGTGCCGTTCAAGGAAGAGGCCTATCGTCTGGTGGACGAATTGAGCGAGCGGGCCACCCGGGCCGGGGCGGTGAACACCCTGATCCGCCTGGCCGACGGTCGCCTGCGCGGCGACAACACCGACGGCGCGGGCTTGCTGCGGGACCTGACGGCGAACGCCGGGGTCGAGCTGCGCGGCAAGCGGGTTCTCCTGCTCGGCGCCGGCGGTGCGGTGCGCGGGGTGCTCGAACCCTTCCTCGGCGAGTGCCCGGCGGAGTTGCTGATCGCCAACCGCACGGCGCGGAAGGCCGTGGATCTGGCCGAGCGGTTCGCCGATCTCGGCGCGGTGCGCGGCTGCGGTTTCGCCGAGGTCGAAGGGCCTTTCGACCTGGTCGTCAACGGCACCTCGGCCAGTCTTGCCGGCGACGTGCCGCCGCTGGCGCAGAGCGTGATCGAGCCCGGCCGTACCGTCTGCTACGACATGATGTATGCCAAGGAACCGACCGCCTTCAACCGCTGGGCCGCCGAACGCGGTGCGGCGCGTACCCTGGATGGCCTGGGCATGCTGGTGGAGCAGGCCGCCGAGGCATTCTTCCTCTGGCGTGGCGTGCGTCCTGCCTCGGCGCCGGTGTTGGAGACGCTGCGCCGGCAATTGGCAACTGTCTGAGTTGTCAGGCAGCATTTGAGACGTACGTCTCAAATCGGTTTCCATCTGACATGTAGGAATCACAAAACCCGGG

*gua allel 3*

TGTGGATGAGCCACGGCGACAAGGTCACCGAGATGCCGGCCGGCTTCCACATCCTGGCCAGCACCCCGAGCTGCCCGATCGCCGCCATGGCCGACGATGCCCGCGCCTACTACGGCGTGCAATTCCACCCGGAAGTCACCCACACCAAGCAGGGCCTGCGCATTCTCTCGCGCTTCGTCCTCGACATCTGTGGTTGCGCCGCGCTGTGGACCCCGTCGAACATCGTCGACGACGCCATCGCCACCGTGCGCGCCCAAGTCGGTTCCTCCAAGGTCCTGCTCGGCCTCTCCGGCGGCGTGGACTCCTCGGTGGTCGCCGCGCTGCTGCACAAGGCCATCGGCGACCAACTGACCTGCGTGTTCGTCGACAACGGCCTGCTGCGCCTGCACGAAGGCGACCAGGTGATGGCCATGTTCGCCGAGAACATGGGCGTGAAGGTGATCCGTGCCAACGCCGAGGACAAGTTCCTCGGCCGCCTGGCCGGCGTCGCCGATCCGGAAGAGAAGCGCAAGATCATCGGCCGCACCTTCATCGAAGTCTTCGACGAAGAAGCCACCAAGCTGCAGGACGTGAAGTTCCTCGCCCAGGGCACCATCTACCCCGACGTGATCGAGTCGGCCGGCGCCAAGACCGGCAAGGCCCACGTGATCAAGTCGCACCACAACGTCGGCGGCCTGCCGGAGGACATGCAGTTCGAACTGGTCGAGCCGCTGCGCGAACTGTTCAAGGACGAAGTGCGCAAGATCGGCCTGGAGCTGGGCCTGCCCTACGACATGGTCTACCGCCACCCGTTCCCCGGCCCGGGCCTGGGCGTGCGCATCCTCGGCGAGGTGAAGAAGGAGTACGCCGACCTGCTGCGCCAGGCCGACCACATCTTCATCGAAGAACTGCGCGCCTTCGACTGGTACCACAAGACCAGCCAGGGCG

*mut allel 13*

AAAACAGCCTTGACGCCGGTTCCCGGCGCATCGACGTGGAGGTCGAGCAGGGCGGCATCAAGTTGCTGCGAGTGCGCGACGACGGTCGCGGCATCCCCGCCGACGACCTGCCGCTGGCCCTGGCTCGCCACGCCACCAGCAAGATCCGCGAGCTGGAAGACCTGGAGCGGGTGATGAGCCTCGGCTTCCGTGGCGAGGCGCTGGCCTCGATCAGCTCGGTAGCGCGCCTGACCATGACCTCGCGTACCGCCGACGCCGGCGAAGCCTGGCAGGTGGAAACCGAGGGCCGCGACATGCAGCCGCGGGTACAGCCGGCGGCGCACCCGGTGGGGACCAGCGTCGAGGTTCGCGACCTGTTCTTCAACACCCCGGCGCGGCGCAAGTTCCTGCGCGCCGAGAAGACCGAGTTCGACCATCTGCAGGAAGTCATCAAGCGCCTGGCGCTGGCCCGTTTCGACGTGGCTTTCCACCTGCGCCACAACGGCAAGACCATCTTCGCCCTGCACGAGGCGCGAGACGAGCTGGCCCGCGCGCGCCGGGTCGGCGCGGTGTGCGGCCAGGCATTCCTCGAGCAGGCGCTGCCGATCGAGGTCGAGCGCAACGGTCTGCACCTGTGGGGCTGGGTCGGCTTGCCGACCTTCTCCCGCAGCCAGCCGGACCTGCAGTACTTCTATGTGAACGGGCGCATGGTGCGCGACAAGCTGGTCGCCCACGCGGTGCGCCAGGCTTATCGCGACGTGCTGTACAACGGCCGGCATCCGACCTTCGTGCTGTTCTTCGAAGTCGATCCGGCGGTGGTGGACGTCAACGTGCACCCGACCAAGCACGAAGTTCGCTTCCGTGACAGCCGGATGGTCCATGACTTCCTCTATGGCACCC

*nuo allel 1*

CCGCCACCCGTACTGGCCCAATGCCAATTGGTACGAGCGCGAAGTGTGGGACATGTACGGCATCACCTTCACCGGCCACCCGCACCTGACCCGCATGCTGATGCCGCCGACCTGGCAGGGCCATCCGCTGCGCAAGGACTACCCGGCGCGCGCCACCGAGTTCGATCCCTACTCCCTGTCCGCCGCCAAGCAGGACCTGGAGCAGGAGGCCCTGCGCTTCAAGCCGGAAGACTGGGGCATGAAGCGCCACGGCGAGAACGAGGACTACATGTTCCTCAACCTCGGCCCGAACCACCCGTCCGCCCACGGCGCGTTCCGCATCATCCTGCAACTGGACGGCGAGGAGATCATCGACTGCGTCCCGGAGATCGGCTACCACCACCGCGGCGCCGAGAAGATGGCCGAGCGCCAGTCCTGGCACAGTTTCATCCCCTACACCGACCGCATCGACTACCTCGGCGGGGTGATGAACAACCTGCCCTACGTACTCTCGGTGGAGAAGCTCGCCGGGATCAAGGTGCCGCAGCGGGTCGACGTGATCCGGATCATGATGGCGGAGTTCTTCCGTATCCTGAACCACCTGCTGTACCTGGGCACCTATATCCAGGACGTCGGCGCCATGACCCCGGTGTTCTTCACCTTCACCGACCGCCAGCGCGCCTACAAGGTGGTCGAGGCCATCACCGGCTTCCGCCTGCACCCGGCCTGGTACCGCATCGGCGGCGTCGCCCACGACCTGCCGCGCGGCTGGGACAAGCTGGTCCGCGAGTTCCTCGACTGGATGCCCAAGCGCCTCGACGAGTACGAGACCGCGGCCCTGAAGAACAGCATCCTGCGCGGCCGGACCATCGGCGTCGCCCAGTACAACACCAAGGAAGCCCT

*pps allel 2*

GCAAGGTAGTGGACGTCGATCGCGCCGATCGCGCCCGCTTCGCCCTGAGCGACGCCGAGGTCACCGAGCTGGCCAAGCAGGCCATGATCATCGAGAAGCACTATGGCCGCCCGATGGACATCGAATGGGCCAAGGACGGTGACGACGGCAAGCTGTACATCGTCCAGGCACGCCCGGAAACCGTGAAGAGCCGCGCCAGCGCCACGGTCATGGAGCGCTACCTGCTGAAAGAGAAGGGGACCGTCCTGGTGGAAGGACGTGCCATCGGCCAGCGCATCGGTGCCGGTCCAGTCAAGGTGATCAACGACGTGTCGGAAATGGACAAGGTCCAACCGGGTGACGTCCTGGTCTCCGACATGACCGACCCGGACTGGGAGCCGGTGATGAAGCGCGCCAGCGCCATCGTCACCAACCGCGGCGGGCGTACCTGCCACGCGGCGATCATCGCTCGCGAACTGGGCATCCCGGCGGTGGTCGGTTGCGGCAACGCCACCCAGATCCTGCAGGATGGGCAGGGGGTGACCGTTTCCTGTGCCGAAGGCGATACCGGCTTCATCTTCGAAGGCGAACTCGGTTTCGATGTGCGCAAGAACTCGGTCGACGCCATGCCCGACCTTCCGTTCAAGATCATGATGAACGTCGGCAATCCCGATCGCGCTTTCGATTTCGCCCAGTTGCCGAACGAAGGCGTGGGCCTGGCCCGCCTCGAATTCATCATCAACCGCATGATCGGCGTGCACCCCAAGGCGTTGCTGAACTTCGCCGGCCTGCCGGCGGACATCAAGGAAAGCGTGGAGAAGCGCATCGCCGGCTATCCCGATCCGGTCGGCTTCTACGTCGAGAAGCTGGTGGAGGGCATCAGCACCCTGGCCGCGGCGTTCTGGCCGAAGAAGGTCATCGTGCGCCTGT

*trp allel 4*

GGTCGTGAGCCGGCGTTTCGTGCCAGCTTCACCCGCGAGGACTATGAAAACGCGGTAGGAAGGATCAAGGACTACATCCTGGCCGGCGACTGCATGCAGGTGGTGCCGTCGCAGCGCATGTCCATCGAGTTCAAGGCGGCGCCCATCGACCTGTACCGCGCGCTGCGCTGTTTCAATCCGACGCCCTACATGTACTTCTTCAACTTCGGCGACTTCCATGTCGTGGGCAGCTCGCCGGAGGTGCTGGTACGGGTCGAGGATGGCCTGGTCACGGTGCGCCCGATCGCCGGTACCCGTCCGCGCGGGATCAACGAAGAGGCCGACCTGGCGCTGGAGCAGGATCTGCTGTCGGACGCCAAGGAGATCGCCGAGCACCTGATGCTGATCGACCTGGGGCGCAACGACGTGGGGCGGGTGTCCGATATCGGCGCGGTGAAGGTCACCGAAAAAATGGTGATCGAACGTTACTCCAACGTCATGCACATCGTGTCCAACGTCACCGGGCAATTGCGCGAGGGGCTCAGCGCGATGGACGCGCTGCGGGCGATCCTGCCGGCGGGTACGCTGTCCGGCGCGCCGAAGATCCGCGCCATGGAGATCATCGACGAGCTGGAGCCGGTCAAGCGTGGAGTCTACGGCGGCGCGGTCGGCTACCTGGCATGGAACGGCAACATGGACACCGCCATTGCCATCCGCACCGCGGTGATCAAGAACGGTGAACTCCACGTGCAGGCCGGCGGCGGTATCGTTGCCGACTCGGTGCCGGCGCTGGAGTGGGAAGAAACCATC

**Strain no. 408 ST 244**

*acs allel 17*

TCGCCTCGCTGACCCACGAGCGGGTYTTCGACTACCGTCCGGGCGAAGTCTACTGGTGCACCGCCGACATCGGCTGGGTCACCGGCCACACCTACATCGTCTATGGCCCGCTGGCCAACGGCGCCACCACCATTCTGTTCGAGGGCGTGCCGAACTACCCCGACGTGACCCGCGTGGCGAAAATCATCGACAAGCACAAGGTCAACATCCTCTACACCGCGCCGACCGCGATCCGCGCGATGATGGCCGAAGGCAAGGCGGCGGTGGCCGGTGCCGACGGTTCCAGCCTGCGTCTGCTCGGTTCGGTGGGCGAGCCGATCAACCCGGAAGCCTGGCAGTGGTACTACGAGACCGTCGGCCAGTCGCGCTGCCCGATCGTCGACACCTGGTGGCAGACCGAGACCGGCGCCTGCCTGATGACCCCGCTGCCGGGCGCCCACGCGATGAAGCCGGGCTCTGCAGCCAAGCCGTTCTTCGGCGTGGTACCGGCACTGGTGGACAATCTCGGCAACCTGATCGAGGGCGCCGCCGAGGGCAACCTGGTGATCCTCGACTCCTGGCCGGGCCAGGCGCGGACCCTGTTCGGCGACCATGACCGCTTCGTCGACACCTACTTCAAGACCTTCAAGGGCATGTACTTCACCGGCGACGGCGCGCGCCGCGACGAGGACGGCTACTACTGGATCACCGGGCGGGTCGACGACGTGCTCAACGTCTCCGGCCACCGCATGGGCACCGCCGAGGTGGAAAGCGCGATGGTCGCCCACCCGAAGGTCGCCGAGGCGGCGGTGGTCGGCATGCAGCACGACATCAAGGGGCAGGGA

*aro allel 5*

GATGGAACCCGAGCCGGGCAGCGAGGAAGCGCGCCTGACCGAGTATTTCCTCGCCGACCGCGATTGGCTCGCCGGCCAGCCCTGACCCGCCCGCAGGGGACGAGTCCGCTCGTCCCGTCCCACTCCGGCAAAGGAACGACCCATGGACCGCTATTGCGTATTCGGCAACCCCATCGGCCACAGCAAGTCGCCGCTGATCCACCGCCTGTTCGCCGAGCAGACCGGCGAGGCGCTGGTCTATGACGCGCAACTGGCGCCGCTGGACGATTTCCCCGGGTTCGCCCGGCGCTTCTTCGAGCAGGGCAAGGGCGCCAATGTCACCGTGCCGTTCAAGGAAGAGGCCTATCGTCTGGTGGACGAGTTGAGCGAGCGGGCCACCCGGGCCGGGGCGGTGAACACCCTGATCCGCCTCGCCGACGGTCGCCTGCGCGGCGACAACACCGACGGCGCCGGCCTGCTGCGGGACCTGACGGCGAACGCCGGGGTCGAGCTGCGCGGCAAGCGGGTTCTCCTGCTCGGCGCCGGCGGTGCGGTGCGTGGGGTGCTCGAACCCTTCCTCGGCGAGTGCCCGGCGGAGTTGCTGATCGCCAACCGCACGGCGCGGAAGGCCGTGGACCTGGCCGAGCGGTTCGCCGACCTCGGCGCGGTGCACGGCTGCGGTTTCGCCGAGGTCGAAGGGCCTTTCGACCTGATCGTCAACGGCACCTCGGCCAGTCTTGCCGGCGACGTGCCGCCGCTGGCGCAGAGCGTGATCGAGCCCGGCCGTACCGTCTGCTACGACATGATGTATGCCAAGGAACCGACTGCCTTCAACCGCTGGGCCGCCGAACGCGGTGCGGCGCGTACCCTGGATGGCCTGGGC

*gua allel 12*

CGGTGGATGAGCCACGGCGACAAGGTCACCGAGATGCCGGCCGGCTTCCACATCCTGGCCAGCACCCCGAGCTGCCCGATCGCCGCCATGGCCGACGATGCCCGCGCCTACTACGGCGTGCAATTCCACCCGGAAGTCACCCACACCAAGCAGGGCCTGCGCATTCTCTCGCGCTTCGTCCTCGACATCTGCGGTTGCGCCGCGCTGTGGACCCCCTCGAACATCGTCGACGACGCCATCGCCACCGTGCGCGCCCAGGTCGGTTCCTCCAAGGTCCTGCTCGGCCTCTCCGGCGGCGTGGACTCCTCGGTGGTCGCCGCGCTGCTGCACAAGGCCATCGGCGACCAACTGACCTGCGTGTTCGTCGACAACGGCCTGCTGCGCCTGCACGAAGGCGACCAGGTGATGGCCATGTTCGCCGAGAACATGGGCGTGAAGGTGATCCGCGCCAACGCCGAGGACAAGTTCCTCGGCCGCCTGGCCGGCGTCGCCGACCCGGAAGAGAAGCGCAAGATCATCGGCCGCACCTTCATCGAAGTCTTCGACGAAGAAGCCACCAAGCTGCAGGACGTGAAGTTCCTCGCCCAGGGCACCATCTACCCCGACGTGATCGAGTCGGCCGGCGCCAAGACCGGCAAGGCCCACGTGATCAAGTCGCACCACAACGTCGGCGGCCTGCCGGAGGACATGCAGTTCGAACTGGTCGAGCCGCTGCGCGAACTGTTCAAGGACGAAGTGCGCAAGATCGGCCTGGAGCTGGGCCTGCCCTACGACATGGTCTACCGCCACCCGTTCCCCGGCCCGGGCCTGGGCGTGCGCATCCTCGGCGAGGTGAAGAAGGAGTACGCCGACCTGCTGCGCCAGGCCGACCACATCTTCATCGAGGAACTGCGCGCCTTCGACTGGTACCACAAGACCAGCCARGGCGTTCA

*mut allel 3*

TTGACGCCGGTTCCCGGCGCATTGATGTGGAGGTCGAGCAGGGCGGCATCAAGTTGCTGCGAGTGCGCGACGACGGTCGCGGCATCCCCGCCGACGACCTGCCGCTGGCCCTGGCTCGCCACGCCACCAGCAAGATCCGCGAGCTGGAAGACCTGGAGCGGGTGATGAGCCTCGGCTTCCGTGGCGAGGCGCTGGCCTCGATCAGCTCGGTAGCGCGCCTGACCATGACCTCGCGTACCGCCGACGCCGGCGAAGCCTGGCAGGTGGAAACCGAGGGCCGCGACATGCAGCCGCGGGTACAGCCGGCGGCGCACCCGGTGGGGACCAGCGTCGAGGTTCGCGACCTGTTCTTCAACACCCCGGCGCGGCGCAAGTTCCTGCGTGCCGAGAAGACCGAGTTCGACCATCTGCAGGAGGTCATCAAGCGCCTGGCGCTGGCCCGCTTCGACGTGGCTTTCCACCTGCGCCACAACGGCAAGACCATCTTCGCCCTGCACGAGGCGCGAGACGAGCTGGCCCGCGCGCGCCGGGTCGGCGCGGTGTGCGGCCAGGCATTCCTCGAGCAGGCGCTGCCGATCGAGGTCGAGCGCAACGGCCTGCACCTGTGGGGCTGGGTCGGCTTGCCGACCTTCTCCCGCAGCCAGCCGGACCTGCAGTACTTCTATGTGAACGGGCGCATGGTGCGCGACAAGCTGGTCGCCCACGCGGTGCGCCAGGCTTATCGCGACGTGCTGTACAACGGCCGGCATCCGACCTTCGTGCTGTTCTTCGAAGTCGATCCGGCGGTGGTGGACGTCAACGTGCACCCGACCAAGCACGAAGTTCGCTTCCGTGACAGCCGGATGGTCCATGACTTCCTCTATGGCACCC

*nuo allel 14*

CCGCCACCCGTACTGGCCCAACGCCAACTGGTACGAGCGCGAAGTGTGGGACATGTACGGCATCACCTTCACCGGCCACCCGCACCTGACCCGCATGCTGATGCCGCCGACCTGGCAGGGCCATCCGCTGCGCAAGGATTACCCGGCGCGCGCCACCGAGTTCGATCCCTACTCCCTGTCCGCCGCCAAGCAGGACCTGGAGCAGGAAGCCCTGCGCTTCAAGCCGGAAGACTGGGGCATGAAGCGCCACGGCGAGAACGAGGACTACATGTTCCTCAACCTCGGCCCGAACCACCCGTCCGCCCACGGCGCGTTCCGCATCATCCTGCAACTGGACGGCGAGGAGATCATCGACTGCGTCCCGGAGATCGGCTACCACCACCGCGGCGCCGAGAAGATGGCCGAGCGCCAGTCCTGGCACAGTTTCATCCCCTACACCGACCGCATCGACTACCTCGGCGGGGTGATGAACAACCTGCCCTACGTACTCTCGGTGGAGAAGCTCGCAGGGATCAAGGTGCCGCAGCGGGTCGACGTGATCCGGATCATGATGGCGGAGTTCTTCCGTATCCTGAACCACCTGCTGTACCTGGGCACCTATATCCAAGACGTCGGCGCCATGACCCCGGTGTTCTTCACCTTCACCGACCGCCAGCGCGCCTACAAGGTGGTCGAGGCCATCACCGGCTTCCGCCTGCACCCGGCCTGGTACCGCATCGGTGGCGTCGCCCACGACCTGCCGCGCGGCTGGGACAAGCTGGTCCGCGAGTTCCTCGACTGGATGCCCAAGCGCCTCGACGAGTACGAGACCGCGGCCCTGAAGAACAGCATCCTGCGCGGCCGGACCATC

*pps allel 4*

CAAGGTAGTGGACGTCGATCGCGCCGATCGCGCCCGCTTCGCCCTGAGCGACGCCGAGGTCACCGAGCTGGCCAAGCAGGCCATGATCATCGAGAAGCACTATGGCCGCCCGATGGACATCGAATGGGCCAAGGACGGTGACGACGGCAAGCTGTACATCGTCCAGGCACGCCCGGAAACCGTGAAGAGCCGCGCCAGCGCCACGGTCATGGAGCGCTACCTGCTGAAAGAGAAGGGGACCGTCCTGGTGGAAGGGCGTGCCATCGGCCAGCGCATCGGTGCCGGTCCGGTCAAGGTGATCAACGACGTGTCGGAAATGGACAAGGTCCAACCGGGTGACGTCCTGGTCTCCGACATGACCGACCCGGACTGGGAGCCGGTGATGAAGCGCGCCAGCGCCATCGTCACCAACCGCGGCGGGCGTACCTGCCACGCGGCGATCATCGCTCGCGAACTGGGCATCCCGGCGGTGGTCGGTTGCGGCAACGCCACCCAGATCCTGCAGGATGGCCAGGGGGTGACCGTTTCCTGTGCCGAAGGCGATACCGGCTTCATCTTCGAAGGCGAACTCGGTTTCGATGTGCGCAAGAACTCGGTCGACGCCATGCCCGACCTTCCGTTCAAGATCATGATGAACGTCGGCAATCCCGATCGCGCTTTCGATTTCGCCCAGTTGCCGAACGAAGGCGTGGGCCTGGCCCGCCTCGAATTCATCATCAACCGCATGATCGGCGTGCACCCCAAGGCATTGCTGAACTTCGCCGGCCTGCCGGCGGACATCAAGGAAAGCGTGGAGAAGCGCATCGCCGGCTATCCCGATCCGGTCGGCTTCTACGTCGAGAAGCTGGTGGAAGGCATCAGCACCCTGGCCGCGGCGTTCTGGCCGAAGAAGGTCATCGTGCGCCTGTCCGACTTCAAGTCCAATGAGTACGCCAACCTGATCGGCGGCAAGCTCTACGAGCCGG

*trp allel 7*

GGTCGTGAGCCGGCGTTTCGTGCCAGCTTCACCCGCGAGGACTATGAAAACGCGGTAGGAAGGATCAAGGACTACATCCTGGCCGGCGACTGCATGCAGGTGGTGCCGTCGCAGCGCATGTCCATCGAATTCAAGGCGGCGCCCATCGACCTGTACCGCGCGCTGCGCTGTTTCAATCCGACGCCCTACATGTACTTCTTCAACTTCGGCGACTTCCATGTCGTGGGCAGCTCGCCGGAGGTGCTGGTACGGGTCGAGGATGGCCTGGTGACGGTGCGCCCGATCGCCGGTACCCGTCCGCGCGGGATCAACGAAGAGGCCGACCTGGCACTGGAGCAGGATCTGCTGTCGGACGCCAAGGAGATCGCCGAGCACCTGATGCTGATCGACCTGGGGCGCAACGACGTGGGGCGGGTGTCCGATATCGGCGCGGTGAAGGTCACCGAAAAAATGGTGATCGAACGTTACTCCAACGTCATGCACATCGTGTCCAACGTCACCGGGCAATTGCGCGAGGGGCTCAGCGCGATGGACGCGCTGCGGGCGATTCTGCCGGCGGGCACTCTATCCGGCGCGCCGAAGATCCGCGCCATGGAGATCATCGACGAGCTGGAGCCGGTCAAGCGTGGAGTCTACGGCGGCGCGGTCGGCTACCTGGCATGGAACGGCAACATGGACACCGCCATTGCCATCCGCACCGCGGTGATCAAGAACGGTGAACTCCACGTGCAGGCCGGCGGCGGTATCGTTGCCGACTCGGTGCCCGCGCTGGAGTGGGAAGAAACCATCAACAAG

**Strain no. 429 ST396**

*acs allel 114*

TCAAGCGTMGGCGCACCACCATTCTGTTCGAGGGCGTGCCGAACTACCCCGACGTGACCCGCGTGGCGAAGATCATCGACAAGCACAAGGTCAACATCCTCTACACCGCGCCGACCGCGATCCGCGCGATGATGGCCGAAGGCAAGGCGGCGGTGGCCGGTGCCGACGGTTCCAGCCTGCGTCTGCTCGGTTCGGTGGGCGAGCCGATCAACCCGGAAGCCTGGCAGTGGTACTACGAGACCGTCGGCCAGTCGCGCTGCCCGATCGTCGACACCTGGTGGCAGACCGAGACCGGCGCCTGCCTGATGACCCCGCTGCCGGGCGCCCACGCGATGAAGCCGGGCTCTGCAGCCAAGCCGTTCTTCGGCGTGGTCCCGGCACTGGTGGACAACCTCGGCAACCTGATCGAGGGCGCCGCCGAGGGCAACCTGGTGATCCTCGACTCCTGGCCGGGCCAGGCGCGGACCCTGTTCGGCGACCATGACCGCTTCGTCGACACCTACTTCAAGACCTTCAAGGGCATGTACTTCACCGGCGACGGCGCGCGCCGCGACGAGGACGGCTACTACTGGATCACCGGGCGGGTCGACGACGTGCTCAACGTCTCCGGCCACCGCATGGGCACCGCCGAGGTGGAAAGCGCGATGGTCGCCCACCCGAAGGTCGCCGAGGCGGCGGTGGTCGGCATGCAGCACGACATCAAGGGGCAGGGAT

*aro allel 73*

CGGGCTCGATCACGCTCTGCGCCAGCGGCGGCACGTCGCCGGCAAGACTAGCCGAGGTGCCGTTGACGATCAGGTCGAAAGGCCCTTCGACCTCGGCGAAACCGCAGCCGTGCACCGCGCCGAGGTCGGCGAACCGCTCGGCCAGGTCCACGGCCTTCCGCACCGTGCGGTTGGCGATCAGCAACTCCGCCGGGCACTCGCCGAGGAAGGGTTCGAGCACCCCACGCACCGCACCGCCGGCGCCGAGCAGGAGAACCCGCTTGCCGCGCAGCTCGACCCCGGCGTTCGCCGTCAGGTCCCGCAGCAGGCCGGCGCCGTCGGTGTTGTCGCCGCGCAGGCGACCGTCGGCGAGGCGGATCAGGGTGTTCACCGCCCCGGCCCGGGTGGCCCGCTCGCTCAACTCGTCCACCAGACGATAGGCCTCTTCCTTGAACGGCACGGTGACATTGGCGCCCTTGCCCTGCTCGAAGAAGCTCCGGGCGAACCCGGGGAAATCGTCCAGCGGCGCCAGTTGCGCGTCATAGACCAGCGCCTCGCCGGTCTGCTCGGCGAACAGGCGGTGGATCAGCGGCGACTTGCTGTGGCCGATGGGGTTGCCGAATACGCAATAGCGGTCCATGGGTCGTTCCTTTGCCGGAGTGGGACGGGACGAGCGGACTCGTCCCCTGCGGGCGGGTCAGGGCTGGCCGGCGAGCCAATCGCGGTCGGCGAGGAAATACTCGGTCAGGCGCGCTTCCTCGCTGCCCGGCTCGGGTTTCCAGTCATAGCCCC

*gua allel 32*

GGTGGATGAGCCACGGCGACAAGGTCACCGAGATGCCGGCCGGCTTCCACATCCTGGCCAGCACCCCGAGCTGCCCGATCGCCGCCATGGCCGACGATGCCCGCGCCTACTACGGCGTGCAATTCCACCCGGAAGTCACCCACACCAAGCAGGGCCTGCGCATTCTCTCGCGCTTCGTCCTCGACATCTGCGGTTGCGCCGCGCTGTGGACCCCCTCGAACATCGTCGACGACGCCATCGCCACCGTGCGCGCCCAGGTCGGTTCCTCCAAGGTCCTGCTAGGCCTCTCCGGCGGCGTGGACTCCTCGGTGGTCGCCGCGCTGTTGCACAAGGCCATCGGCGACCAACTGACCTGCGTGTTCGTCGACAACGGCCTGCTGCGCCTGCACGAAGGCGACCAGGTGATGGCCATGTTCGCCGAGAACATGGGCGTGAAGGTGATCCGCGCCAACGCCGAGGACAAGTTCCTCGGCCGCCTGGCCGGCGTCGCCGACCCGGAAGAGAAGCGCAAGATCATCGGCCGCACCTTCATCGAAGTTTTCGACGAAGAAGCCACCAAGCTGCAGGACGTGAAGTTCCTCGCCCAGGGCACCATCTACCCCGACGTGATCGAGTCGGCCGGCGCCAAGACCGGCAAGGCCCACGTGATCAAGTCGCACCACAACGTCGGCGGCCTGCCGGAGGACATGCAGTTCGAACTGGTCGAGCCGCTGCGCGAACTGTTCAAGGACGAAGTGCGCAAGATCGGCCTGGAGCTGGGCCTGCCCTACGACATGGTCTACCGCCACCCGTTCCCCGGCCCGGGCCTGGGCGTGCGCATCCTCGGCGAGGTGAAGAAGGAGTACGCCGACCTGCTGCGCCAGGCCGACCACATCTTCATCGAGGAACTGCGCGCCTTCGACTGGTACCACAAGACCAGCCAAGGCG

*mut allel 3*

CCGACGACCTGCCGCTGGCCCTGGCTCGCCACGCCACCAGCAAGATCCGCGAGCTGGAAGACCTGGAGCGGGTGATGAGCCTCGGCTTCCGTGGCGAGGCGCTGGCCTCGATCAGCTCGGTAGCGCGCCTGACCATGACCTCGCGTACCGCCGACGCCGGCGAAGCCTGGCAGGTGGAAACCGAGGGCCGCGACATGCAGCCGCGGGTACAGCCGGCGGCGCACCCGGTGGGGACCAGCGTCGAGGTTCGCGACCTGTTCTTCAACACCCCGGCGCGGCGCAAGTTCCTGCGTGCCGAGAAGACCGAGTTCGACCATCTGCAGGAGGTCATCAAGCGCCTGGCGCTGGCCCGCTTCGACGTGGCTTTCCACCTGCGCCACAACGGCAAGACCATCTTCGCCCTGCACGAGGCGCGAGACGAGCTGGCCCGCGCGCGCCGGGTCGGCGCGGTGTGCGGCCAGGCATTCCTCGAGCAGGCGCTGCCGATCGAGGTCGAGCGCAACGGCCTGCACCTGTGGGGCTGGGTCGGCTTGCCGACCTTCTCCCGCAGCCAGCCGGACCTGCAGTACTTCTATGTGAACGGGCGCATGGTGCGCGACAAGCTGGTCGCCCACGCGGTGCGCCAGGCTTATCGCGACGTGCTGTACAACGGCCGGCATCCGACCTTCGTGCTGTTCTTCGAAGTCGATCCGGCGGTGGTGGACGTCAACGTGCACCCGACCAAGCACGAAGTTCGCTTCCGTGACAGCCGGATGGTCCATGACTTCCTCTATGGCACCC

*nuo allel 4*

CCGCCAACCCGTACTGGCCCAACGCCAACTGGTACGAGCGCGAAGTGTGGGACATGTACGGCATCACCTTCACCGGCCACCCGCACCTGACCCGCATGCTGATGCCGCCGACCTGGCAGGGCCATCCGCTGCGCAAGGACTACCCGGCGCGCGCCACCGAGTTCGATCCCTACTCCCTGTCCGCCGCCAAGCAGGACCTGGAGCAGGAAGCCCTGCGCTTCAAGCCGGAAGACTGGGGCATGAAGCGCCACGGCGAGAACGAGGACTACATGTTCCTCAACCTCGGCCCGAACCACCCGTCCGCCCACGGCGCGTTCCGCATCATCCTGCAACTGGACGGCGAGGAGATCATCGACTGCGTCCCGGAGATCGGCTACCACCACCGCGGCGCCGAGAAGATGGCCGAGCGCCAGTCCTGGCACAGTTTCATCCCCTACACCGACCGCATCGACTACCTCGGCGGGGTGATGAACAACCTGCCCTACGTACTCTCGGTGGAGAAGCTCGCCGGGATCAAGGTGCCCCAGCGGGTCGACGTGATCCGGATCATGATGGCGGAGTTCTTCCGTATCCTGAACCACCTGCTGTACCTGGGCACCTATATCCAGGACGTCGGCGCCATGACCCCGGTGTTCTTCACCTTCACCGACCGCCAGCGCGCCTACAAGGTGGTCGAGGCCATCACCGGCTTCCGCCTGCACCCGGCCTGGTACCGCATCGGCGGCGTCGCCCACGACCTGCCGCGCGGCTGGGACAAGCTGGTCCGCGAGTTCCTCGACTGGATGCCCAAGCGCCTCGACGAGTACGAGACCGCGGCCCTGAAGAACAGCATCC

*pps allel 4*

GTCAAGGTAGTGGACGTCGATCGCGCCGATCGCGCCCGCTTCGCCCTGAGCGACGCCGAGGTCACCGAGCTGGCCAAGCAGGCCATGATCATCGAGAAGCACTATGGCCGCCCGATGGACATCGAATGGGCCAAGGACGGTGACGACGGCAAGCTGTACATCGTCCAGGCACGCCCGGAAACCGTGAAGAGCCGCGCCAGCGCCACGGTCATGGAGCGCTACCTGCTGAAAGAGAAGGGGACCGTCCTGGTGGAAGGGCGTGCCATCGGCCAGCGCATCGGTGCCGGTCCGGTCAAGGTGATCAACGACGTGTCGGAAATGGACAAGGTCCAACCGGGTGACGTCCTGGTCTCCGACATGACCGACCCGGACTGGGAGCCGGTGATGAAGCGCGCCAGCGCCATCGTCACCAACCGCGGCGGGCGTACCTGCCACGCGGCGATCATCGCTCGCGAACTGGGCATCCCGGCGGTGGTCGGTTGCGGCAACGCCACCCAGATCCTGCAGGATGGCCAGGGGGTGACCGTTTCCTGTGCCGAAGGCGATACCGGCTTCATCTTCGAAGGCGAACTCGGTTTCGATGTGCGCAAGAACTCGGTCGACGCCATGCCCGACCTTCCGTTCAAGATCATGATGAACGTCGGCAATCCCGATCGCGCTTTCGATTTCGCCCAGTTGCCGAACGAAGGCGTGGGCCTGGCCCGCCTCGAATTCATCATCAACCGCATGATCGGCGTGCACCCCAAGGCATTGCTGAACTTCGCCGGCCTGCCGGCGGACATCAAGGAAAGCGTGGAGAAGCGCATCGCCGGCTATCCCGATCCGGTCGGCTTCTACGTCGAGAAGCTGGTGGAAGGCATCAGCACCCTGGCCGCGGCGTTCTGGCCGAAGAAGGTCATCGTGCGCCTGTCCGACTTCAAGTCCAATGAGTACGCCAACCTGATCGGCGGCAAGCTCTAC

*trp allel 1*

GGTCGTGAGCCGGCGTTTCGTGCCAGCTTCACCCGCGAGGACTATGAAAACGCGGTAGGAAGGATCAAGGACTACATCCTGGCCGGCGACTGCATGCAGGTGGTGCCGTCGCAGCGCATGTCCATCGAATTCAAGGCGGCGCCCATCGACCTGTACCGCGCGCTGCGCTGTTTCAATCCGACGCCCTACATGTACTTCTTCAACTTCGGCGACTTCCATGTCGTGGGCAGCTCGCCGGAGGTGCTGGTACGGGTCGAGGATGGCCTGGTGACGGTGCGCCCGATCGCCGGTACCCGTCCGCGCGGGATCAACGAAGAGGCCGACCTGGCGCTGGAGCAGGATCTGCTGTCGGACGCCAAGGAGATCGCCGAGCACCTGATGCTGATCGACCTGGGGCGCAACGACGTGGGGCGGGTGTCCGATATCGGCGCGGTGAAGGTCACCGAAAAAATGGTGATCGAACGTTACTCCAACGTCATGCACATCGTGTCCAACGTCACCGGGCAATTGCGCGAGGGGCTCAGCGCGATGGACGCGCTGCGGGCGATTCTGCCGGCGGGCACTCTATCCGGCGCGCCGAAGATCCGCGCCATGGAGATCATCGACGAGCTGGAGCCGGTCAAGCGTGGAGTCTACGGCGGCGCGGTCGGCTACCTGGCATGGAACGGCAACATGGACACCGCCATTGCCATCCGCACCGCGGTGATCAAGAACGGTGAACTCCACGTGCAGGCCGGCGGCGGTATCGTTGCCGACTCGGTGCCCGCGCTGGAGTGGGAAGAAACCATCAACAA

**Strain no. 520 ST348**

*acs allel 22*

GRSCTTGGCCCGCTGGCCAACGGCGCCACCACCATTCTGTTCGAGGGCGTGCCGAACTACCCCGACGTGACCCGCGTGGCGAAAATCATCGACAAGCACAAGGTCAACATCCTCTACACCGCGCCGACCGCGATCCGCGCGATGATGGCCGAAGGCAAGGCGGCGGTGGCCGGTGCCGACGGTTCCAGCCTGCGTCTGCTCGGTTCGGTGGGCGAGCCGATCAACCCGGAAGCCTGGCAGTGGTACTACGAGACCGTCGGCCAGTCGCGCTGCCCGATCGTCGACACCTGGTGGCAGACCGAGACCGGCGCCTGCCTGATGACCCCGCTGCCGGGCGCCCACGCGATGAAGCCGGGCTCCGCGGCCAAGCCGTTCTTCGGCGTGGTACCGGCACTGACCAGWWAA

*aro allel 20*

GGGGCTAATGATGGAAACCCGAGCCGGGCAGCGAGGAAGCGCGCCTGACCGAGTATTTCCTCGCCGACCGCGATTGGCTCGCCGGCCAGCCCTGACCCGCCCGCAGGGGACGAGTCCGCTCGTCCCGTCCCACTCCGGCAAAGGAACGACCCATGGACCGCTATTGCGTATTCGGCAACCCCATCGGCCACAGCAAGTCGCCGCTGATCCACCGCCTGTTCGCCGAGCAGACCGGCGAGGCGCTGGTCTATGACGCGCAACTGGCGCCGCTGGACGATTTCCCCGGGTTCGCCCGGCGCTTCTTCGAGCAGGGCAAGGGCGCCAATGTCACCGTGCCGTTCAAGGAAGAGGCCTATCGCCTGGTGGACGAGTTGAGCGAGCGGGCCACCCGGGCCGGGGCGGTGAACACCCTGATCCGCCTCGCCGACGGTCGCCTGCGCGGCGACAACACCGACGGCGCCGGCCTGCTGCGGGACCTGACGGCGAACGCCGGGGTCGAGCTGCGCGGCAAGCGGGTTCTCCTGCTCGGCGCCGGCGGTGCGGTGCGTGGGGTGCTCGAACCCTTCCTCGGCGAGTGCCCGGCGGAGTTGCTGATCGCCAACCGCACGGCGCGGAAGGCCGTGGACCTGGCCGAGCGGTTCGCCGACCTCGGCGCGGTGCACGGCTGCGGTTTCGCCGAGGTCGAAGGGCCTTTCGACCTGATCGTCAACGGCACCTCGGCCAGTCTTGCCGGCGACGTGCCGCCGCTGGCGCAGAGCGTGATCGAGCCCGGCCGTACCGTCTGCTACGACATGATGTATGCCAAGGAACCGACTGCCTTCAACCGCTGGGCCGCCGAACGCGGTGCGGCGCGTACCCTGGATGGCCTGGGCATGCTGGTGGAGCAGGCCGCCGAGGCATTCTTCCTCTGGCGCGGCGTGCGTCCTGCCTCGGCGCCAGTGTTGGAGACGCTGCGCCGACAGTTGGCAACTGTCTGAGTTGTCAGGCGGCATTTGAGACGTACGTCTCAAATTGGCTTCCATCTGACATGTAGGAATCACAAACCCGGG

*gua allel 11*

GTGGATGAGCCACGGCGACAAGGTCACCGAGATGCCGGCCGGCTTCCACATCCTGGCCAGCACCCCGAGCTGCCCGATCGCCGCCATGGCCGACGATGCCCGCGCCTACTACGGCGTGCAATTCCACCCGGAAGTCACCCACACCAAGCAGGGCCTGCGCATTCTCTCGCGCTTCGTCCTCGACATCTGCGGTTGCGCCGCGCTGTGGACCCCCTCGAACATCGTCGACGACGCCATCGCCACCGTGCGCGCCCAGGTCGGTTCCTCCAAGGTCCTGCTAGGCCTCTCCGGCGGCGTGGACTCCTCGGTGGTCGCCGCGCTGCTGCACAAGGCCATCGGCGACCAACTGACCTGCGTGTTCGTCGACAACGGCCTGCTGCGCCTGCACGAAGGCGACCAGGTGATGGCCATGTTCGCCGAGAACATGGGCGTGAAGGTGATCCGCGCCAACGCCGAGGACAAGTTCCTCGGCCGCCTGGCCGGCGTCGCCGACCCGGAAGAGAAGCGCAAGATCATCGGCCGCACCTTCATCGAAGTTTTCGACGAAGAAGCCACCAAGCTGCAGGACGTGAAGTTCCTCGCCCAGGGCACCATCTACCCCGACGTGATCGAGTCGGCCGGCGCCAAGACCGGCAAGGCCCACGTGATCAAGTCGCACCACAACGTCGGCGGCCTGCCGGAGGACATGCAGTTCGAACTGGTCGAGCCGCTGCGCGAACTGTTCAAGGACGAAGTGCGCAAGATCGGCCTGGAGCTGGGCCTGCCCTACGACATGGTCTACCGCCACCCGTTCCCCGGCCCGGGCCTGGGCGTGCGCATCCTCGGCGAGGTGAAGAAGGAGTACGCCGACCTGCTGCGCCAGGCCGACCACATCTTCATCGAGGAACTGCGCGCCTTCGACTGGTACCACAAGACCAGCC

*mut allel 3*

CACGCCACCAGCAAGATCCGCGAGCTGGAAGACCTGGAGCGGGTGATGAGCCTCGGCTTCCGTGGCGAGGCGCTGGCCTCGATCAGCTCGGTAGCGCGCCTGACCATGACCTCGCGTACCGCCGACGCCGGCGAAGCCTGGCAGGTGGAAACCGAGGGCCGCGACATGCAGCCGCGGGTACAGCCGGCGGCGCACCCGGTGGGGACCAGCGTCGAGGTTCGCGACCTGTTCTTCAACACCCCGGCGCGGCGCAAGTTCCTGCGTGCCGAGAAGACCGAGTTCGACCATCTGCAGGAGGTCATCAAGCGCCTGGCGCTGGCCCGCTTCGACGTGGCTTTCCACCTGCGCCACAACGGCAAGACCATCTTCGCCCTGCACGAGGCGCGAGACGAGCTGGCCCGCGCGCGCCGGGTCGGCGCGGTGTGCGGCCAGGCATTCCTCGAGCAGGCGCTGCCGATCGAGGTCGAGCGCAACGGCCTGCACCTGTGGGGCTGGGTCGGCTTGCCGACCTTCTCCCGCAGCCAGCCGGACCTGCAGTACTTCTATGTGAACGGGCGCATGGTGCGCGACAAGCTGGTCGCCCACGCGGTGCGCCAGGCTTATCGCGACGTGCTGTACAACGGCCGGCATCCGACCTTCGTGCTGTTCTTCGAAGTCGATCCGGCGGTGGTGGACGTCAACGTGCACCCGACCAAGCACGAAGTTCGCTTCCGTGACAGCCGGATGGTCCATGACTTCCTCTATGGCACCC

*nuo allel 3*

GACTACCCGGCGCGCGCCACCGAGTTCGATCCCTACTCCCTGTCCGCCGCCAAGCAGGACCTGGAGCAGGAGGCCCTGCGCTTCAAGCCGGAAGACTGGGGCATGAAGCGCCACGGCGAGAACGAGGACTACATGTTCCTCAACCATGTTCCTCAACCTCGGCCCGAACCACCCGTCCGCCCACGGCGCGTTCCGCATCATCCTGCAGCTGGACGGCGAGGAGATCATCGACTGCGTCCCGGAGATCGGCTACCACCACCGCGGCGCCGAGAAGATGGCCGAGCGCCAGTCCTGGCACAGTTTCATTCCCTACACCGACCGCATCGACTACCTCGGCGGGGTGATGAACAACCTGCCCTACGTACTCTCGGTGGAGAAGCTCGCCGGGATCAAGGTGCCGCAGCGGGTCGACGTGATCCGGATCATGATGGCGGAGTTCTTCCGTATCCTGAACCACCTGCTGTACCTGGGCACCTATATCCAGGACGTCGGCGCCATGACCCCGGTGTTCCCTACAAGGTGGTCGAGGCCATCACCGGCTTCCGCCTGCACCCGGCCTGGTACCGCATCGGCGGCGTCGCCCACGACCTGCCGCGCGGCTGGGACAAGCTGGTCCGCGAGTTCCTCGACTGGATGCCCAAGCGCCTCGACGAGTACGAGACCGCGGCCCTGAAGAACAGCATCTTGCGCGGCCGGACCATCGGCGTCGCCCAGTACAACACCAAGGAAGCCC

*pps allel 3*

CAAGGTAGTGGACGTCGATCGCGCCGATCGCGCCCGCTTCGCCCTGAGCGACGCCGAGGTCACCGAGCTGGCCAAGCAGGCCATGATCATCGAGAAGCACTATGGCCGCCCGATGGACATCGAATGGGCCAAGGACGGTGACGACGGCAAGCTGTACATCGTCCAGGCACGCCCGGAAACCGTGAAGAGCCGCGCCAGCGCCACGGTCATGGAGCGCTACCTGCTGAAAGAGAAGGGGACCGTCCTGGTGGAAGGGCGTGCCATCGGCCAGCGCATCGGTGCCGGTCCTGTCAAGGTGATCAACGACGTGTCGGAAATGGACAAGGTCCAACCGGGTGACGTCCTGGTCTCCGACATGACCGACCCGGACTGGGAGCCGGTGATGAAGCGCGCCAGCGCCATCGTCACCAACCGCGGCGGGCGTACCTGCCACGCGGCGATCATCGCTCGCGAACTGGGCATCCCGGCGGTGGTCGGTTGCGGCAACGCCACCCAGATCCTGCAGGATGGCCAGGGGGTGACCGTTTCCTGTGCCGAAGGCGATACCGGCTTCATCTTCGAAGGCGAACTCGGTTTCGATGTGCGCAAGAACTCGGTCGACGCCATGCCCGACCTTCCGTTCAAGATCATGATGAACGTCGGCAATCCCGATCGCGCTTTCGATTTCGCCCAGTTGCCGAACGAAGGCGTGGGCCTGGCCCGCCTCGAATTCATCATCAACCGCATGATCGGCGTGCACCCCAAGGCATTGCTGAACTTCGCCGGCCTGCCGGCGGACATCAAGGAAAGCGTGGAGAAGCGCATCGCGGGCTATCCCGATCCGGTCGGCTTCTACGTCGAGAAACTGGTGGAGGGCATCAGCACCCTGGCCGCGGCGTTCTGGCCGAAGAAGGTCATCGTGCGCCTGTCCGACTTCAAGTCCAATGAGTACGCCAACCTGATCGGCGGCAAGCTCTACGAGCCGG

*trp allel 7*

GGCCCAAGGGTCGTGAGCCGGCGTTTCGTGCCAGCTTCACCCGCGAGGACTATGAAAACGCGGTAGGAAGGATCAAGGACTACATCCTGGCCGGCGACTGCATGCAGGTGGTGCCGTCGCAGCGCATGTCCATCGAATTCAAGGCGGCGCCCATCGACCTGTACCGCGCGCTGCGCTGTTTCAATCCGACGCCCTACATGTACTTCTTCAACTTCGGCGACTTCCATGTCGTGGGCAGCTCGCCGGAGGTGCTGGTACGGGTCGAGGATGGCCTGGTGACGGTGCGCCCGATCGCCGGTACCCGTCCGCGCGGGATCAACGAAGAGGCCGACCTGGCACTGGAGCAGGATCTGCTGTCGGACGCCAAGGAGATCGCCGAGCACCTGATGCTGATCGACCTGGGGCGCAACGACGTGGGGCGGGTGTCCGATATCGGCGCGGTGAAGGTCACCGAAAAAATGGTGATCGAACGTTACTCCAACGTCATGCACATCGTGTCCAACGTCACCGGGCAATTGCGCGAGGGGCTCAGCGCGATGGACGCGCTGCGGGCGATTCTGCCGGCGGGCACTCTATCCGGCGCGCCGAAGATCCGCGCCATGGAGATCATCGACGAGCTGGAGCCGGTCAAGCGTGGAGTCTACGGCGGCGCGGTCGGCTACCTGGCATGGAACGGCAACATGGACACCGCCATTGCCATCCGCACCGCGGTGATCAAGAACGGTGAACTCCACGTGCAGGCCGGCGGCGGTATCGTTGCCGACTCGGTGCCCGCGCTGGAGTGGGAAGAAACCATCAACAA

**Strain no.725 ST 137**

*acs allel 31*

TAGCCGGTAGGCGCTCACCATTCTGTTCGAGGGCGTGCCGAACTACCCCGACGTGACCCGCGTGGCGAAAATCATCGACAAGCACAAGGTCAACATCCTCTACACCGCGCCGACCGCGATCCGCGCGATGATGGCCGAAGGCAAGGCGGCGGTGGCCGGTGCCGACGGTTCCAGCCTGCGTCTGCTCGGTTCGGTGGGCGAGCCGATCAACCCGGAAGCCTGGCAGTGGTACTACGAGACCGTCGGCCAGTCGCGCTGCCCGATCGTCGACACCTGGTGGCAGACCGAGACCGGCGCCTGCCTGATGACCCCGTTGCCGGGCGCCCATGCGATGAAGCCGGGCTCCGCGGCCAAGCCGTTCTTCGGCGTGGTCCCGGCGCTGGTGGACAACCTCGGCAACCTGATCGAGGGCGCCGCCGAGGGCAACCTGGTGATCCTCGACTCCTGGCCGGGCCAGGCGCGGACCCTGTTCGGCGACCATGACCGCTTCGTCGACACCTACTTCAAGACCTTCAAGGGCATGTACTTCACCGGCGACGGCGCGCGCCGCGACGAGGACGGCTACTACTGGATCACCGGGCGGGTCGACGACGTGCTCAACGTCTCCGGCCACCGCATGGGCACCGCCGAGGTGGAAAGCGCGATGGTCGCCCACCCGAAGGTCGCCGAGGCGGCGGTGGTCGGCATGCAGCACGACATCAAGGGGCAGGGATT

*aro allel 5*

GGGGCTAAGGATGGAACCCGAGCCGGGCAGCGAGGAAGCGCGCCTGACCGAGTATTTCCTCGCCGACCGCGATTGGCTCGCCGGCCAGCCCTGACCCGCCCGCAGGGGACGAGTCCGCTCGTCCCGTCCCACTCCGGCAAAGGAACGACCCATGGACCGCTATTGCGTATTCGGCAACCCCATCGGCCACAGCAAGTCGCCGCTGATCCACCGCCTGTTCGCCGAGCAGACCGGCGAGGCGCTGGTCTATGACGCGCAACTGGCGCCGCTGGACGATTTCCCCGGGTTCGCCCGGCGCTTCTTCGAGCAGGGCAAGGGCGCCAATGTCACCGTGCCGTTCAAGGAAGAGGCCTATCGTCTGGTGGACGAGTTGAGCGAGCGGGCCACCCGGGCCGGGGCGGTGAACACCCTGATCCGCCTCGCCGACGGTCGCCTGCGCGGCGACAACACCGACGGCGCCGGCCTGCTGCGGGACCTGACGGCGAACGCCGGGGTCGAGCTGCGCGGCAAGCGGGTTCTCCTGCTCGGCGCCGGCGGTGCGGTGCGTGGGGTGCTCGAACCCTTCCTCGGCGAGTGCCCGGCGGAGTTGCTGATCGCCAACCGCACGGCGCGGAAGGCCGTGGACCTGGCCGAGCGGTTCGCCGACCTCGGCGCGGTGCACGGCTGCGGTTTCGCCGAGGTCGAAGGGCCTTTCGACCTGATCGTCAACGGCACCTCGGCCAGTCTTGCCGGCGACGTGCCGCCGCTGGCGCAGAGCGTGATCGAGCCCGGCCGTACCGTCTGCTACGACATGATGTATGCCAAGGAACCGACTGCCTTCAACCGCTGGGCCGCCGAACGCGGTGCGGCGCGTACCCTGGATGGCCTGGGCATGCTGGTGGAGCAGGCCGCCGAGGCATTCTTCCTCTGGCGCGGCGTGCGTCCTGCCTCGGCGCCAGTGTTGGAGACGCTGCGCCGACAGTTGGCAACTGTCTGAGTTGTCAGGCGGCATTTGAGACGTACGTCTCAAATTGGCTTCCATCTGACATGTAGGAATCACAAACCCGGGTT

*gua allel 57*

CTGTGGATGAGCCACGGCGACAAGGTCACCGAGATGCCGGCCGGCTTCCACATCCTGGCCAGCACCCCGAGCTGCCCGATCGCCGCCATGGCCGACGATGCCCGCGCCTACTACGGCGTGCAATTCCACCCGGAAGTCACCCACACCAAGCAGGGCCTGCGCATTCTCTCGCGCTTCGTCCTCGACATCTGCGGTTGCGCCGCGCTGTGGACCCCGTCGAACATCGTCGACGACGCCATCGCTACCGTGCGCGCCCAGGTCGGTTCCTCCAAGGTCCTGCTCGGCCTCTCCGGTGGCGTGGACTCCTCGGTGGTCGCCGCGCTGCTGCACAAGGCCATCGGCGACCAACTGACCTGCGTGTTCGTCGACAACGGCCTGCTGCGTCTGCACGAAGGCGACCAGGTGATGGCCATGTTCGCCGAGAACATGGGCGTGAAGGTGATCCGCGCCAACGCCGAGGACAAGTTCCTCGGTCGCCTGGCCGGCGTCGCCGACCCGGAAGAGAAGCGCAAGATCATCGGCCGCACCTTCATCGAAGTCTTCGACGAAGAAGCCACCAAGCTGCAGGACGTGAAGTTCCTCGCCCAGGGCACCATCTACCCCGACGTGATCGAGTCGGCCGGCGCCAAGACCGGCAAGGCCCATGTGATCAAGTCGCACCACAACGTCGGCGGCCTGCCGGAGGACATGCAGTTCGAACTGGTCGAGCCGCTGCGCGAACTGTTCAAGGACGAAGTGCGCAAGATCGGCCTGGAGCTGGGCCTGCCCTACGACATGGTCTACCGCCACCCGTTCCCCGGCCCGGGCCTGGGCGTGCGCATCCTCGGCGAGGTGAAGAAGGAGTACGCCGACCTGCTGCGCCAGGCCGACCACATCTTCATCGAGGAACTGCGCGCCTTCGATTGGTACCACAAGACCAGCCAGGCGTT

*mut allel 13*

TCGACGTGGAGGTCGAGCAGGGCGGCATCAAGTTGCTGCGAGTGCGCGACGACGGTCGCGGCATCCCCGCCGACGACCTGCCGCTGGCCCTGGCTCGCCACGCCACCAGCAAGATCCGCGAGCTGGAAGACCTGGAGCGGGTGATGAGCCTCGGCTTCCGTGGCGAGGCGCTAGCCTCGATCAGCTCGGTAGCGCGCCTGACCATGACCTCGCGCACCGCCGACGCCGGCGAAGCCTGGCAGGTGGAAACCGAGGGCCGCGACATGCAGCCGCGGGTACAGCCGGCGGCGCACCCGGTGGGGACCAGCGTCGAGGTTCGCGACCTGTTCTTCAACACCCCGGCGCGGCGCAAGTTCCTGCGCGCCGAGAAGACCGAGTTCGACCATCTGCAGGAAGTCATCAAGCGCCTGGCGCTGGCCCGTTTCGACGTGGCTTTCCACCTGCGCCACAACGGCAAGACCATCTTCGCCCTGCACGAGGCGCGAGACGAGCTGGCCCGCGCGCGCCGGGTCGGCGCGGTGTGCGGCCAGGCATTCCTCGAGCAGGCGCTGCCGATCGAGGTCGAGCGCAACGGTCTGCACCTGTGGGGCTGGGTCGGCTTGCCGACCTTCTCCCGCAGCCAGCCGGACCTGCAGTACTTCTATGTGAACGGGCGCATGGTGCGCGACAAGCTGGTCGCCCACGCGGTGCGCCAGGCTTATCGCGACGTGCTGTACAACGGCCGGCATCCGACCTTCGTGCTGTTCTTCGAAGTCGATCCGGCGGTGGTGGACGTCAACGTGCACCCGACCAAGCACGAAGTTCGCTTCCGTGACAGCCGGATGGTCCATGACTTCCTCTATGGCACCCA

*nuo allel 1*

TACCGCCAACCCGTACTGGCCCAACGCCAACTGGTACGAGCGCGAAGTGTGGGACATGTACGGCATCACCTTCACCGGCCACCCGCACCTGACCCGCATGCTGATGCCGCCGACCTGGCAGGGCCATCCGCTGCGCAAGGACTACCCGGCGCGCGCCACCGAGTTCGATCCCTACTCCCTGTCCGCCGCCAAGCAGGACCTGGAGCAGGAGGCCCTGCGCTTCAAGCCGGAAGACTGGGGCATGAAGCGCCACGGCGAGAACGAGGACTACATGTTCCTCAACCTCGGCCCGAACCACCCGTCCGCCCACGGCGCGTTCCGCATCATCCTGCAACTGGACGGCGAGGAGATCATCGACTGCGTCCCGGAGATCGGCTACCACCACCGCGGCGCCGAGAAGATGGCCGAGCGCCAGTCCTGGCACAGTTTCATCCCCTACACCGACCGCATCGACTACCTCGGCGGGGTGATGAACAACCTGCCCTACGTACTCTCGGTGGAGAAGCTCGCCGGGATCAAGGTGCCGCAGCGGGTCGACGTGATCCGGATCATGATGGCGGAGTTCTTCCGTATCCTGAACCACCTGCTGTACCTGGGCACCTATATCCAGGACGTCGGCGCCATGACCCCGGTGTTCTTCACCTTCACCGACCGCCAGCGCGCCTACNAGGTGGTCGAGGCCATCACCGGCTTCCGCCTGCACCCGGCCTGGTACCGCATCGGCGGCGTCGCTCACGACCTGCCGCGCGGCTGGGACAAGCTGGTCCGCGAGTTCCTCGACTGGATGCCCAAGCGCCTCGACGAGTACGAGACCGCGGCCCTGAAGAACAGCATCCTGCGCGGCCGGACCAT

*pps allel 40*

GTCAAGGTAGTGGACGTCGATCGCGCCGATCGCGCCCGCTTCGCCCTGAGCGACGCCGAGGTCACCGAGCTGGCCAAGCAGGCCATGATCATCGAGAAGCACTATGGCCGCCCGATGGACATCGAATGGGCCAAGGACGGTGACGACGGCAAGCTGTACATCGTCCAGGCACGCCCGGAAACCGTGAAGAGCCGCGCCAGCGCCACGGTCATGGAGCGCTACCTGCTGAAAGAGAAGGGGACCGTCCTGGTGGAAGGACGTGCCATCGGCCAGCGCATCGGTGCCGGTCCGGTCAAGGTGATCAACGACGTGTCGGAAATGGACAAGGTCCAACCGGGTGACGTCCTGGTCTCCGACATGACCGACCCGGACTGGGAGCCGGTGATGAAGCGCGCCAGCGCCATCGTCACCAACCGCGGCGGGCGTACCTGCCACGCTGCGATCATCGCTCGCGAACTGGGCATCCCGGCGGTGGTCGGTTGCGGCAACGCCACCCAGATCCTGCAGGATGGGCAGGGGGTGACCGTTTCCTGTGCCGAAGGCGATACCGGCTTCATCTTCGAAGGCGAACTCGGTTTCGATGTGCGCAAGAACTCGGTCGACGCCATGCCCGACCTTCCGTTCAAGATCATGATGAACGTCGGCAATCCCGATCGCGCTTTCGATTTCGCCCAGTTGCCGAACGAAGGCGTGGGCCTGGCCCGCCTCGAATTCATCATCAACCGCATGATCGGCGTGCACCCCAAGGCGTTGCTGAACTTCGCCGGCCTGCCGGCGGACATCAAGGAAAGCGTGGAGAAGCGCATCGCCGGCTATCCCGATCCGGTCGGCTTCTACGTCGAGAAGCTGGTGGAGGGCATCAGCACCCTGGCCGCGGCGTTCTGGCCGAAGAAGGTCATCGTGCGCCTGTCCGACTTCAAGTCCAATGAGTACGCCAACCTGATCGGCGGCAAGCTCTACGAGCCGGAAG

*trp allel 3*

GGGTCGTGAGCCGGCGTTTCGTGCCAGCTTCACCCGCGAGGACTATGAAAACGCGGTAGGAAGGATCAAGGACTACATCCTGGCCGGCGACTGCATGCAGGTGGTGCCGTCGCAGCGCATGTCCATCGAGTTCAAGGCGGCGCCCATCGACCTGTACCGCGCGCTGCGCTGTTTCAATCCGACGCCCTACATGTACTTCTTCAACTTCGGCGACTTCCATGTCGTGGGCAGCTCGCCGGAGGTGCTGGTACGGGTCGAGGATGGCCTGGTGACGGTGCGCCCGATCGCCGGTACCCGTCCGCGCGGGATCAACGAAGAGGCCGACCTGGCGCTGGAGCAGGATCTGCTGTCGGACGCCAAGGAGATCGCCGAGCACCTGATGCTGATCGACCTGGGGCGCAACGACGTGGGGCGGGTGTCCGACATCGGCGCGGTGAAGGTCACCGAAAAAATGGTGATCGAACGTTACTCCAACGTCATGCACATCGTGTCCAACGTCACCGGGCAATTGCGCGAGGGGCTCAGCGCGATGGACGCGCTGCGGGCGATCCTGCCGGCGGGTACGCTGTCCGGCGCGCCGAAGATCCGCGCCATGGAGATCATCGACGAGCTGGAGCCGGTCAAGCGTGGAGTCTACGGCGGCGCGGTCGGCTACCTGGCATGGAACGGCAACATGGACACCGCCATTGCCATCCGCACCGCGGTGATCAAGAACGGTGAACTCCACGTGCAGGCCGGCGGCGGTATCGTTGCCGACTCGGTGCCGGCGCTGGAGTGGGAAGAAACCATCAACAA

**Strain no 764 ST 644**

*acs allel 28*

GCCTCGCTGACCCACGAGCGGGTYTTCGACTACCGTCCGGGCGAAGTCTACTGGTGCACCGCCGACATCGGCTGGGTCACCGGCCACACCTACATCGTCTATGGCCCGTTGGCCAACGGCGCCACCACCATTCTGTTCGAGGGCGTGCCGAACTACCCCGACGTGACCCGCGTGGCGAAAATCATCGACAAGCACAAGGTCAACATCCTCTACACCGCGCCGACCGCGATCCGCGCGATGATGGCCGAAGGCAAGGCGGCGGTGGCCGGTGCCGACGGTTCCAGCCTGCGTCTGCTCGGTTCGGTGGGCGAGCCGATCAACCCGGAAGCCTGGCAGTGGTACTACGAGACCGTCGGCCAGTCGCGCTGCCCGATCGTCGACACCTGGTGGCAGACCGAGACCGGCGCCTGCCTGATGACCCCGCTGCCGGGCGCCCACGCGATGAAGCCGGGCTCTGCAGCCAAGCCGTTCTTCGGCGTGGTACCGGCACTGGTGGACAATCTCGGCAACCTGATCGAGGGCGCCGCCGAGGGCAACCTGGTGATCCTCGACTCCTGGCCGGGCCAGGCGCGGACCCTGTTCGGCGACCATGACCGCTTCGTCGACACCTACTTCAAGACCTTCAAGGGCATGTACTTCACCGGCGACGGCGCGCGCCGCGACGAGGACGGCTACTACTGGATCACCGGGCGGGTCGACGACGTGCTCAACGTCTCCGGCCACCGCATGGGCACCGCCGAGGTGGAAAGCGCGATGGTCGCCCACCCGAAGGTCGCCGAGGCGGCGGTGGTCGGCATGCAGCACGACATCAAGGGGCAGG

*aro allel 3*

GGGCTAAGGATGGAAACCCGAGCCGGGCAGCGAGGAAGCGCGCCTGACCGAGTATTTCCTCGCCGACCGCGATTGGCTCGCCGGCCAGCCCTGACCCGCCCGCAGGGGACGAGTCCGCTCGTCCCGTCCCACCCCGGCAAAGGAACGACCCATGGACCGCTATTGCGTATTCGGCAACCCCATCGGCCACAGCAAGTCGCCGCTGATCCACCGCCTGTTCGCCGAGCAGACCGGCGAGGCGCTGGTCTATGACGCGCAGCCGGCGCCGCTGGACGATTTCCCCGGGTTCGCCCGGCGCTTCTTCGAGCAGGGCAAGGGCGCCAATGTCACCGTGCCGTTCAAGGAAGAGGCCTATCGTCTGGTGGACGAATTGAGCGAGCGGGCCACCCGGGCCGGGGCGGTGAACACCCTGATCCGCCTGGCCGACGGTCGCCTGCGCGGCGACAACACCGACGGCGCGGGCTTGCTGCGGGACCTGACGGCGAACGCCGGGGTCGAGCTGCGCGGCAAGCGGGTTCTCCTGCTCGGCGCCGGCGGTGCGGTGCGCGGGGTGCTCGAACCCTTCCTCGGCGAGTGCCCGGCGGAGCTGCTGATCGCCAACCGCACGGCGCGGAAGGCCGTGGATCTGGCCGAGCGGTTCGCCGATCTCGGCGCGGTGCGCGGCTGCGGTTTCGCCGAGGTCGAAGGGCCTTTCGACCTGGTCGTCAACGGCACCTCGGCCAGTCTTGCCGGCGACGTGCCGCCGCTGGCGCAGAGCGTGATCGAGCCCGGCCGTACCGTTTGCTACGACATGATGTATGCCAAGGAACCGACCGCCTTCAACCGCTGGGCCGCCGAACGCGGTGCGGCGCGTACCCTGGATGGCCTGGGCATGCTGGTGGAGCAGGCCGCCGAGGCATTCTTCCTCTGGCGTGGCGTGCGTCCTGCCTCGGCGCCAGTGTTGGAGACGCTGCGCCGGCAATTGGCAACTGTCTGAGTTGTCAGGCAGCATTTGAGACGTACGTCTCAAATCGGTTTCCATCTGACATGTAGGAATCACAAAACCCGGG

*gua allel 94*

CTGTGGATGAGCCACGGCGACAAGGTCACCGAGATGCCGGCCGGCTTCCACATCCTGGCCAGCACCCCGAGCTGCCCGATCGCCGCCATGGCCGACGATGCCCGCGCCTACTACGGCGTGCAATTCCACCCGGAAGTCACCCACACCAAGCAGGGCCTGCGCATTCTCTCGCGCTTCGTCCTCGACATCTGCGGTTGCGCCGCGTTGTGGACCCCCTCGAACATCGTCGACGACGCCATCGCCACCGTGCGCGCCCAGGTCGGTTCCTCCAAGGTCCTGCTCGGCCTCTCCGGTGGCGTGGACTCCTCGGTGGTCGCCGCGCTGCTGCACAAGGCCATCGGCGACCAACTGACCTGCGTGTTCGTCGACAACGGCCTGCTGCGCCTGCACGAAGGCGACCAGGTGATGGCCATGTTCGCCGAGAACATGGGCGTGAAGGTGATCCGCGCCAACGCCGAGGACAAGTTCCTCGGCCGCCTGGCCGGCGTCGCCGACCCGGAAGAGAAGCGCAAGATCATCGGCCGCACCTTCATCGAAGTCTTCGACGAAGAAGCCACCAAGCTGCAGGACGTGAAGTTCCTCGCCCAGGGCACCATCTACCCCGACGTGATCGAGTCGGCCGGCGCCAAGACCGGCAAGGCCCACGTGATCAAGTCGCACCACAACGTCGGCGGCCTGCCGGAGGACATGCAGTTCGAACTGGTCGAGCCGCTGCGCGAACTGTTCAAGGACGAAGTGCGCAAGATCGGCCTGGAGCTGGGCCTGCCCTACGACATGGTCTACCGCCACCCGTTCCCCGGCCCGGGCCTGGGCGTGCGCATCCTCGGCGAGGTGAAGAAGGAGTACGCCGACCTGCTGCGCCAGGCCGACCACATCTTCATCGAGGAACTGCGCGCCTTCGACTGGTACCACAAGACCAGCC

*mut allel 13*

CTGGCTCGCCACGCCACCAGCAAGATCCGCGAGCTGGAAGACCTGGAGCGGGTGATGAGCCTCGGCTTCCGTGGCGAGGCGCTGGCCTCGATCAGCTCGGTAGCGCGCCTGACCATGACCTCGCGCACCGCCGACGCCGGCGAAGCCTGGCAGGTGGAAACCGAGGGCCGCGACATGCAGCCGCGGGTACAGCCGGCGGCGCACCCGGTGGGGACCAGCGTCGAGGTTCGCGACCTGTTCTTCAACACCCCGGCGCGGCGCAAGTTCCTGCGTGCCGAGAAGACCGAGTTCGACCATCTGCAGGAAGTCATCAAGCGCCTGGCGCTGGCCCGTTTCGACGTGGCTTTCCACCTGCGCCACAACGGCAAGACCATCTTCGCCCTGCACGAGGCGCGAGACGAGCTGGCCCGCGCGCGCCGGGTCGGCGCGGTGTGCGGCCAGGCATTCCTCGAGCAGGCGCTGCCGATCGAGGTCGAGCGCAACGGTCTGCACCTGTGGGGCTGGGTCGGCTTGCCGACCTTCTCCCGCAGCCAGCCGGACCTGCAGTACTTCTATGTGAACGGGCGCATGGTGCGCGACAAGCTGGTCGCCCACGCGGTGCGCCAGGCTTATCGCGACGTGCTGTACAACGGCCGGCATCCGACCTTCGTGCTGTTCTTCGAAGTCGATCCGGCGGTGGTGGACGTCAACGTGCACCCGACCAAGCACGAAGTTCGCTTCCGTGACAGCCGGATGGTCCATGACTTCCTCTATGGGCACCC

*nuo allel 1*

CCCCGTACTGGCCCAACGCCAACTGGTACGAGCGCGAAGTGTGGGACATGTACGGCATCACCTTCACCGGCCACCCGCACCTGACCCGCATGCTGATGCCGCCGACCTGGCAGGGCCATCCGCTGCGCAAGGACTACCCGGCGCGCGCCACCGAGTTCGATCCCTACTCCCTGTCCGCCGCCAAGCAGGACCTGGAGCAGGAAGCCCTGCGCTTCAAGCCGGAAGACTGGGGCATGAAGCGCCACGGCGAGAACGAGGACTACATGTTCCTCAACCTCGGCCCGAACCACCCGTCCGCCCACGGCGCGTTCCGCATCATCCTGCAACTGGACGGCGAGGAGATCATCGACTGCGTCCCGGAGATCGGCTACCACCACCGCGGCGCCGAGAAGATGGCCGAGCGCCAGTCCTGGCACAGTTTCATCCCCTACACCGACCGCATCGACTACCTCGGCGGGGTGATGAACAACCTGCCCTACGTACTCTCGGTGGAGAAGCTCGCCGGGATCAAGGTGCCGCAGCGGGTCGACGTGATCCGGATCATGATGGCGGAGTTCTTCCGTATCCTGAACCACCTGCTGTACCTGGGCACCTATATCCAGGACGTCGGCGCCATGACCCCGGTGTTCTTCACCTTCACCGACCGCCAGCGCGCCTACAAGGTGGTCGAGGCCATCACCGGCTTCCGCCTGCACCCGGCCTGGTACCGCATCGGCGGCGTCGCCCACGACCTGCCGCGCGGCTGGGACAAGCTGGTCCGCGAGTTCCTCGACTGGATGCCCAAGCGCCTCGACGAGTACGAGACCGCGGCCCTGAAGAACAGCATCCTGCGCGGCCGGACCAT

*pps allel 4*

AAGGTAGTGGACGTCGATCGCGCCGATCGCGCCCGCTTCGCCCTGAGCGACGCCGAGGTCACCGAGCTGGCCAAGCAGGCCATGATCATCGAGAAGCACTATGGCCGCCCGATGGACATCGAATGGGCCAAGGACGGTGACGACGGCAAGCTGTACATCGTCCAGGCACGCCCGGAAACCGTGAAGAGCCGCGCCAGCGCCACGGTCATGGAGCGCTACCTGCTGAAAGAGAAGGGGACCGTCCTGGTGGAAGGGCGTGCCATCGGCCAGCGCATCGGTGCCGGTCCGGTCAAGGTGATCAACGACGTGTCGGAAATGGACAAGGTCCAACCGGGTGACGTCCTGGTCTCCGACATGACCGACCCGGACTGGGAGCCGGTGATGAAGCGCGCCAGCGCCATCGTCACCAACCGCGGCGGGCGTACCTGCCACGCGGCGATCATCGCTCGCGAACTGGGCATCCCGGCGGTGGTCGGTTGCGGCAACGCCACCCAGATCCTGCAGGATGGCCAGGGGGTGACCGTTTCCTGTGCCGAAGGCGATACCGGCTTCATCTTCGAAGGCGAACTCGGTTTCGATGTGCGCAAGAACTCGGTCGACGCCATGCCCGACCTTCCGTTCAAGATCATGATGAACGTCGGCAATCCCGATCGCGCTTTCGATTTCGCCCAGTTGCCGAACGAAGGCGTGGGCCTGGCCCGCCTCGAATTCATCATCAACCGCATGATCGGCGTGCATCCCAAGGCGTTGCTGAACTTCGCCGGCCTGCCGGCGGACATCAAGGAAAGCGTGGAGAAGCGCATCGCGGGCTATCCCGATCCGGTCGGCTTCTACGTCGAGAAACTGGTGGAGGGCATCAGCACCCTGGCCGCGGCGTTCTGGCC

*trp allel 10*

GGTCGTGAGCCGGCGTTTCGTGCCAGCTTCACCCGCGAGGACTATGAAAACGCGGTAGGAAGGATCAAGGACTACATCCTGGCCGGCGACTGCATGCAGGTGGTGCCGTCGCAGCGCATGTCCATCGAATTCAAGGCGGCGCCCATCGACCTGTACCGCGCGCTGCGCTGTTTCAATCCGACGCCCTACATGTACTTCTTCAACTTCGGCGACTTCCATGTCGTGGGCAGCTCGCCGGAGGTGCTGGTACGGGTCGAGGATGGCCTGGTGACGGTGCGCCCGATCGCCGGTACCCGTCCGCGCGGGATCAACGAAGAGGCCGACCTGGCGCTGGAGCAGGATCTGCTGTCGGACGCCAAGGAGATCGCCGAGCACCTGATGCTGATCGACCTGGGGCGCAACGACGTGGGGCGGGTGTCCGATATCGGCGCGGTGAAGGTCACCGAAAAAATGGTGATCGAACGTTACTCCAACGTCATGCACATCGTGTCCAACGTCACCGGGCAATTGCGCGAGGGGCTCAGCGCGATGGACGCGCTGCGGGCGATCCTGCCGGCGGGCACTCTATCCGGCGCGCCGAAGATCCGCGCCATGGAGATCATCGACGAGCTGGAGCCGGTCAAGCGTGGAGTCTACGGCGGCGCGGTCGGCTACCTGGCATGGAACGGCAACATGGACACCGCCATTGCCATCCGCACCGCGGTGATCAAGAACGGTGAACTCCACGTGCAGGCCGGCGGCGGTATCGTTGCCGACTCGGTGCCCGCGCTGGAGTGGGAAGAAACCATCAACAAGG

**Strain no. 779 ST260**

*acs allel 14*

GACTACCGTCCGGGCGAAGTCTACTGGTGCACCGCCGACATCGGCTGGGTCACCGGCCACACCTACATCGTCTATGGCCCGTTGGCCAACGGCGCCACCACCATTCTGTTCGAGGGCGTGCCGAACTACCCCGACGTGACCCGCGTGGCGAAAATCATCGACAAGCACAAGGTCAACATCCTCTACACCGCGCCGACCGCGATCCGCGCGATGATGGCTGAAGGCAAGGCGGCGGTGGCCGGTGCCGACGGTTCCAGCCTGCGTCTGCTCGGTTCGGTGGGCGAGCCGATCAACCCGGAAGCGTGGCAGTGGTACTACGAGGCCGTCGGCCAGTCGCGCTGCCCGATCGTCGACACCTGGTGGCAGACCGAGACCGGCGCCTGCCTGATGACCCCGTTGCCGGGCGCCCATGCGATGAAGCCGGGCTCCGCGGCCAAGCCGTTCTTCGGCGTGGTCCCGGCGCTGGTGGACAACCTCGGCAACCTGATCGAGGGCGCCGCCGAGGGCAACCTGGTGATCCTCGACTCCTGGCCGGGCCAGGCGCGGACCCTGTTCGGCGACCATGACCGCTTCGTCGACACCTACTTCAAGACCTTCAAGGGCATGTACTTCACCGGCGACGGCGCGCGCCGCGACGAGGACGGCTACTACTGGATCACCGGGCGGGTCGACGACGTGCTCAACGTCTCCGGCCACCGCATGGGCACCGCCGAGGTGGAAAGCGCGATGGTCGCCCACCCGAAGGTCGCCGAGGCGGCGGTGGTCGGCATGCAGCACGACATCAAGGGGCAGG

*aro allel 5*

GGGCTATGACTGGAAACCCGAGCCGGGCAGCGAGGAAGCGCGCCTGACCGAGTATTTCCTCGCCGACCGCGATTGGCTCGCCGGCCAGCCCTGACCCGCCCGCAGGGGACGAGTCCGCTCGTCCCGTCCCACTCCGGCAAAGGAACGACCCATGGACCGCTATTGCGTATTCGGCAACCCCATCGGCCACAGCAAGTCGCCGCTGATCCACCGCCTGTTCGCCGAGCAGACCGGCGAGGCGCTGGTCTATGACGCGCAACTGGCGCCGCTGGACGATTTCCCCGGGTTCGCCCGGCGCTTCTTCGAGCAGGGCAAGGGCGCCAATGTCACCGTGCCGTTCAAGGAAGAGGCCTATCGTCTGGTGGACGAGTTGAGCGAGCGGGCCACCCGGGCCGGGGCGGTGAACACCCTGATCCGCCTCGCCGACGGTCGCCTGCGCGGCGACAACACCGACGGCGCCGGCCTGCTGCGGGACCTGACGGCGAACGCCGGGGTCGAGCTGCGCGGCAAGCGGGTTCTCCTGCTCGGCGCCGGCGGTGCGGTGCGTGGGGTGCTCGAACCCTTCCTCGGCGAGTGCCCGGCGGAGTTGCTGATCGCCAACCGCACGGCGCGGAAGGCCGTGGACCTGGCCGAGCGGTTCGCCGACCTCGGCGCGGTGCACGGCTGCGGTTTCGCCGAGGTCGAAGGGCCTTTCGACCTGATCGTCAACGGCACCTCGGCCAGTCTTGCCGGCGACGTGCCGCCGCTGGCGCAGAGCGTGATCGAGCCCGGCCGTACCGTCTGCTACGACATGATGTATGCCAAGGAACCGACTGCCTTCAACCGCTGGGCCGCCGAACGCGGTGCGGCGCGTACCCTGGATGGCCTGGGCATGCTGGTGGAGCAGGCCGCCGAGGCATTCTTCCTCTGGCGCGGCGTGCGTCCTGCCTCGGCGCCAGTGTTGGAGACGCTGCGCCGACAGTTGGCAACTGTCTGAGTTGTCAGGCGGCATTTGAGACGTACGTCTCAAATTGGCTTCCATCTGACATGTAGGAATCACAAAACCGGG

*gua allel 10*

TGTGGATGAGCCACGGCGACAAGGTCACCGAGATGCCGGCCGGCTTCCACATCCTGGCCAGCACCCCGAGCTGCCCGATCGCCGCCATGGCCGACGATGCCCGCGCCTACTACGGCGTGCAATTCCACCCGGAAGTCACCCACACCAAGCAGGGCCTGCGCATTCTCTCGCGCTTCGTCCTCGACATCTGCGGTTGCGCCGCGCTGTGGACCCCCTCGAACATCGTCGACGACGCCATCGCCACCGTGCGCGCCCAGATCGGTTCCTCCAAGGTCCTGCTAGGCCTCTCCGGCGGCGTGGACTCCTCGGTGGTCGCCGCGCTGCTGCACAAGGCCATCGGCGACCAACTGACCTGCGTGTTCGTCGACAACGGCCTGCTGCGCCTGCACGAAGGCGACCAGGTGATGGCCATGTTCGCCGAGAACATGGGCGTGAAGGTGATCCGCGCCAACGCCGAGGACAAGTTCCTCGGCCGCCTGGCCGGCGTCGCCGATCCGGAAGAGAAGCGCAAGATCATCGGCCGCACCTTCATCGAAGTCTTCGACGAAGAAGCCACCAAGCTGCAGGACGTGAAGTTCCTCGCCCAGGGCACCATCTACCCCGACGTGATCGAGTCGGCCGGCGCCAAGACCGGCAAGGCCCACGTGATCAAGTCGCACCACAACGTCGGCGGCCTGCCGGAGGACATGCAGTTCGAACTGGTCGAGCCGCTGCGCGAACTGTTCAAGGACGAAGTGCGCAAGATCGGCCTGGAGCTGGGCCTGCCCTACGACATGGTCTACCGCCACCCGTTCCCCGGCCCGGGCCTGGGCGTGCGCATCCTCGGCGAGGTGAAGAAGGAGTACGCCGACCTGCTGCGCCAGGCCGACCACATCTTCATCGAGGAACTGCGCGCCTTCGACTGGTACCACAAGACCAGCC

*mut allel 7*

CGGCATCAAGTTGCTGCGAGTGCGCGACGACGGTCGCGGCATCCCCGCCGACGACCTGCCGCTGGCCCTGGCTCGCCACGCCACCAGCAAGATCCGCGAGCTGGAAGACCTGGAGCGGGTGATGAGCCTCGGCTTCCGTGGCGAGGCGCTGGCCTCGATCAGTTCGGTAGCGCGCCTGACCATGACCTCGCGTACCGCCGACGCCGGCGAAGCCTGGCAGGTGGAAACCGAGGGCCGTGACATGCAGCCGCGGGTACAGCCGGCGGCGCACCCGGTGGGGACCAGCGTCGAGGTTCGCGACCTGTTCTTCAACACCCCGGCGCGGCGCAAGTTCCTGCGCGCCGAGAAGACCGAGTTCGACCATCTGCAGGAAGTCATCAAGCGCCTGGCGCTGGCCCGTTTCGACGTGGCTTTCCACCTGCGCCACAACGGCAAGACCATCTTCGCCCTGCACGAGGCGCGAGACGAGCTGGCCCGCGCGCGCCGGGTCGGCGCGGTGTGCGGCCAGGCATTCCTCGAGCAGGCGCTGCCGATCGAGGTCGAGCGCAACGGCCTGCACCTGTGGGGTTGGGTCGGCTTGCCGACCTTCTCTCGCAGCCAGCCGGACCTGCAGTACTTCTATGTGAACGGGCGCATGGTGCGCGACAAGCTGGTCGCCCACGCGGTGCGCCAGGCTTATCGCGACGTGCTGTACAACGGCCGGCATCCGACCTTCGTGCTGTTCTTCGAAGTCGATCCGGCGGTGGTGGACGTCAACGTGCACCCGACCAAGCACGAAGTTCGCTTCCGTGACAGCCGGATGGTCCATGACTTCCTCTATGGCACCC

*nuo allel 4*

CCGCCAACCCGTACTGGCCCAACGCCAACTGGTACGAGCGCGAAGTGTGGGACATGTACGGCATCACCTTCACCGGCCACCCGCACCTGACCCGCATGCTGATGCCGCCGACCTGGCAGGGCCATCCGCTGCGCAAGGACTACCCGGCGCGCGCCACCGAGTTCGATCCCTACTCCCTGTCCGCCGCCAAGCAGGACCTGGAGCAGGAAGCCCTGCGCTTCAAGCCGGAAGACTGGGGCATGAAGCGCCACGGCGAGAACGAGGACTACATGTTCCTCAACCTCGGCCCGAACCACCCGTCCGCCCACGGCGCGTTCCGCATCATCCTGCAACTGGACGGCGAGGAGATCATCGACTGCGTCCCGGAGATCGGCTACCACCACCGCGGCGCCGAGAAGATGGCCGAGCGCCAGTCCTGGCACAGTTTCATCCCCTACACCGACCGCATCGACTACCTCGGCGGGGTGATGAACAACCTGCCCTACGTACTCTCGGTGGAGAAGCTCGCCGGGATCAAGGTGCCCCAGCGGGTCGACGTGATCCGGATCATGATGGCGGAGTTCTTCCGTATCCTGAACCACCTGCTGTACCTGGGCACCTATATCCAGGACGTCGGCGCCATGACCCCGGTGTTCTTCACCTTCACCGACCGCCAGCGCGCCTACAAGGTGGTCGAGGCCATCACCGGCTTCCGCCTGCACCCGGCCTGGTACCGCATCGGCGGCGTCGCCCACGACCTGCCGCGCGGCTGGGACAAGCTGGTCCGCGAGTTCCTCGACTGGATGCCCAAGCGCCTCGACGAGTACGAGACCGCGGCCCTGAAGAACAGCATC

*pps allel 13*

CGCTCGGTCAAGGTAGTGGACGTCGATCGCGCCGATCGCGCCCGCTTCGCCCTGAGCGACGCCGAGGTCACCGAGCTGGCCAAGCAGGCCATGATCATCGAGAAGCACTATGGCCGCCCGATGGACATCGAATGGGCCAAGGACGGTGACGACGGCAAGCTGTACATCGTCCAGGCACGCCCGGAAACCGTGAAGAGCCGCGCCAGCGCCACGGTCATGGAGCGCTACCTGCTGAAAGAGAAGGGGACCGTCCTGGTGGAAGGGCGTGCCATCGGCCAGCGCATCGGTGCCGGTCCGGTCAAAGTGATCAACGACGTGTCGGAAATGGACAAGGTCCAACCGGGTGACGTCCTGGTCTCCGACATGACCGACCCGGACTGGGAGCCAGTGATGAAGCGCGCCAGCGCCTTCGTCACCAACCGCGGCGGGCGCACCTGCCACGCGGCGATCATCGCTCGCGAACTGGGCATCCCGGCGGTGGTCGGTTGCGGCAACGCCACCCAGATCCTGCAGGATGGCCAGGGGGTGACCGTTTCCTGTGCCGAAGGCGATACCGGCTTCATCTTCGAAGGCGAACTCGGTTTCGATGTGCGCAAGAACTCGGTCGACGCCTGCCCGACCTTCCGTTCAAGATCATGATGAACGTCGGCAATCCCGATCGCGCTTTCGATTTCGCCCAGTTGCCGAACGAAGGCGTGGGCCTGGCCCGCCTCGAATTCATCATCAACCGCATGATCGGCGTGCATCCCAAGGCGTTGCTGAACTTCGCCGGCCTGCCGGCGGACATCAAGGAAAGCGTGGAGAAGCGCATCGCCGGCTATCCCGATCCGGTCGGCTTCTACGTCGAGAAGCTGGTGGAGGGCATCAGCACCCTGGCCGCGGCGTTCTGGCCGAAGAAGGTCATCGTGCG

*trp allel 7*

GGTCGTGAGCCGGCGTTTCGTGCCAGCTTCACCCGCGAGGACTATGAAAACGCGGTAGGAAGGATCAAGGACTACATCCTGGCCGGCGACTGCATGCAGGTGGTGCCGTCGCAGCGCATGTCCATCGAATTCAAGGCGGCGCCCATCGACCTGTACCGCGCGCTGCGCTGTTTCAATCCGACGCCCTACATGTACTTCTTCAACTTCGGCGACTTCCATGTCGTGGGCAGCTCGCCGGAGGTGCTGGTACGGGTCGAGGATGGCCTGGTGACGGTGCGCCCGATCGCCGGTACCCGTCCGCGCGGGATCAACGAAGAGGCCGACCTGGCACTGGAGCAGGATCTGCTGTCGGACGCCAAGGAGATCGCCGAGCACCTGATGCTGATCGACCTGGGGCGCAACGACGTGGGGCGGGTGTCCGATATCGGCGCGGTGAAGGTCACCGAAAAAATGGTGATCGAACGTTACTCCAACGTCATGCACATCGTGTCCAACGTCACCGGGCAATTGCGCGAGGGGCTCAGCGCGATGGACGCGCTGCGGGCGATTCTGCCGGCGGGCACTCTATCCGGCGCGCCGAAGATCCGCGCCATGGAGATCATCGACGAGCTGGAGCCGGTCAAGCGTGGAGTCTACGGCGGCGCGGTCGGCTACCTGGCATGGAACGGCAACATGGACACCGCCATTGCCATCCGCACCGCGGTGATCAAGAACGGTGAACTCCACGTGCAGGCCGGCGGCGGTATCGTTGCCGACTCGGTGCCCGCGCTGGAGTGGGAAGAAACCATCAAC

**Strain no 783 ST253**

*acs allel 4*

GACTACCGTCCGGGCGAAGTCTACTGGTGCACCGCCGACATCGGCTGGGTCACCGGCCACACCTACATCGTCTATGGCCCGTTGGCCAACGGCGCCACCACCATTCTGTTCGAGGGCGTACCGAACTACCCCGACGTGACCCGCGTGGCGAAGATCATCGACAAGCACAAGGTTAACATCCTCTACACCGCGCCGACCGCGATCCGCGCGATGATGGCCGAAGGCAAGGCGGCGGTGGCCGGTGCCGACGGTTCCAGCCTGCGTCTGCTCGGTTCGGTGGGCGAGCCGATCAACCCGGAAGCCTGGCAGTGGTACTACGAGACCGTCGGCCAGTCGCGCTGCCCGATCGTCGACACCTGGTGGCAGACCGAGACCGGCGCCTGCCTGATGACCCCGTTGCCGGGCGCCCATGCGATGAAGCCGGGCTCCGCGGCCAAGCCGTTCTTCGGCGTGGTCCCGGCGCTGGTGGACAACCTCGGCAACCTGATCGAAGGCGCCGCCGAGGGCAACCTGGTGATCCTCGACTCCTGGCCGGGCCAGGCGCGGACCCTGTTCGGCGACCATGACCGCTTCGTCGACACCTACTTCAAGACCTTCAAGGGCATGTACTTCACCGGCGACGGCGCGCGCCGCGACGAGGACGGCTACTACTGGATCACCGGGCGGGTCGACGACGTGCTCAACGTCTCCGGCCACCGCATGGGCACCGCCGAGGTGGAAAGCGCGATGGTCGCCCACCCGAAGGTCGCCGAGGCGGCGGTGGTCGGCATGCAGCACGACATCAAGGGGCAGGGA

*aro allel 4*

CCGGGCAGCGAGGAAGCGCGCCTGACCGAGTATTTCCTCGCCGACCGCGATTGGCTCGCCGGCCAGCCCTGACCCGCCCGCAGGGGACGAGTCCGCTCGTCCCGTCCCACCCCGGCAAAGGAACGACCCATGGACCGCTACTGCGTATTCGGCAACCCCATCGGCCACAGCAAGTCGCCGCTGATCCACCGCCTGTTCGCCGAGCAGACCGGCGAGGCGCTGGTCTATGACGCGCAGCTGGCGCCGCTGGACGATTTCCCCGGGTTCGCCCGGCGCTTCTTCGAGCAGGGCAAGGGCGCCAATGTCACCGTGCCGTTCAAGGAAGAGGCCTATCGTCTGGTGGACGAATTGAGCGAGCGGGCCACCCGGGCCGGGGCGGTGAACACCCTGATCCGCCTGGCCGACGGTCGCCTGCGCGGCGACAACACCGACGGCGCGGGCTTGCTGCGGGACCTGACGGCGAACGCCGGGGTCGAGCTGCGCGGCAAGCGGGTTCTCCTGCTCGGCGCCGGCGGTGCGGTGCGCGGGGTGCTCGAACCCTTCCTCGGCGAGTGCCCGGCGGAGTTGCTGATCGCCAACCGCACGGCGCGGAAGGCCGTGGACCTGGCCGAGCGATTCGCCGATCTCGGCGCGGTGCGCGGCTGCGGTTTCGCCGAGGTCGAAGGGCCTTTCGACCTGGTCGTCAACGGCACCTCGGCCAGTCTTGCCGGCGACGTGCCGCCGCTGGCGCAGAGCGTGATCGAGCCCGGCCGTACCGTCTGCTACGACATGATGTATGCCAAGGAACCGACCGCCTTCAACCGCTGGGCCGCCGAACGCGGTGCGGCGCGTACCCTGGATGGCCTGGGCATGCTGGTGGAGCAGGCCGC

*gua allel 16*

CGGTGGATGAGCCACGGCGACAAGGTCACCGAGATGCCGGCCGGCTTCCACATCCTGGCCAGCACCCCGAGCTGCCCGATCGCCGCCATGGCCGACGATGCCCGCGCCTACTACGGCGTGCAATTCCACCCGGAAGTCACCCACACCAAGCAGGGCCTGCGCATTCTCTCGCGCTTCGTCCTCGACATCTGTGGTTGCGCCGCGCTGTGGACCCCCTCGAACATCGTCGACGACGCCATCGCCACCGTGCGCGCCCAGGTCGGTTCCTCCAAGGTCCTGCTCGGCCTCTCCGGCGGCGTGGACTCCTCGGTGGTCGCCGCGCTGCTGCACAAGGCCATCGGCGACCAACTGACCTGCGTGTTCGTCGACAACGGCCTGCTGCGCCTGCACGAAGGCGACCAGGTGATGGCCATGTTCGCCGAGAACATGGGCGTGAAGGTGATCCGCGCCAACGCCGAGGACAAGTTCCTCGGCCGCCTGGCCGGCGTCGCCGATCCGGAAGAGAAGCGCAAGATCATCGGCCGCACCTTCATCGAAGTCTTCGACGAAGAAGCCACCAAGCTGCAGGACGTGAAGTTCCTCGCCCAGGGCACCATCTACCCCGACGTGATCGAGTCGGCCGGCGCCAAAACCGGCAAGGCCCACGTGATCAAGTCGCACCACAACGTCGGCGGCCTGCCGGAGGACATGCAGTTCGAACTGGTCGAGCCGCTGCGCGAACTGTTCAAGGACGAAGTGCGCAAGATCGGCCTGGAGCTGGGCCTGCCCTACGACATGGTCTACCGCCACCCGTTCCCCGGCCCGGGCCTGGGCGTGCGCATCCTCGGCGAGGTGAAGAAGGAGTACGCCGACCTGCTGCGCCAGGCCGACCACATCTTCATCGAGGAACTGCGCGCCTTCGACTGGTACCACAAGACCAGCC

*mut allel 12*

GCGGCATCCCCGCCGACGACCTGCCGCTGGCCCTGGCTCGCCACGCCACCAGCAAGATCCGCGAGCTGGAAGACCTGGAGCGGGTGATGAGCCTCGGCTTCCGTGGCGAGGCGCTGGCCTCGATCAGCTCGGTAGCGCGCCTGACCATGACCTCGCGTACCGCCGACGCCGGCGAAGCCTGGCAGGTGGAAACCGAGGGCCGCGACATGCAGCCGCGGGTACAGCCGGCGGCGCACCCGGTGGGGACCAGCGTCGAGGTTCGCGACCTGTTCTTCAACACCCCGGCGCGGCGCAAGTTCCTGCGCGCTGAGAAGACCGAGTTCGACCATCTGCAGGAAGTCATCAAGCGCCTGGCGCTGGCCCGTTTCGACGTGGCTTTCCACCTGCGCCACAACGGCAAGACCATCTTCGCCCTGCACGAGGCGCGAGACGAGCTGGCCCGCGCGCGCCGGGTCGGCGCGGTGTGCGGCCAGGCATTCCTCGAGCAGGCGCTGCCGATCGAGGTCGAGCGCAACGGCCTGCACCTGTGGGGCTGGGTCGGCTTGCCGACCTTCTCCCGCAGCCAGCCGGACCTGCAGTACTTCTATGTGAACGGGCGCATGGTGCGCGACAAGCTGGTCGCCCACGCGGTGCGCCAGGCTTATCGCGACGTGCTGTACAACGGCCGGCACCCGACCTTCGTGCTGTTCTTCGAAGTCGATCCGGCGGTGGTGGACGTCAACGTGCACCCGACCAAGCACGAAGTTCGCTTCCGTGACAGCCGGATGGTCCATGACTTCCTCTATGGGCACCC

*nuo allel 1*

GTACTGGCCCAACGCCAACTGGTACGAGCGCGAAGTGTGGGACATGTACGGCATCACCTTCACCGGCCACCCGCACCTGACCCGCATGCTGATGCCGCCGACCTGGCAGGGCCATCCGCTGCGCAAGGACTACCCGGCGCGCGCCACCGAGTTCGATCCCTACTCCCTGTCCGCCGCCAAGCAGGACCTGGAGCAGGAGGCCCTGCGCTTCAAGCCGGAAGACTGGGGCATGAAGCGCCACGGCGAGAACGAGGACTACATGTTCCTCAACCTCGGCCCGAACCACCCGTCCGCCCACGGCGCGTTCCGCATCATCCTGCAACTGGACGGCGAGGAGATCATCGACTGCGTCCCGGAGATCGGCTACCACCACCGCGGCGCCGAGAAGATGGCCGAGCGCCAGTCCTGGCACAGTTTCATCCCCTACACCGACCGCATCGACTACCTCGGCGGGGTGATGAACAACCTGCCCTACGTACTCTCGGTGGAGAAGCTCGCCGGGATCAAGGTGCCGCAGCGGGTCGACGTGATCCGGATCATGATGGCGGAGTTCTTCCGTATCCTGAACCACCTGCTGTACCTGGGCACCTATATCCAGGACGTCGGCGCCATGACCCCGGTGTTCTTCACCTTCACCGACCGCCAGCGCGCCTACAAGGTGGTCGAGGCCATCACCGGCTTCCGCCTGCACCCGGCCTGGTACCGCATCGGCGGCGTCGCCCACGACCTGCCGCGCGGCTGGGACAAGCTGGTCCGCGAGTTCCTCGACTGGATGCCCAAGCGCCTCGACGAGTACGAGACCGCGGCCCTGAAGAACAGCATCCTGCGCGGCCGGACCATCGGCGTCGCCCAGTACAACACCAAGGAA

*pps allel 6*

CGGTCAAGGTAGTGGACGTCGATCGCGCCGATCGCGCCCGCTTCGCCCTGAGCGACGCCGAGGTCACCGAGCTGGCCAAGCAGGCCATGATCATCGAGAAGCACTATGGCCGCCCGATGGACATCGAATGGGCCAAGGACGGTGACGACGGCAAGCTGTACATCGTCCAGGCACGCCCGGAAACCGTGAAGAGCCGCGCCAGCGCCACGGTCATGGAGCGCTACCTGCTGAAAGAGAAGGGGACCGTCCTGGTGGAAGGACGTGCCATCGGCCAGCGCATCGGTGCCGGTCCGGTCAAGGTGATCAACGACGTGTCGGAAATGGACAAGGTCCAACCGGGTGACGTCCTGGTCTCCGACATGACCGACCCGGACTGGGAGCCGGTGATGAAGCGCGCCAGCGCCATCGTCACCAACCGCGGCGGGCGTACCTGCCACGCGGCGATCATCGCTCGCGAACTGGGCATCCCGGCGGTGGTCGGTTGCGGCAACGCCACCCAGTCCTGCAGGATGGGCAGGGGGTGACCGTTTCCTGTGCCGAAGGCGATACCGGCTTCATCTTCGAAGGCGAACTCGGTTTCGATGTGCGCAAGAACTCGGTCGACGCCATGCCCGACCTTCCGTTCAAGATCATGATGAACGTCGGCAATCCCGATCGCGCTTTCGATTTCGCCCAGTTGCCGAACGAAGGCGTGGGCCTGGCCCGCCTCGAATTCATCATCAACCGCATGATCGGCGTGCACCCCAAGGCGTTGCTGAACTTCGCCGGCCTGCCGGCGGACATCAAGGAAAGCGTGGAGAAGCGCATCGCCGGCTATCCCGATCCGGTCGGCTTCTACGTCGA

*trp allel 3*

GGGTCGTGAGCCGGCGTTTCGTGCCAGCTTCACCCGCGAGGACTATGAAAACGCGGTAGGAAGGATCAAGGACTACATCCTGGCCGGCGACTGCATGCAGGTGGTGCCGTCGCAGCGCATGTCCATCGAGTTCAAGGCGGCGCCCATCGACCTGTACCGCGCGCTGCGCTGTTTCAATCCGACGCCCTACATGTACTTCTTCAACTTCGGCGACTTCCATGTCGTGGGCAGCTCGCCGGAGGTGCTGGTACGGGTCGAGGATGGCCTGGTGACGGTGCGCCCGATCGCCGGTACCCGTCCGCGCGGGATCAACGAAGAGGCCGACCTGGCGCTGGAGCAGGATCTGCTGTCGGACGCCAAGGAGATCGCCGAGCACCTGATGCTGATCGACCTGGGGCGCAACGACGTGGGGCGGGTGTCCGACATCGGCGCGGTGAAGGTCACCGAAAAAATGGTGATCGAACGTTACTCCAACGTCATGCACATCGTGTCCAACGTCACCGGGCAATTGCGCGAGGGGCTCAGCGCGATGGACGCGCTGCGGGCGATCCTGCCGGCGGGTACGCTGTCCGGCGCGCCGAAGATCCGCGCCATGGAGATCATCGACGAGCTGGAGCCGGTCAAGCGTGGAGTCTACGGCGGCGCGGTCGGCTACCTGGCATGGAACGGCAACATGGACACCGCCATTGCCATCCGCACCGCGGTGATCAAGAACGGTGAACTCCACGTGCAGGCCGGCGGCGGTATCGTTGCCGACTCGGTGCCGGCGCTGGAGTGGGAAGAAACCATC

**Strain no 786 ST253**

*acs allel 4*

GCCAACGGCGCCACCACCATTCTGTTCGAGGGCGTACCGAACTACCCCGACGTGACCCGCGTGGCGAAGATCATCGACAAGCACAAGGTTAACATCCTCTACACCGCGCCGACCGCGATCCGCGCGATGATGGCCGAAGGCAAGGCGGCGGTGGCCGGTGCCGACGGTTCCAGCCTGCGTCTGCTCGGTTCGGTGGGCGAGCCGATCAACCCGGAAGCCTGGCAGTGGTACTACGAGACCGTCGGCCAGTCGCGCTGCCCGATCGTCGACACCTGGTGGCAGACCGAGACCGGCGCCTGCCTGATGACCCCGTTGCCGGGCGCCCATGCGATGAAGCCGGGCTCCGCGGCCAAGCCGTTCTTCGGCGTGGTCCCGGCGCTGGTGGACAACCTCGGCAACCTGATCGAAGGCGCCGCCGAGGGCAACCTGGTGATCCTCGACTCCTGGCCGGGCCAGGCGCGGACCCTGTTCGGCGACCATGACCGCTTCGTCGACACCTACTTCAAGACCTTCAAGGGCATGTACTTCACCGGCGACGGCGCGCGCCGCGACGAGGACGGCTACTACTGGATCACCGGGCGGGTCGACGACGTGCTCAACGTCTCCGGCCACCGCATGGGCACCGCCGAGGTGGAAAGCGCGATGGTCGCCCACCCGAAGGTCGCCGAGGCGGCGGTGGTCGGCATGCAGCACGACATCAAGGGGCAGGGA

*aro allel 4*

GGGGCTATGGACTGGAAACCCGAGCCGGGCAGCGAGGAAGCGCGCCTGACCGAGTATTTCCTCGCCGACCGCGATTGGCTCGCCGGCCAGCCCTGACCCGCCCGCAGGGGACGAGTCCGCTCGTCCCGTCCCACCCCGGCAAAGGAACGACCCATGGACCGCTACTGCGTATTCGGCAACCCCATCGGCCACAGCAAGTCGCCGCTGATCCACCGCCTGTTCGCCGAGCAGACCGGCGAGGCGCTGGTCTATGACGCGCAGCCGGCGCCGCTGGACGATTTCCCCGGGTTCGCCCGGCGCTTCTTCGAGCAGGGCAAGGGCGCCAATGTCACCGTGCCGTTCAAGGAAGAGGCCTATCGTCTGGTGGACGAATTGAGCGAGCGGGCCACCCGGGCCGGGGCGGTGAACACCCTGATCCGCCTGGCCGACGGTCGCCTGCGCGGCGACAACACCGACGGCGCGGGCTTGCTGCGGGACCTGACGGCGAACGCCGGGGTCGAGCTGCGCGGCAAGCGGGTTCTCCTGCTCGGCGCCGGCGGTGCGGTGCGCGGGGTGCTCGAACCCTTCCTCGGCGAGTGCCCGGCGGAGTTGCTGATCGCCAACCGCACGGCGCGGAAGGCCGTGGACCTGGCCGAGCGATTCGCCGATCTCGGCGCGGTGCGCGGCTGCGGTTTCGCCGAGGTCGAAGGGCCTTTCGACCTGGTCGTCAACGGCACCTCGGCCAGTCTTGCCGGCGACGTGCCGCCGCTGGCGCAGAGCGTGATCGAGCCCGGCCGTACCGTCTGCTACGACATGATGTATGCCAAGGAACCGACCGCCTTCAACCGCTGGGCCGCCGAACGCGGTGCGGCGCGTACCCTGGATGGCCTGGGCATGCTG

*gua allel 16*

TGGATGAGCCACGGCGACAAGGTCACCGAGATGCCGGCCGGCTTCCACATCCTGGCCAGCACCCCGAGCTGCCCGATCGCCGCCATGGCCGACGATGCCCGCGCCTACTACGGCGTGCAATTCCACCCGGAAGTCACCCACACCAAGCAGGGCCTGCGCATTCTCTCGCGCTTCGTCCTCGACATCTGTGGTTGCGCCGCGCTGTGGACCCCCTCGAACATCGTCGACGACGCCATCGCCACCGTGCGCGCCCAGGTCGGTTCCTCCAAGGTCCTGCTCGGCCTCTCCGGCGGCGTGGACTCCTCGGTGGTCGCCGCGCTGCTGCACAAGGCCATCGGCGACCAACTGACCTGCGTGTTCGTCGACAACGGCCTGCTGCGCCTGCACGAAGGCGACCAGGTGATGGCCATGTTCGCCGAGAACATGGGCGTGAAGGTGATCCGCGCCAACGCCGAGGACAAGTTCCTCGGCCGCCTGGCCGGCGTCGCCGATCCGGAAGAGAAGCGCAAGATCATCGGCCGCACCTTCATCGAAGTCTTCGACGAAGAAGCCACCAAGCTGCAGGACGTGAAGTTCCTCGCCCAGGGCACCATCTACCCCGACGTGATCGAGTCGGCCGGCGCCAAAACCGGCAAGGCCCACGTGATCAAGTCGCACCACAACGTCGGCGGCCTGCCGGAGGACATGCAGTTCGAACTGGTCGAGCCGCTGCGCGAACTGTTCAAGGACGAAGTGCGCAAGATCGGCCTGGAGCTGGGCCTGCCCTACGACATGGTCTACCGCCACCCGTTCCCCGGCCCGGGCCTGGGCGTGCGCATCCTCGGCGAGGTGAAGAAGGAGTACGCCGACCTGCTGCGCCAGGCCGACCACATCTTCATCGAGGAACTGCGCGCCTTCGACTGGTA

*mut allel 12*

GCGGCATCCCCGCCGACGACCTGCCGCTGGCCCTGGCTCGCCACGCCACCAGCAAGATCCGCGAGCTGGAAGACCTGGAGCGGGTGATGAGCCTCGGCTTCCGTGGCGAGGCGCTGGCCTCGATCAGCTCGGTAGCGCGCCTGACCATGACCTCGCGTACCGCCGACGCCGGCGAAGCCTGGCAGGTGGAAACCGAGGGCCGCGACATGCAGCCGCGGGTACAGCCGGCGGCGCACCCGGTGGGGACCAGCGTCGAGGTTCGCGACCTGTTCTTCAACACCCCGGCGCGGCGCAAGTTCCTGCGCGCTGAGAAGACCGAGTTCGACCATCTGCAGGAAGTCATCAAGCGCCTGGCGCTGGCCCGTTTCGACGTGGCTTTCCACCTGCGCCACAACGGCAAGACCATCTTCGCCCTGCACGAGGCGCGAGACGAGCTGGCCCGCGCGCGCCGGGTCGGCGCGGTGTGCGGCCAGGCATTCCTCGAGCAGGCGCTGCCGATCGAGGTCGAGCGCAACGGCCTGCACCTGTGGGGCTGGGTCGGCTTGCCGACCTTCTCCCGCAGCCAGCCGGACCTGCAGTACTTCTATGTGAACGGGCGCATGGTGCGCGACAAGCTGGTCGCCCACGCGGTGCGCCAGGCTTATCGCGACGTGCTGTACAACGGCCGGCACCCGACCTTCGTGCTGTTCTTCGAAGTCGATCCGGCGGTGGTGGACGTCAACGTGCACCCGACCAAGCACGAAGTTCGCTTCCGTGACAGCCGGATGGTCCATGACTTCCTCTATGGGCACCC

*nuo allel 1*

GTACTGGCCCAACGCCAACTGGTACGAGCGCGAAGTGTGGGACATGTACGGCATCACCTTCACCGGCCACCCGCACCTGACCCGCATGCTGATGCCGCCGACCTGGCAGGGCCATCCGCTGCGCAAGGACTACCCGGCGCGCGCCACCGAGTTCGATCCCTACTCCCTGTCCGCCGCCAAGCAGGACCTGGAGCAGGAGGCCCTGCGCTTCAAGCCGGAAGACTGGGGCATGAAGCGCCACGGCGAGAACGAGGACTACATGTTCCTCAACCTCGGCCCGAACCACCCGTCCGCCCACGGCGCGTTCCGCATCATCCTGCAACTGGACGGCGAGGAGATCATCGACTGCGTCCCGGAGATCGGCTACCACCACCGCGGCGCCGAGAAGATGGCCGAGCGCCAGTCCTGGCACAGTTTCATCCCCTACACCGACCGCATCGACTACCTCGGCGGGGTGATGAACAACCTGCCCTACGTACTCTCGGTGGAGAAGCTCGCCGGGATCAAGGTGCCGCAGCGGGTCGACGTGATCCGGATCATGATGGCGGAGTTCTTCCGTATCCTGAACCACCTGCTGTACCTGGGCACCTATATCCAGGACGTCGGCGCCATGACCCCGGTGTTCTTCACCTTCACCGACCGCCAGCGCGCCTACAAGGTGGTCGAGGCCATCACCGGCTTCCGCCTGCACCCGGCCTGGTACCGCATCGGCGGCGTCGCCCACGACCTGCCGCGCGGCTGGGACAAGCTGGTCCGCGAGTTCCTCGACTGGATGCCCAAGCGCCTCGACGAGTACGAGACCGCGGCCCTGAAGAACAGCATCCTGCGCGGCCGGAC

*pps allel 6*

GTCGCTCGGTCAAGGTAGTGGACGTCGATCGCGCCGATCGCGCCCGCTTCGCCCTGAGCGACGCCGAGGTCACCGAGCTGGCCAAGCAGGCCATGATCATCGAGAAGCACTATGGCCGCCCGATGGACATCGAATGGGCCAAGGACGGTGACGACGGCAAGCTGTACATCGTCCAGGCACGCCCGGAAACCGTGAAGAGCCGCGCCAGCGCCACGGTCATGGAGCGCTACCTGCTGAAAGAGAAGGGGACCGTCCTGGTGGAAGGACGTGCCATCGGCCAGCGCATCGGTGCCGGTCCGGTCAAGGTGATCAACGACGTGTCGGAAATGGACAAGGTCCAACCGGGTGACGTCCTGGTCTCCGACATGACCGACCCGGACTGGGAGCCGGTGATGAAGCGCGCCAGCGCCATCGTCACCAACCGCGGCGGGCGTACCTGCCACGCGGCGATCATCGCTCGCGAACTGGGCATCCCGGCGGTGGTCGGTTGCGGCAACGCCACCCAGTCCTGCAGGATGGGCAGGGGGTGACCGTTTCCTGTGCCGAAGGCGATACCGGCTTCATCTTCGAAGGCGAACTCGGTTTCGATGTGCGCAAGAACTCGGTCGACGCCATGCCCGACCTTCCGTTCAAGATCATGATGAACGTCGGCAATCCCGATCGCGCTTTCGATTTCGCCCAGTTGCCGAACGAAGGCGTGGGCCTGGCCCGCCTCGAATTCATCATCAACCGCATGATCGGCGTGCACCCCAAGGCGTTGCTGAACTTCGCCGGCCTGCCGGCGGACATCAAGGAAAGCGTGGAGAAGCGCATCGCCGGCTATCCCGATCCGGTCGGCTTCTACGTCGAGAAGCTGGTGGAGGGCATCAG

*trp allel 3*

GCCGGCGTTTCGTGCCAGCTTCACCCGCGAGGACTATGAAAACGCGGTAGGAAGGATCAAGGACTACATCCTGGCCGGCGACTGCATGCAGGTGGTGCCGTCGCAGCGCATGTCCATCGAGTTCAAGGCGGCGCCCATCGACCTGTACCGCGCGCTGCGCTGTTTCAATCCGACGCCCTACATGTACTTCTTCAACTTCGGCGACTTCCATGTCGTGGGCAGCTCGCCGGAGGTGCTGGTACGGGTCGAGGATGGCCTGGTGACGGTGCGCCCGATCGCCGGTACCCGTCCGCGCGGGATCAACGAAGAGGCCGACCTGGCGCTGGAGCAGGATCTGCTGTCGGACGCCAAGGAGATCGCCGAGCACCTGATGCTGATCGACCTGGGGCGCAACGACGTGGGGCGGGTGTCCGACATCGGCGCGGTGAAGGTCACCGAAAAAATGGTGATCGAACGTTACTCCAACGTCATGCACATCGTGTCCAACGTCACCGGGCAATTGCGCGAGGGGCTCAGCGCGATGGACGCGCTGCGGGCGATCCTGCCGGCGGGTACGCTGTCCGGCGCGCCGAAGATCCGCGCCATGGAGATCATCGACGAGCTGGAGCCGGTCAAGCGTGGAGTCTACGGCGGCGCGGTCGGCTACCTGGCATGGAACGGCAACATGGACACCGCCATTGCCATCCGCACCGCGGTGATCAAGAACGGTGA

**Strain no.892 ST 966**

*asc allel 17*

CGCCTCGCTGACCCACGAGCGGGTCTTCGACTACCGTCCGGGCGAAGTCTACTGGTGCACCGCCGACATCGGCTGGGTCACCGGCCACACCTACATCGTCTATGGCCCGCTGGCCAACGGCGCCACCACCATTCTGTTCGAGGGCGTGCCGAACTACCCCGACGTGACCCGCGTGGCGAAAATCATCGACAAGCACAAGGTCAACATCCTCTACACCGCGCCGACCGCGATCCGCGCGATGATGGCCGAAGGCAAGGCGGCGGTGGCCGGTGCCGACGGTTCCAGCCTGCGTCTGCTCGGTTCGGTGGGCGAGCCGATCAACCCGGAAGCCTGGCAGTGGTACTACGAGACCGTCGGCCAGTCGCGCTGCCCGATCGTCGACACCTGGTGGCAGACCGAGACCGGCGCCTGCCTGATGACCCCGCTGCCGGGCGCCCACGCGATGAAGCCGGGCTCTGCAGCCAAGCCGTTCTTCGGCGTGGTACCGGCACTGGTGGACAACCTCGGCAACCTGATCGAGGGCGCCGCCGAGGGCAACCTGGTGATCCTCGACTCCTGGCCGGGCCAGGCGCGGACCCTGTTCGGCGACCATGACCGCTTCGTCGACACCTACTTCAAGACCTTCAAGGGCATGTACTTCACCGGCGACGGCGCGCGCCGCGACGAGGACGGCTACTACTGGATCACCGGGCGGGTCGACGACGTGCTCAACGTCTCCGGCCACCGCATGGGCACCGCCGAGGTGGAAAGCGCGATGGTCGCCCACCCGAAGGTCGCCGAGGCGGCGGTGGTCGGCATGCAGCACGACTTCAAGGGGCAGGGCA

*aro allel 3*

CTAAGGATGGAAACCCGAGCCGGGCAGCGAGGAAGCGCGCCTGACCGAGTATTTCCTCGCCGACCGCGATTGGCTCGCCGGCCAGCCCTGACCCGCCCGCAGGGGACGAGTCCGCTCGTCCCGTCCCACCCCGGCAAAGGAACGACCCATGGACCGCTATTGCGTATTCGGCAACCCCATCGGCCACAGCAAGTCGCCGCTGATCCACCGCCTGTTCGCCGAGCAGACCGGCGAGGCGCTGGTCTATGACGCGCAGCCGGCGCCGCTGGACGATTTCCCCGGGTTCGCCCGGCGCTTCTTCGAGCAGGGCAAGGGCGCCAATGTCACCGTGCCGTTCAAGGAAGAGGCCTATCGTCTGGTGGACGAATTGAGCGAGCGGGCCACCCGGGCCGGGGCGGTGAACACCCTGATCCGCCTGGCCGACGGTCGCCTGCGCGGCGACAACACCGACGGCGCGGGCTTGCTGCGGGACCTGACGGCGAACGCCGGGGTCGAGCTGCGCGGCAAGCGGGTTCTCCTGCTCGGCGCCGGCGGTGCGGTGCGCGGGGTGCTCGAACCCTTCCTCGGCGAGTGCCCGGCGGAGCTGCTGATCGCCAACCGCACGGCGCGGAAGGCCGTGGATCTGGCCGAGCGGTTCGCCGATCTCGGCGCGGTGCGCGGCTGCGGTTTCGCCGAGGTCGAAGGGCCTTTCGACCTGGTCGTCAACGGCACCTCGGCCAGTCTTGCCGGCGACGTGCCGCCGCTGGCGCAGAGCGTGATCGAGCCCGGCCGTACCGTTTGCTACGACATGATGTATGCCAAGGAACCGACCGCCTTCAACCGCTGGGCCGCCGAACGCGGTGCGGCGCGTACCCTGGATGGCCTGG

*gua allel 5*

GTGGATGAGCCACGGCGACAAGGTCACCGAGATGCCGGCCGGCTTCCACATCCTGGCCAGCACCCCGAGCTGCCCGATCGCCGCCATGGCCGACGATGCCCGCGCCTACTACGGCGTGCAATTCCACCCGGAAGTCACCCACACCAAGCAGGGCCTGCGCATTCTCTCGCGCTTCGTCCTCGACATCTGTGGTTGCGCCGCGCTGTGGACCCCCTCGAACATCGTCGACGACGCCATCGCCACCGTGCGCGCCCAGGTCGGTTCCTCCAAGGTCCTGCTCGGCCTCTCCGGCGGCGTGGACTCCTCGGTGGTCGCCGCGCTGCTGCACAAGGCCATCGGCGACCAACTGACCTGCGTGTTCGTCGACAACGGCCTGCTGCGCCTGCACGAAGGCGACCAGGTGATGGCCATGTTCGCCGAGAACATGGGCGTGAAGGTGATCCGCGCCAACGCCGAGGACAAGTTCCTCGGCCGCCTGGCCGGCGTCGCCGATCCGGAAGAGAAGCGCAAGATCATCGGCCGCACCTTCATCGAAGTCTTCGACGAAGAAGCCACCAAGCTGCAGGACGTGAAGTTCCTCGCCCAGGGCACCATCTACCCCGACGTGATCGAGTCGGCCGGCGCCAAGACCGGCAAGGCCCACGTGATCAAGTCGCACCACAACGTCGGCGGCCTGCCGGAGGACATGCAGTTCGAACTGGTCGAGCCGCTGCGCGAACTGTTCAAGGACGAAGTGCGCAAGATCGGCCTGGAGCTGGGCCTGCCCTACGACATGGTCTACCGCCACCCGTTCCCCGGCCCGGGCCTGGGCGTGCGCATCCTCGGCGAGGTGAAGAAGGAGTACGCCGACCTGCTGCGCCAGGCCGACCACATCTTCATCGAGGAACTGCGCGCCTTCGACTGGTACCACAAG

*mut allel4*

GCCTTGACGCCGGTTCCCGGCGCATTGATGTGGAGGTCGAGCAGGGCGGCATCAAGTTGCTGCGAGTGCGCGACGACGGTCGCGGCATCCCCGCCGACGACCTGCCGCTGGCCCTGGCTCGCCACGCCACCAGCAAGATCCGCGAGCTGGAAGACCTGGAGCGGGTGATGAGCCTCGGCTTCCGTGGCGAGGCGCTGGCCTCGATCAGCTCGGTAGCGCGCCTGACCATGACCTCGCGTACCGCCGACGCCGGCGAAGCCTGGCAGGTGGAAACCGAGGGCCGCGACATGCAGCCGCGGGTACAGCCGGCGGCGCACCCGGTGGGGACCAGCGTCGAGGTTCGCGACCTGTTCTTCAACACCCCGGCGCGGCGCAAGTTCCTGCGTGCCGAGAAGACCGAGTTCGACCATCTGCAGGAGGTCATCAAGCGCCTGGCGCTGGCCCGCTTCGACGTGGCTTTCCACCTGCGCCACAACGGCAAGACCATCTTCGCCCTGCACGAGGCGCGAGACGAGCTGGCCCGCGCGCGCCGGGTCGGCGCGGTGTGCGGCCAGGCATTCCTCGAGCAGGCGCTGCCGATCGAGGTCGAGCGCAACGGCCTGCACCTGTGGGGCTGGGTCGGCTTGCCGACCTTCTCCCGCAGCCAGCCGGACCTGCAGTACTTCTATGTGAACGGGCGCATGGTGCGCGACAAGCTGGTCGCCCACGCGGTGCGCCAGGCTTATCGCGACGTGCTGTACAACGGCCGGCATCCGACCTTCGTGCTGTTCTTCGAAGTCGATCCGGCGGTGGTGGACGTCAACGTGCACCCGACCAAGCACGAAGTTCGCTTCCGTGACAGCCGGATGATCCATGACTTCCTCTATGGCACCC

*nuo allel 4*

CGCGCCACCGAGTTCGATCCCTACTCCCTGTCCGCCGCCAAGCAGGACCTGGAGCAGGAAGCCCTGCGCTTCAAGCCGGAAGACTGGGGCATGAAGCGCCACGGCGAGAACGAGGACTACATGTTCCTCAACCTCGGCCCGAACCACCCGTCCGCCCACGGCGCGTTCCGCATCATCCTGCAACTGGACGGCGAGGAGATCATCGACTGCGTCCCGGAGATCGGCTACCACCACCGCGGCGCCGAGAAGATGGCCGAGCGCCAGTCCTGGCACAGTTTCATCCCCTACACCGACCGCATCGACTACCTCGGCGGGGTGATGAACAACCTGCCCTACGTACTCTCGGTGGAGAAGCTCGCCGGGATCAAGGTGCCCCAGCGGGTCGACGTGATCCGGATCATGATGGCGGAGTTCTTCCGTATCCTGAACCACCTGCTGTACCTGGGCACCTATATCCAGGACGTCGGCGCCATGACCCCGGTGTTCTTCACCTTCACCGACCGCCAGCGCGCCTACAAGGTGGTCGAGGCCATCACCGGCTTCCGCCTGCACCCGGCCTGGTACCGCATCGGCGGCGTCGCCCACGACCTGC

*pps allel 4*

TCAAGGTAGTGGACGTCGATCGCGCCGATCGCGCCCGCTTCGCCCTGAGCGACGCCGAGGTCACCGAGCTGGCCAAGCAGGCCATGATCATCGAGAAGCACTATGGCCGCCCGATGGACATCGAATGGGCCAAGGACGGTGACGACGGCAAGCTGTACATCGTCCAGGCACGCCCGGAAACCGTGAAGAGCCGCGCCAGCGCCACGGTCATGGAGCGCTACCTGCTGAAAGAGAAGGGGACCGTCCTGGTGGAAGGGCGTGCCATCGGCCAGCGCATCGGTGCCGGTCCGGTCAAGGTGATCAACGACGTGTCGGAAATGGACAAGGTCCAACCGGGTGACGTCCTGGTCTCCGACATGACCGACCCGGACTGGGAGCCGGTGATGAAGCGCGCCAGCGCCATCGTCACCAACCGCGGCGGGCGTACCTGCCACGCGGCGATCATCGCTCGCGAACTGGGCATCCCGGCGGTGGTCGGTTGCGGCAACGCCACCCAGATCCTGCAGGATGGCCAGGGGGTGACCGTTTCCTGTGCCGAAGGCGATACCGGCTTCATCTTCGAAGGCGAACTCGGTTTCGATGTGCGCAAGAACTCGGTCGACGCCATGCCCGACCTTCCGTTCAAGATCATGATGAACGTCGGCAATCCCGATCGCGCTTTCGATTTCGCCCAGTTGCCGAACGAAGGCGTGGGCCTGGCCCGCCTCGAATTCATCATCAACCGCATGATCGGCGTGCACCCCAAGGCATTGCTGAACTTCGCCGGCCTGCCGGCGGACATCAAGGAAAGCGTGGAGAAGCGCATCGCCGGCTATCCCGATCCGGTCGGCTTCTACGTCGAGAAGCTGGTGGAAGGCATCAGCACCCTGGCCGCGGCGTTCTGGCCGAAGA

*trp allel 3*

CGTGAGCCGGCGTTTCGTGCCAGCTTCACCCGCGAGGACTATGAAAACGCGGTAGGAAGGATCAAGGACTACATCCTGGCCGGCGACTGCATGCAGGTGGTGCCGTCGCAGCGCATGTCCATCGAATTCAAGGCGGCGCCCATCGACCTGTACCGCGCGCTGCGCTGTTTCAATCCGACGCCCTACATGTACTTCTTCAACTTCGGCGACTTCCATGTCGTGGGCAGCTCGCCGGAGGTGCTGGTACGGGTCGAGGATGGCCTGGTGACGGTGCGCCCGATCGCCGGTACCCGTCCGCGCGGGATCAACGAAGAGGCCGACCTGGCGCTGGAGCAGGATCTGCTGTCGGACGCCAAGGAGATCGCCGAGCACCTGATGCTGATCGACCTGGGGCGCAACGACGTGGGGCGGGTGTCCGACATCGGCGCGGTGAAGGTCACCGAAAAAATGGTGATCGAACGTTACTCCAACGTCATGCACATCGTGTCCAACGTCACCGGGCAATTGCGCGAGGGGCTCAGCGCGATGGACGCGCTGCGGGCGATCCTGCCGGCGGGTACGCTGTCCGGCGCGCCGAAGATCCGCGCCATGGAGATCATCGACGAGCTGGAGCCGGTCAAGCGTGGAGTCTACGGCGGCGCGGTCGGCTACCTGGCATGGAACGGCAACATGGACACCGCCATTGCCATCCGCACCGCGGTGATCAAGAACGGTGAACTCCACGTGCAGGCCGGCGGCGGTATCGTTGCCGACTCGGTGCCGGCGCTGGAG
