# Supplementary material for: Multi-locus sequence typing (MLST) of non-fermentative Gram-negative bacilli isolated from bloodstream infections in southern Poland
Source: Folia Microbiol (Praha). 2017 Sep 22;63(2):191–6. doi: 10.1007/s12223-017-0550-7 (PMC5805803; doi:10.1007/s12223-017-0550-7)
Supplement: Supplementary file 4 — (DOCX 31 kb) [file 12223_2017_550_MOESM4_ESM.docx]

**Nucleotide sequences Stenotrophomonas maltophilia strains**

**Strain no. 3**

*atpD allel 76*

CAGGGCAAGATCGTTCTGCTGTTCGTCGACAACATCTACCGCTACACCCTGGCCGGTACCGAAGTGTCGGCACTGCTGGGCCGTATGCCGTCCGCCGTGGGTTACCAGCCGACCCTGGCCGAGGAAATGGGCGTCCTGCAGGAACGCGGCATCTCCGTGCCGGTCGGCGCTGGCACCCTGGGCCGCATCATGGACGTGCTCGGCCGTCCGATCGACGAAGCCGGCCCGGTGGCCGCCAGCGACAGCTGGGAAATCCACCGTGCGGCCCCGTCGTACGAAGACCAGTCCCCGGCCACCGAACTGCTGGAAACCGGCATCAAGGTCATCGACCTGATGTGCCCGTTCGCCAAGGGCGGCAAGGTCGGCCTGTTCGGCGGCGCCGGCGTCGGCAAGACCGTCAACATGATGGAACTGATCAACAACATCGCCAAGGCGCACAGCGGTCTGTCCGTGTTCGCCGGCGTGGGTGAGCGTACCCGTGAGGGCAACGACTTCTACCACGAAATGAAGGACTCCAACGTCCTGGACAAGGTGGCGATGGTGTACGGCCAGATGAACGAGCCGCCGGGCAACCGTCTGCGCGTCGCCCTGACCGGCCTGACCATGGCCGAGTACTTCCGCGACGAGAAGGACGAAAACGGCAAGGGCAAGGACGTTCTGCTGTTCGT

*gapA allel 68*

CGATGCCGGCCTCGGCGTCGAACACCGAGGTGCAGGTCTCGCCGCGGAAATCGGTGGCCACGACCTTGTCTTCGGTGTAGCCGAGGATGCCCTTCAGCGGGCCTTCGCTCTGTGCCTTCACTTCAGCGCAGATCTCGGCGTAGGTGGCTTCCTTTTCCAGCTCGACGGTCAGGTCGACCACCGACACGTCCGAGGTCGGGACACGGAAGCTCATGCCGGTCAGCTTCTTGTTCAGTTCCGGGATGACCACGCCGACAGCCTTGGCCGCACCGGTGGACGACGGGATGATGTTCTCCAGGATGCCGCGGCCACCACGCCAGTCCTTGTTGGACGGGCCATCGACGGTCTTCTGGGTGGCAGTAGCCGCATGCACGGTGGTCATCAGGCCACGCTTGATGCCCCACTTGTCATTGATGACCTTGGCCAGCGGGGCCAGGCAGTTGGTGGTGCACGACGCGTTGGAAATGATGGCCTGGCCGGCGTAGGTCTTGTCGTTCACGCCGTAGACGAACATCGGCGTATCGTCCTTCGACGGGGCCGACATGATGACCTTCCTGGCGCCGGCATCGATGTGCTTCTGCGCGGTTTCCTTGGTCAGGAACAGGCCGGTGGACTCGATCACCACGTCCACGTCCACTTCGTTCCACTTCAGGTTGGCCGGGTCGCGTTCCTGGGTCAGGCGGATCTTCTTGCCGTTGACCAGCAGGTCGTTGCCCTGCACCGCCACGTCGGCCTTGAAACGACCGTGCACGGAGTCGTACTTGAGCATGTACGCCAGATAGTCCGGCTCCAGCAGATCGTTGATGGCC

*guaA allel 7*

CGCTGGTACGGCGTGCAGTTCCACCCGGAAGTGACCCACACCCTGCAGGGCCAGGCGCTGCTGCGCCGCTTCGTGGTGGACGTGTGCGGCTGCCAGACCCTGTGGACCGCCGCCAACATCATCGACGACCAGATCGCCCGCGTGCGCGAACAGGTGGGCGATGACGAAGTGATCCTGGGCCTGTCCGGCGGCGTCGATTCGTCCGTGGTGGCCGCGCTGCTGCACAAGGCCATCGGCGAAAAGCTGACCTGCGTGTTCGTGGATACCGGCCTGCTGCGCTGGCAGGAAGGCGACCAGGTGATGGCGATGTTCGCCGAACACATGGGCGTGAAGGTCGTTCGCGTGAATGCCGCCGACCGTTACTTCGCCGCGCTGGAAGGCGTGAGCGACCCGGAAGCCAAGCGCAAGATCATCGGTAACCTGTTCGTTGAGATCTTCGATGAAGAGTCGAACAAGCTGAGCAACGCCAAGTGGCTGGCGCAGGGCACCATCTACCCGGACGTGATCGAGTCGGCTGGCAGCAAGACCGGCAAGGCGCATGTGATCAAGAGCCACCACAACGTGGGCGGCCTGCCAGAGCACATGAAGCTGGGCCTGGTGGAGCCGCTGCGCGAGCTGTTCAAGGACGAAGTGCGCCGCCTGGGTGTTGAACTGGGCCTGCCGCGCACCATGGTCTC

*mutM allel 7*

TAACGTCCTGCGCTCGGCACCTGCAGGGCCGCCGCGTGCATGGCGTGATCCTGCGCCGCGCCGACCTGCGCTGGCCGATTCCGCCGGAAGTGGCCGAGCTGCTGCCGGGGCAGCGCATCGAGGACATCCGCCGTCGCGCCAAGTACCTGCTGCTGGACACCGCCATCGGCAGCGCCGTGCTGCACCTGGGCATGTCCGGCAGCCTGCGTGTGCTGCCCGGCGATACCCCGCTGCGCGCGCACGACCATGTGGATATCAGCCTGGACAACGGCCGCCTGTTGCGCTTCAACGACCCGCGCCGTTTCGGCAGCCTGCTCTGGCAGCCGGCCGGCGAAGTCCACCCGCTGCTGCAGGGGCTGGGCCCGGAGCCGCTGGACGATGCCTTCGACGGGGACTACCTGTTCTCCCGCAGCCGTGGCCGCAGCGCGCCGGTGAAGACCTTCCTGATGGACCAGGCGGTGGTGGTGGGCGTGGGCAACATCACGCCGACGCCGGTATCGCCCTG

*nuoD allel 80*

*AACCATGAACTTCGGCCCGCAGCATCCGGCCGCGCACGGTGTGCTGCGCCTGATCCTGGAGATGGACGGCGAAACCATCATGCGCGCCGACCCGCACGTGGGTCTGCTGCACCGTGGTACCGAAAAGCTGGCCGAGTCCAAGCCGTTCAACCAGTCGATCGGCTACATGGATCGCCTGGATTACGTGTCGATGATGTGCAACGAGCACGCCTACGTGCGCGCGATCGAGACCCTGATGGGCATCGAGGCGCCGGAGCGTGCGCAGTACATCCGCACCATGTACGACGAAATCACCCGCATCCTCAACCACCTGATGTGGCTTGGCTCCAATGCGCTCGATCTGGGTGCGATGGCGGTGATGCTGTACGCCTTCCGCGAGCGCGAAGAGCTGATGGACTGCTATGAAGCGGTCTCCGGCGCGCGCATGCACGCGGCGTACTACCGTCCGGGCGGTGTCTACCGCGACCTGCCGGACCACATGCCGAAGTACAAGA*

*ppsA allel 93*

TAGCCACGTTACATGACCGTGCGCGGCGTAAGAGCTGCGCAACACCTTCTCGATCAGCGACGAGGACGTGCAGGAACTGTCCAAGCAGGCGCTGGTCATCGAAAAGCACTACGGCCGCCCGATGGACATCGAGTGGGCCAAGGACGGTGTCAGCGGTAAGCTGTTCATCGTGCAGGCGCGCCCGGAAACGGTGAAGTCGCGCAGCCACGCCACCCAGATCGAGCGCTTCGCGCTGACCGAAAAGGGCGGCAACGTGCTGGCCGAAGGCCGTGCCGTCGGTGCCAAGATCGGTTCGGGCGTGGCCCGCGTGGTCAAGACGCTGGACGACATGAACCGCGTGCAGCCGGGCGACGTGCTGATCGCCGACATGACCGACCCGGACTGGGAACCGGTGATGAAGCGCGCTTCGGCCATCGTCACCAATCGCGGTGGCCGTACCTGCCACGCCGCGATCATCGCGCGCGAGCTGGGCGTGCCGGCCGTGGTCGGTTCGGGCAACGCCACCAAGGTCATCGAAGATGTCAACAGAGAGCAAKGGAAAAAAACGACTAAGA

*recA allel 74*

GCAGCTCTGGGCCAGATCGAAAAGCAGTTCGGCAAGGGCTCGGTGATGCGCATGGGCGACCGCGTGGTCGAGCCCGTCGAAGCCATCCCGACCGGTTCGCTGATGCTCGACATCGCACTGGGCATCGGCGGTCTGCCGAAGGGCCGTGTCGTCGAGATCTACGGGCCGGAATCCTCGGGCAAGACCACGCTGACCCTGCAGGCCATCGCCGAATGCCAGAAGATGGGCGGCACCGCGGCCTTCATCGACGCCGAGCACGCGCTGGACCCGATCTACGCCGCCAAGCTGGGCGTGAACGTGGACGACCTGCTGCTGTCGCAGCCGGATACCGGTGAGCAGGCGCTGGAAATCGCCGACATGCTGGTCCGTTCGGGTTCGGTCGACATCCTGGTGATCGACTCGGTCGCCGCGTTGACCCCGAAGGCTGAAATCGAAGGCGAGATGGGCGATCAGCTGCCGGGCCTGCAGGCCCGCCTGATGAGCCAGGCACTGCGCAAGCTGACCGGCAACATCAAGCGCTCCAACACCCTGGTGGTCTTCATCAACCAGCTGCGCATGAAGATCGGTGTGATGATGCCGGGCCAGAGCCCGGAAACCACCACCGGCGGCAACGCGCTGAAGTTCTATGCCTCGGTGCGCCTGGACATCCGCCGTATCGGCGCGATCAAGAAGGGCGACGAGATCATCGGCAACCAGACCAAGATCAAGGTCGTCAAGAACAAGCTGGCGCCTCCGTTCAAG

**Strain no. 14**

*atpD allel 76*

CCGTATGCCGTCCGCCGTGGGTTACCAGCCGACCCTGGCCGAGGAAATGGGCGTCCTGCAGGAACGCGGCATCTCCGTGCCGGTCGGCGCTGGCACCCTGGGCCGCATCATGGACGTGCTCGGCCGTCCGATCGACGAAGCCGGCCCGGTGGCCGCCAGCGACAGCTGGGAAATCCACCGTGCGGCCCCGTCGTACGAAGACCAGTCCCCGGCCACCGAACTGCTGGAAACCGGCATCAAGGTCATCGACCTGATGTGCCCGTTCGCCAAGGGCGGCAAGGTCGGCCTGTTCGGCGGCGCCGGCGTCGGCAAGACCGTCAACATGATGGAACTGATCAACAACATCGCCAAGGCGCACAGCGGTCTGTCCGTGTTCGCCGGCGTGGGTGAGCGTACCCGTGAGGGCAACGACTTCTACCACGAAATGAAGGACTCCAACGTCCTGGACAAGGTGGCGATGGTGTACGGCCAGATGAACGAGCCGCCGGGCAACCGTCTGCGCGTCGCCCTGACCGGCCTGACCATGGCCGAGTACTTCCGCGACGAGAAGGACGAAAACGGCAAGGGCAAGGACGTTCTGCTGTTCGT

*gapA allel 68*

ATGCCCTTCAGCGGGCCTTCGCTCTGTGCCTTCACTTCAGCGCAGATCTCGGCGTAGGTGGCTTCCTTTTCCAGCTCGACGGTCAGGTCGACCACCGACACGTCCGAGGTCGGGACACGGAAGCTCATGCCGGTCAGCTTCTTGTTCAGTTCCGGGATGACCACGCCGACAGCCTTGGCCGCACCGGTGGACGACGGGATGATGTTCTCCAGGATGCCGCGGCCACCACGCCAGTCCTTGTTGGACGGGCCATCGACGGTCTTCTGGGTGGCAGTAGCCGCATGCACGGTGGTCATCAGGCCACGCTTGATGCCCCACTTGTCATTGATGACCTTGGCCAGCGGGGCCAGGCAGTTGGTGGTGCACGACGCGTTGGAAATGATGGCCTGGCCGGCGTAGGTCTTGTCGTTCACGCCGTAGACGAACATCGGCGTATCGTCCTTCGACGGGGCCGACATGATGACCTTCCTGGCGCCGGCATCGATGTGCTTCTGCGCGGTTTCCTTGGTCAGGAACAGGCCGGTGGACTCGATCACCACGTCCACGTCCACTTCGTTCCACTTCAGGTTGGCCGGGTCGCGTTCCTGGGTCAGGCGGATCTTCTTGCCGTTGACCAGCAGGTCGTTGCCCTGCACCGCCACGTCGGCCTTGAAACGACCGTGCACGGAGTCGTACTTGAGCATGTACGCCAGATAGTCCGGCTCCAGCAGATCGTTGA

*guaA allel 7*

CGCTGGTACGGCGTGCAGTTCCACCCGGAAGTGACCCACACCCTGCAGGGCCAGGCGCTGCTGCGCCGCTTCGTGGTGGACGTGTGCGGCTGCCAGACCCTGTGGACCGCCGCCAACATCATCGACGACCAGATCGCCCGCGTGCGCGAACAGGTGGGCGATGACGAAGTGATCCTGGGCCTGTCCGGCGGCGTCGATTCGTCCGTGGTGGCCGCGCTGCTGCACAAGGCCATCGGCGAAAAGCTGACCTGCGTGTTCGTGGATACCGGCCTGCTGCGCTGGCAGGAAGGCGACCAGGTGATGGCGATGTTCGCCGAACACATGGGCGTGAAGGTCGTTCGCGTGAATGCCGCCGACCGTTACTTCGCCGCGCTGGAAGGCGTGAGCGACCCGGAAGCCAAGCGCAAGATCATCGGTAACCTGTTCGTTGAGATCTTCGATGAAGAGTCGAACAAGCTGAGCAACGCCAAGTGGCTGGCGCAGGGCACCATCTACCCGGACGTGATCGAGTCGGCTGGCAGCAAGACCGGCAAGGCGCATGTGATCAAGAGCCACCACAACGTGGGCGGCCTGCCAGAGCACATGAAGCTGGGCCTGGTGGAGCCGCTGCGCGAGCTGTTCAAGGACGAAGTGCGCCGCCTGGGTGTTGAACTGGGCCTGCCGCGCACCATGGTCT

*mutM allel 7*

TAACGTCCTGCGCTCGGCACCTGCAGGGCCGCCGCGTGCATGGCGTGATCCTGCGCCGCGCCGACCTGCGCTGGCCGATTCCGCCGGAAGTGGCCGAGCTGCTGCCGGGGCAGCGCATCGAGGACATCCGCCGTCGCGCCAAGTACCTGCTGCTGGACACCGCCATCGGCAGCGCCGTGCTGCACCTGGGCATGTCCGGCAGCCTGCGTGTGCTGCCCGGCGATACCCCGCTGCGCGCGCACGACCATGTGGATATCAGCCTGGACAACGGCCGCCTGTTGCGCTTCAACGACCCGCGCCGTTTCGGCAGCCTGCTCTGGCAGCCGGCCGGCGAAGTCCACCCGCTGCTGCAGGGGCTGGGCCCGGAGCCGCTGGACGATGCCTTCGACGGGGACTACCTGTTCTCCCGCAGCCGTGGCCGCAGCGCGCCGGTGAAGACCTTCCTGATGGACCAGGCGGTGGTGGTGGGCGTGGGCAACATCACGCCGACGCCGGTATCGCCC

*nuoD allel 80*

CTAACCATGAACTTCGGCCCGCAGCATCCGGCCGCGCACGGTGTGCTGCGCCTGATCCTGGAGATGGACGGCGAAACCATCATGCGCGCCGACCCGCACGTGGGTCTGCTGCACCGTGGTACCGAAAAGCTGGCCGAGTCCAAGCCGTTCAACCAGTCGATCGGCTACATGGATCGCCTGGATTACGTGTCGATGATGTGCAACGAGCACGCCTACGTGCGCGCGATCGAGACCCTGATGGGCATCGAGGCGCCGGAGCGTGCGCAGTACATCCGCACCATGTACGACGAAATCACCCGCATCCTCAACCACCTGATGTGGCTTGGCTCCAATGCGCTCGATCTGGGTGCGATGGCGGTGATGCTGTACGCCTTCCGCGAGCGCGAAGAGCTGATGGACTGCTATGAAGCGGTCTCCGGCGCGCGCATGCACGCGGCGTACTACCGTCCGGGCGGTGTCTACCGCGACCTGCCGGACCACATGCCGAAGTACAAG

*ppsA allel 93*

ATGACCGTGCGCGGCGTAAGAGCTGCGCAACACCTTCTCGATCAGCGACGAGGACGTGCAGGAACTGTCCAAGCAGGCGCTGGTCATCGAAAAGCACTACGGCCGCCCGATGGACATCGAGTGGGCCAAGGACGGTGTCAGCGGTAAGCTGTTCATCGTGCAGGCGCGCCCGGAAACGGTGAAGTCGCGCAGCCACGCCACCCAGATCGAGCGCTTCGCGCTGACCGAAAAGGGCGGCAACGTGCTGGCCGAAGGCCGTGCCGTCGGTGCCAAGATCGGTTCGGGCGTGGCCCGCGTGGTCAAGACGCTGGACGACATGAACCGCGTGCAGCCGGGCGACGTGCTGATCGCCGACATGACCGACCCGGACTGGGAACCGGTGATGAAGCGCGCTTCGGCCATCGTCACCAATCGCGGTGGCCGTACCTGCCACGCCGCGATCATCGCGCGCGAGCTGGGCGTGCCGGCCGTGGTCGGTTCGGGCAACGCCACCAAGGTCATCGAAGATGTCAACAGAGAGCAAKGGAAAAAA

*recA allel 74*

CGCGCCCTCGCTGCAGCTCTGGGCCAGATCGAAAAGCAGTTCGGCAAGGGCTCGGTGATGCGCATGGGCGACCGCGTGGTCGAGCCCGTCGAAGCCATCCCGACCGGTTCGCTGATGCTCGACATCGCACTGGGCATCGGCGGTCTGCCGAAGGGCCGTGTCGTCGAGATCTACGGGCCGGAATCCTCGGGCAAGACCACGCTGACCCTGCAGGCCATCGCCGAATGCCAGAAGATGGGCGGCACCGCGGCCTTCATCGACGCCGAGCACGCGCTGGACCCGATCTACGCCGCCAAGCTGGGCGTGAACGTGGACGACCTGCTGCTGTCGCAGCCGGATACCGGTGAGCAGGCGCTGGAAATCGCCGACATGCTGGTCCGTTCGGGTTCGGTCGACATCCTGGTGATCGACTCGGTCGCCGCGTTGACCCCGAAGGCTGAAATCGAAGGCGAGATGGGCGATCAGCTGCCGGGCCTGCAGGCCCGCCTGATGAGCCAGGCACTGCGCAAGCTGACCGGCAACATCAAGCGCTCCAACACCCTGGTGGTCTTCATCAACCAGCTGCGCATGAAGATCGGTGTGATGATGCCGGGCCAGAGCCCGGAAACCACCACCGGCGGCAACGCGCTGAAGTTCTATGCCTCGGTGCGCCTGGACATCCGCCGTATCGGCGCGATCAAGAAGGGCGACGAGATCATCGGCAACCAGACCAAGATCAAGGTCGTCAAGAACAAGCTGGCGCCTCCGTTCAA

**Strain no. 19**

*atpD allel 76*

GCGTCCTGCAGGAACGCGGCCGCGGCATCTCCGTGCCGGTCGGCGCTGGCACCCTGGGCCGCATCATGGACGTGCTCGGCCGTCCGATCGACGAAGCCGGCCCGGTGGCCGCCAGCGACAGCTGGGAAATCCACCGTGCGGCCCCGTCGTACGAAGACCAGTCCCCGGCCACCGAACTGCTGGAAACCGGCATCAAGGTCATCGACCTGATGTGCCCGTTCGCCAAGGGCGGCAAGGTCGGCCTGTTCGGCGGCGCCGGCGTCGGCAAGACCGTCAACATGATGGAACTGATCAACAACATCGCCAAGGCGCACAGCGGTCTGTCCGTGTTCGCCGGCGTGGGTGAGCGTACCCGTGAGGGCAACGACTTCTACCACGAAATGAAGGACTCCAACGTCCTGGACAAGGTGGCGATGGTGTACGGCCAGATGAACGAGCCGCCGGGCAACCGTCTGCGCGTCGCCCTGACCGGCCTGACCATGGCCGAGTACTTCCGCGACGAGAAGGACGAAAACGGCAAGGGCAAGGACGTTCTGCTGTTCGTCGACAACATCTCCGTGCCGGTCGGCGCTGGCACCCTGGGCCGCATCATGGACGTGCTCGGCCGTCCGATCGACGAAGCCGGCCCGGTGGCCGCCAGCGACAGCTGGGAAATCCACCGTGCGGCCCCGTCGTACGAAGACCAGTCCCCGGCCACCGAACTGCTGGAAACCGGCATCAAGGTCATCGACCTGATGTGCCCGTTCGCCAAGGGCGGCAAGGTCGGCCTGTTCGGCGGCGCCGGCGTCGGCAAGACCGTCAACATGATGGAACTGATCAACAACATCGCCAAGGCGCACAGCGGTCTGTCCGTGTTCGCCGGCGTGGGTGAGCGTACCCGTGAGGGCAACGACTTCTACCACGAAATGAAGGACTCCAACGTCCTGGACAAGGTGGCGATGGTGTACGGCCAGATGAACGAGCCGCCGGGCAACCGTCTGCGCGTCGCCCTGACCGGCCTGACCATGGCCGAGTACTTCCGCGACGAGAAGGACGAAAACGGCAAGGGCAAGGACGTTCTGCTGTTCGT

*gapA allel 68*

CGAACACCGAGGTGCAGGTCTCGCCGCGGAAATCGGTGGCCACGACCTTGTCTTCGGTGTAGCCGAGGATGCCCTTCAGCGGGCCTTCGCTCTGTGCCTTCACTTCAGCGCAGATCTCGGCGTAGGTGGCTTCCTTTTCCAGCTCGACGGTCAGGTCGACCACCGACACGTCCGAGGTCGGGACACGGAAGCTCATGCCGGTCAGCTTCTTGTTCAGTTCCGGGATGACCACGCCGACAGCCTTGGCCGCACCGGTGGACGACGGGATGATGTTCTCCAGGATGCCGCGGCCACCACGCCAGTCCTTGTTGGACGGGCCATCGACGGTCTTCTGGGTGGCAGTAGCCGCATGCACGGTGGTCATCAGGCCACGCTTGATGCCCCACTTGTCATTGATGACCTTGGCCAGCGGGGCCAGGCAGTTGGTGGTGCACGACGCGTTGGAAATGATGGCCTGGCCGGCGTAGGTCTTGTCGTTCACGCCGTAGACGAACATCGGCGTATCGTCCTTCGACGGGGCCGACATGATGACCTTCCTGGCGCCGGCATCGATGTGCTTCTGCGCGGTTTCCTTGGTCAGGAACAGGCCGGTGGACTCGATCACCACGTCCACGTCCACTTCGTTCCACTTCAGGTTGGCCGGGTCGCGTTCCTGGGTCAGGCGGATCTTCTTGCCGTTGACCAGCAGGTCGTTGCATCGTTGATGGCC

*guaA allel 7*

CGCTGGTACGGCGTGCAGTTCCACCCGGAAGTGACCCACACCCTGCAGGGCCAGGCGCTGCTGCGCCGCTTCGTGGTGGACGTGTGCGGCTGCCAGACCCTGTGGACCGCCGCCAACATCATCGACGACCAGATCGCCCGCGTGCGCGAACAGGTGGGCGATGACGAAGTGATCCTGGGCCTGTCCGGCGGCGTCGATTCGTCCGTGGTGGCCGCGCTGCTGCACAAGGCCATCGGCGAAAAGCTGACCTGCGTGTTCGTGGATACCGGCCTGCTGCGCTGGCAGGAAGGCGACCAGGTGATGGCGATGTTCGCCGAACACATGGGCGTGAAGGTCGTTCGCGTGAATGCCGCCGACCGTTACTTCGCCGCGCTGGAAGGCGTGAGCGACCCGGAAGCCAAGCGCAAGATCATCGGTAACCTGTTCGTTGAGATCTTCGATGAAGAGTCGAACAAGCTGAGCAACGCCAAGTGGCTGGCGCAGGGCACCATCTACCCGGACGTGATCGAGTCGGCTGGCAGCAAGACCGGCAAGGCGCATGTGATCAAGAGCCACCACAACGTGGGCGGCCTGCCAGAGCACATGAAGCTGGGCCTGGTGGAGCCGCTGCGCGAGCTGTTCAAGGACGAAGTGCGCCGCCTGGGTGTTGAACTGGGCCTGCCGCGCACCATGGTCT

*mutM allel 7*

CGCTCGGCACCTGCAGGGCCGCCGCGTGCATGGCGTGATCCTGCGCCGCGCCGACCTGCGCTGGCCGATTCCGCCGGAAGTGGCCGAGCTGCTGCCGGGGCAGCGCATCGAGGACATCCGCCGTCGCGCCAAGTACCTGCTGCTGGACACCGCCATCGGCAGCGCCGTGCTGCACCTGGGCATGTCCGGCAGCCTGCGTGTGCTGCCCGGCGATACCCCGCTGCGCGCGCACGACCATGTGGATATCAGCCTGGACAACGGCCGCCTGTTGCGCTTCAACGACCCGCGCCGTTTCGGCAGCCTGCTCTGGCAGCCGGCCGGCGAAGTCCACCCGCTGCTGCAGGGGCTGGGCCCGGAGCCGCTGGACGATGCCTTCGACGGGGACTACCTGTTCTCCCGCAGCCGTGGCCGCAGCGCGCCGGTGAAGACCTTCCTGATGGACCAGGCGGTGGTGGTGGGCGTGGGCAACATCACGCCGACGCCGGTATCGCCCTG

*nuoD allel 80*

AACCATGAACTTCGGCCCGCAGCATCCGGCCGCGCACGGTGTGCTGCGCCTGATCCTGGAGATGGACGGCGAAACCATCATGCGCGCCGACCCGCACGTGGGTCTGCTGCACCGTGGTACCGAAAAGCTGGCCGAGTCCAAGCCGTTCAACCAGTCGATCGGCTACATGGATCGCCTGGATTACGTGTCGATGATGTGCAACGAGCACGCCTACGTGCGCGCGATCGAGACCCTGATGGGCATCGAGGCGCCGGAGCGTGCGCAGTACATCCGCACCATGTACGACGAAATCACCCGCATCCTCAACCACCTGATGTGGCTTGGCTCCAATGCGCTCGATCTGGGTGCGATGGCGGTGATGCTGTACGCCTTCCGCGAGCGCGAAGAGCTGATGGACTGCTATGAAGCGGTCTCCGGCGCGCGCATGCACGCGGCGTACTACCGTCCGGGCGGTGTCTACCGCGACCTGCCGGACCACATGCCGAAGTACAAGAGGTCGCGCT

*ppsA allel 93*

TAAGAGCTGCGCAACACCTTCTCGATCAGCGACGAGGACGTGCAGGAACTGTCCAAGCAGGCGCTGGTCATCGAAAAGCACTACGGCCGCCCGATGGACATCGAGTGGGCCAAGGACGGTGTCAGCGGTAAGCTGTTCATCGTGCAGGCGCGCCCGGAAACGGTGAAGTCGCGCAGCCACGCCACCCAGATCGAGCGCTTCGCGCTGACCGAAAAGGGCGGCAACGTGCTGGCCGAAGGCCGTGCCGTCGGTGCCAAGATCGGTTCGGGCGTGGCCCGCGTGGTCAAGACGCTGGACGACATGAACCGCGTGCAGCCGGGCGACGTGCTGATCGCCGACATGACCGACCCGGACTGGGAACCGGTGATGAAGCGCGCTTCGGCCATCGTCACCAATCGCGGTGGCCGTACCTGCCACGCCGCGATCATCGCGCGCGAGCTGGGCGTGCCGGCCGTGGTCGGTTCGGGCAACGCCACCAAGGTCATCGAAGATGTCAACAGAGAGCAAAAAAAAACGACTA

*recA allel 74*

CAAGAAGCGCGCCCTCGCTGCAGCTCTGGGCCAGATCGAAAAGCAGTTCGGCAAGGGCTCGGTGATGCGCATGGGCGACCGCGTGGTCGAGCCCGTCGAAGCCATCCCGACCGGTTCGCTGATGCTCGACATCGCACTGGGCATCGGCGGTCTGCCGAAGGGCCGTGTCGTCGAGATCTACGGGCCGGAATCCTCGGGCAAGACCACGCTGACCCTGCAGGCCATCGCCGAATGCCAGAAGATGGGCGGCACCGCGGCCTTCATCGACGCCGAGCACGCGCTGGACCCGATCTACGCCGCCAAGCTGGGCGTGAACGTGGACGACCTGCTGCTGTCGCAGCCGGATACCGGTGAGCAGGCGCTGGAAATCGCCGACATGCTGGTCCGTTCGGGTTCGGTCGACATCCTGGTGATCGACTCGGTCGCCGCGTTGACCCCGAAGGCTGAAATCGAAGGCGAGATGGGCGATCAGCTGCCGGGCCTGCAGGCCCGCCTGATGAGCCAGGCACTGCGCAAGCTGACCGGCAACATCAAGCGCTCCAACACCCTGGTGGTCTTCATCAACCAGCTGCGCATGAAGATCGGTGTGATGATGCCGGGCCAGAGCCCGGAAACCACCACCGGCGGCAACGCGCTGAAGTTCTATGCCTCGGTGCGCCTGGACATCCGCCGTATCGGCGCGATCAAGAAGGGCGACGAGATCATCGGCAACCAGACCAAGATCAAGGTCGTCAAGAACAAGCTGGCGCCTCCGTTCAAG

**Strain no. 22**

*atpD allel 76*

GCAGGAACGCGGCCGCGGCATCTCCGTGCCGGTCGGCGCTGGCACCCTGGGCCGCATCATGGACGTGCTCGGCCGTCCGATCGACGAAGCCGGCCCGGTGGCCGCCAGCGACAGCTGGGAAATCCACCGTGCGGCCCCGTCGTACGAAGACCAGTCCCCGGCCACCGAACTGCTGGAAACCGGCATCAAGGTCATCGACCTGATGTGCCCGTTCGCCAAGGGCGGCAAGGTCGGCCTGTTCGGCGGCGCCGGCGTCGGCAAGACCGTCAACATGATGGAACTGATCAACAACATCGCCAAGGCGCACAGCGGTCTGTCCGTGTTCGCCGGCGTGGGTGAGCGTACCCGTGAGGGCAACGACTTCTACCACGAAATGAAGGACTCCAACGTCCTGGACAAGGTGGCGATGGTGTACGGCCAGATGAACGAGCCGCCGGGCAACCGTCTGCGCGTCGCCCTGACCGGCCTGACCATGGCCGAGTACTTCCGCGACGAGAAGGACGAAAACGGCAAGGGCAAGGACGTTCTGCTGTTCGTCGACAACATCTCCGTGCCGGTCGGCGCTGGCACCCTGGGCCGCATCATGGACGTGCTCGGCCGTCCGATCGACGAAGCCGGCCCGGTGGCCGCCAGCGACAGCTGGGAAATCCACCGTGCGGCCCCGTCGTACGAAGACCAGTCCCCGGCCACCGAACTGCTGGAAACCGGCATCAAGGTCATCGACCTGATGTGCCCGTTCGCCAAGGGCGGCAAGGTCGGCCTGTTCGGCGGCGCCGGCGTCGGCAAGACCGTCAACATGATGGAACTGATCAACAACATCGCCAAGGCGCACAGCGGTCTGTCCGTGTTCGCCGGCGTGGGTGAGCGTACCCGTGAGGGCAACGACTTCTACCACGAAATGAAGGACTCCAACGTCCTGGACAAGGTGGCGATGGTGTACGGCCAGATGAACGAGCCGCCGGGCAACCGTCTGCGCGTCGCCCTGACCGGCCTGACCATGGCCGAGTACTTCCGCGACGAGAAGGACGAAAACGGCAAGGGCAAGGACGTTCTGCTGTTCGT

*gapA allel 68*

CGATGCCGGCCTCGGCGTCGAACACCGAGGTGCAGGTCTCGCCGCGGAAATCGGTGGCCACGACCTTGTCTTCGGTGTAGCCGAGGATGCCCTTCAGCGGGCCTTCGCTCTGTGCCTTCACTTCAGCGCAGATCTCGGCGTAGGTGGCTTCCTTTTCCAGCTCGACGGTCAGGTCGACCACCGACACGTCCGAGGTCGGGACACGGAAGCTCATGCCGGTCAGCTTCTTGTTCAGTTCCGGGATGACCACGCCGACAGCCTTGGCCGCACCGGTGGACGACGGGATGATGTTCTCCAGGATGCCGCGGCCACCACGCCAGTCCTTGTTGGACGGGCCATCGACGGTCTTCTGGGTGGCAGTAGCCGCATGCACGGTGGTCATCAGGCCACGCTTGATGCCCCACTTGTCATTGATGACCTTGGCCAGCGGGGCCAGGCAGTTGGTGGTGCACGACGCGTTGGAAATGATGGCCTGGCCGGCGTAGGTCTTGTCGTTCACGCCGTAGACGAACATCGGCGTATCGTCCTTCGACGGGGCCGACATGATGACCTTCCTGGCGCCGGCATCGATGTGCTTCTGCGCGGTTTCCTTGGTCAGGAACAGGCCGGTGGACTCGATCACCACGTCCACGTCCACTTCGTTCCACTTCAGGTTGGCCGGGTCGCGTTCCTGGGTCAGGCGGATCTTCTTGCCGTTGACCAGCAGGTCGTTGCCCTGCACCGCCACGTCGGCCTTGAAACGACCGTGCACGGAGTCGTACTTGAGCATGTACGCCAGATAGTCCGGCTCCAGCAGATCGTTGATGGC

*guaA allel 7*

GCGCTGGTACGGCGTGCAGTTCCACCCGGAAGTGACCCACACCCTGCAGGGCCAGGCGCTGCTGCGCCGCTTCGTGGTGGACGTGTGCGGCTGCCAGACCCTGTGGACCGCCGCCAACATCATCGACGACCAGATCGCCCGCGTGCGCGAACAGGTGGGCGATGACGAAGTGATCCTGGGCCTGTCCGGCGGCGTCGATTCGTCCGTGGTGGCCGCGCTGCTGCACAAGGCCATCGGCGAAAAGCTGACCTGCGTGTTCGTGGATACCGGCCTGCTGCGCTGGCAGGAAGGCGACCAGGTGATGGCGATGTTCGCCGAACACATGGGCGTGAAGGTCGTTCGCGTGAATGCCGCCGACCGTTACTTCGCCGCGCTGGAAGGCGTGAGCGACCCGGAAGCCAAGCGCAAGATCATCGGTAACCTGTTCGTTGAGATCTTCGATGAAGAGTCGAACAAGCTGAGCAACGCCAAGTGGCTGGCGCAGGGCACCATCTACCCGGACGTGATCGAGTCGGCTGGCAGCAAGACCGGCAAGGCGCATGTGATCAAGAGCCACCACAACGTGGGCGGCCTGCCAGAGCACATGAAGCTGGGCCTGGTGGAGCCGCTGCGCGAGCTGTTCAAGGACGAAGTGCGCCGCCTGGGTGTTGAACTGGGCCTGCCGCGCACCATGGTCTAC

*mutM allel 7*

TAACGTCCTGCGCTCGGCACCTGCAGGGCCGCCGCGTGCATGGCGTGATCCTGCGCCGCGCCGACCTGCGCTGGCCGATTCCGCCGGAAGTGGCCGAGCTGCTGCCGGGGCAGCGCATCGAGGACATCCGCCGTCGCGCCAAGTACCTGCTGCTGGACACCGCCATCGGCAGCGCCGTGCTGCACCTGGGCATGTCCGGCAGCCTGCGTGTGCTGCCCGGCGATACCCCGCTGCGCGCGCACGACCATGTGGATATCAGCCTGGACAACGGCCGCCTGTTGCGCTTCAACGACCCGCGCCGTTTCGGCAGCCTGCTCTGGCAGCCGGCCGGCGAAGTCCACCCGCTGCTGCAGGGGCTGGGCCCGGAGCCGCTGGACGATGCCTTCGACGGGGACTACCTGTTCTCCCGCAGCCGTGGCCGCAGCGCGCCGGTGAAGACCTTCCTGATGGACCAGGCGGTGGTGGTGGGCGTGGGCAACATCACGCCGACGCCGGTATCGCCCTG

*nuoD allel 80*

CTAACCATGAACTTCGGCCCGCAGCATCCGGCCGCGCACGGTGTGCTGCGCCTGATCCTGGAGATGGACGGCGAAACCATCATGCGCGCCGACCCGCACGTGGGTCTGCTGCACCGTGGTACCGAAAAGCTGGCCGAGTCCAAGCCGTTCAACCAGTCGATCGGCTACATGGATCGCCTGGATTACGTGTCGATGATGTGCAACGAGCACGCCTACGTGCGCGCGATCGAGACCCTGATGGGCATCGAGGCGCCGGAGCGTGCGCAGTACATCCGCACCATGTACGACGAAATCACCCGCATCCTCAACCACCTGATGTGGCTTGGCTCCAATGCGCTCGATCTGGGTGCGATGGCGGTGATGCTGTACGCCTTCCGCGAGCGCGAAGAGCTGATGGACTGCTATGAAGCGGTCTCCGGCGCGCGCATGCACGCGGCGTACTACCGTCCGGGCGGTGTCTACCGCGACCTGCCGGACCACATGCCGAAGTACAAGA

*ppsA allel 93*

TCCTTCTCGATCAGCGACGAGGACGTGCAGGAACTGTCCAAGCAGGCGCTGGTCATCGAAAAGCACTACGGCCGCCCGATGGACATCGAGTGGGCCAAGGACGGTGTCAGCGGTAAGCTGTTCATCGTGCAGGCGCGCCCGGAAACGGTGAAGTCGCGCAGCCACGCCACCCAGATCGAGCGCTTCGCGCTGACCGAAAAGGGCGGCAACGTGCTGGCCGAAGGCCGTGCCGTCGGTGCCAAGATCGGTTCGGGCGTGGCCCGCGTGGTCAAGACGCTGGACGACATGAACCGCGTGCAGCCGGGCGACGTGCTGATCGCCGACATGACCGACCCGGACTGGGAACCGGTGATGAAGCGCGCTTCGGCCATCGTCACCAATCGCGGTGGCCGTACCTGCCACGCCGCGATCATCGCGCGCGAGCTGGGCGTGCCGGCCGTGGTCGGTTCGGGCAACGCCACCAAGGTCATCGAAGATGTCAACAGAGAGCAAGGAAAAAAACGACTAAGA

*recA allel 74*

GCGCGCCCTCGCTGCAGCTCTGGGCCAGATCGAAAAGCAGTTCGGCAAGGGCTCGGTGATGCGCATGGGCGACCGCGTGGTCGAGCCCGTCGAAGCCATCCCGACCGGTTCGCTGATGCTCGACATCGCACTGGGCATCGGCGGTCTGCCGAAGGGCCGTGTCGTCGAGATCTACGGGCCGGAATCCTCGGGCAAGACCACGCTGACCCTGCAGGCCATCGCCGAATGCCAGAAGATGGGCGGCACCGCGGCCTTCATCGACGCCGAGCACGCGCTGGACCCGATCTACGCCGCCAAGCTGGGCGTGAACGTGGACGACCTGCTGCTGTCGCAGCCGGATACCGGTGAGCAGGCGCTGGAAATCGCCGACATGCTGGTCCGTTCGGGTTCGGTCGACATCCTGGTGATCGACTCGGTCGCCGCGTTGACCCCGAAGGCTGAAATCGAAGGCGAGATGGGCGATCAGCTGCCGGGCCTGCAGGCCCGCCTGATGAGCCAGGCACTGCGCAAGCTGACCGGCAACATCAAGCGCTCCAACACCCTGGTGGTCTTCATCAACCAGCTGCGCATGAAGATCGGTGTGATGATGCCGGGCCAGAGCCCGGAAACCACCACCGGCGGCAACGCGCTGAAGTTCTATGCCTCGGTGCGCCTGGACATCCGCCGTATCGGCGCGATCAAGAAGGGCGACGAGATCATCGGCAACCAGACCAAGATCAAGGTCGTCAAGAACAAGCTGGCGCCTCCGTTCAAGCAG

**Strain no. 25 ST142**

*atpD allel 80*

GACGGGSSYGCCTCTACCGCTACACCCTGGCCGGTACCGAAGTGTCGGCACTGCTGGGCCGTATGCCGTCCGCCGTGGGTTACCAGCCGACCCTGGCCGAGGAAATGGGCGTCCTGCAGGACGGCATCTCGGTGCCGGTCGGCGCTGGCACCCTGGGCCGCATCATGGACGTGCTCGGCCGTCCGATCGACGAAGCCGGCCCGGTGGCCGCCAGCGACAGCTGGGAAATCCACCGTGCGGCCCCGTCGTACGAAGACCAGTCCCCGGCTACCGAGCTGCTGGAAACCGGCATCAAGGTCATCGACCTGATGTGCCCGTTCGCCAAGGGCGGCAAGGTCGGCCTGTTCGGCGGCGCCGGCGTCGGCAAGACCGTCAACATGATGGAGCTGATCAACAACATCGCCAAGGCGCACAGCGGTCTGTCCGTGTTCGCCGGCGTGGGTGAGCGTACCCGTGAGGGCAACGACTTCTACCACGAAATGAAGGACTCCAACGTCCTCGACAAGGTGGCGATGGTGTACGGCCAGATGAACGAGCCGCCGGGCAACCGTCTGCGCGTCGCCCTGACCGGCCTGACCATGGCCGAGTACTTCCGCGACGAGAAGGACGAAAACGGCAAGGGCAAGGACGTCCTGCTGTTCGTCGACAA

*gapA allel 89*

AGGTTGGTATGATTACCTGGCGTACATGCTCAAGTACGACTCCGTGCACGGCCGCTTCAAGGCCGACGTGGCCGTGCAGGGCAACGACCTGCTGGTGAACGGCAAGAAGATCCGCCTGACCCAGGAACGCGACCCGGCCAACCTGAAGTGGGACGAAGTCGGCGCCGACGTGGTGCTGGAAGCCACCGGCCTGTTCCTGACCAAGGAAACCGCGCAGAAGCACATCGATGCCGGCGCGAAGAAGGTCATCATGTCGGCCCCGTCGAAGGACGACACGCCGATGTTCGTGTTCGGCGTGAACGACAAGACCTACGCCGGCCAGGCCATCATTTCCAACGCGTCGTGCACCACCAACTGCCTGGCCCCGCTGGCCAAGGTCATCAACGACAAGTGGGGCATCAAGCGTGGCCTGATGACCACGGTGCATGCGGCCACCGCCACCCAGAAGACCGTCGATGGCCCGTCCAACAAGGACTGGCGCGGCGGCCGTGGCATCCTGGAAAACATCATTCCGTCGTCCACCGGTGCGGCAAAGGCCGTCGGCGTGGTCATTCCGGAACTGAACAAGCGCTGAAGTGAAGGCACAGAGCG

*guaA allel 43*

CGCTGGTACGGCGTGCAGCTTCGTGGTCGACGTGTGCGGCTGCCAGACCCTGTGGACCGCCGCCAACATCATCGACGACCAGATCGCCCGCGTGCGCGAACAGGTGGGCGATGACGAAGTGATCCTGGGCCTGTCCGGCGGCGTCGATTCGTCCGTGGTGGCCGCACTGCTGCACAAGGCCATCGGCGAAAAGCTGACCTGCGTGTTCGTGGATACCGGCCTGCTGCGCTGGCAGGAAGGCGACCAGGTGATGGCGATGTTCGCCGAGCACATGGGCGTAAAGGTGGTGCGCGTAAATGCCGCCGACCGTTACTTCGCCGCGCTGGAAGGCGTGAGCGACCCGGAAGCCAAGCGCAAGATCATCGGCAACCTGTTCGTTGAGATCTTCGACGAAGAGTCGAACAGGCTGAAGAACGCCAAATGGCTGGCGCAGGGCACCATCTACCCGGACGTGATCGAGTCGGCCGGCAGCAAGACCGGCAAGGCGCATGTGATCAAGAGCCACCACAACGTGGGCGGCCTGCCGGAGCACATGAAGCTGGGCCTGGTGGAGCCCTGGGTGTTGAACTGGGCCTGCCGCGCACCATGGTC

*mutM allel 73*

AACTGCCCGAAGTCGAAACCACCTGCAGGGTCGTCGCGTGCATGGCGTGATCCTGCGCCGCGCCGACCTGCGCTGGCCGATTCCGCCGGAAGTGGCCGAGCTGCTGCCGGGCCAGCGCATCGAGGAGATCCGTCGCCGCGCCAAGTACCTGCTGCTGGACACCGCCGTCGGCAGCGCGGTGCTGCACCTGGGCATGTCCGGCAGCCTGCGCGTGCTGCCTGGCGATACCCCGCTGCGCGCCCACGACCACGTCGACATCAGCCTGGACAACGGCCGCCTGCTGCGCTTCAACGACCCGCGCCGCTTCGGCAGCCTGCTCTGGCAGCCGGCCGGCGAAGTCCACCCGCTGCTGCAGGGGCTGGGCCCGGAACCGCTGGACGACGTGTTTGACGGCGACTACCTGTTCGCCCGCAGCCGGGGCCGCAGTGCGCCGGTGAAGACCTTCCTGATGGACCAGGCGGTGGTGGTGGGCGTCGGCAACATCGAGGATCTCCTTCACCGCATC

*nuoD allel 72*

TAACCATGAACTTCGGCCCGCAGCATCCGGCCGCTCACGGTGTGCTGCGCCTGATCCTGGAAATGGACGGTGAAACCATCATGCGCGCCGACCCGCACGTGGGTCTGCTGCACCGTGGTACCGAAAAGCTGGCCGAGTCCAAGCCGTTCAACCAGTCGATCGGCTACATGGATCGCCTGGATTACGTGTCGATGATGTGCAACGAGCACGCCTACGTGCGCGCGATCGAGACCCTGATGGGCATCGAGGCGCCGGAGCGTGCCCAGTACATCCGCACCATGTACGATGAAATCACCCGCATCCTCAACCACCTGATGTGGCTGGGCTCCAACGCGCTCGACCTGGGCGCGATGGCGGTGATGCTGTACGCCTTCCGCGAACGCGAAGAGCTGATGGACTGCTACGAAGCGGTTTCCGGCGCGCGCATGCACGCGGCGTACTACCGTCCGGGCGGTGTCTACCGCGACCTGCCGGACCACATGCCGAAGTACAAGA

*ppsA allel 98*

AAGTTACTCCTGGCCGAACTCCGCACCACCTTCTCGATCAGCGACGAGGACGTGCAGGAACTGGCCAAGCAGGCGCTGGTCATCGAAAAGCACTACGGCCGCCCGATGGACATCGAGTGGGCCAAGGACGGTGTCAGCGGCAAGCTGTTCATCGTGCAGGCGCGCCCGGAAACGGTGAAGTCGCGCAGCCACGCCACCCAGATCGAGCGCTTCGCGCTGACCGAGAAGGGCGGCAAGGTACTGGCCGAGGGCCGTGCCGTCGGCGCCAAGATCGGTTCGGGCGTGGCCCGCGTGGTGAAGACGCTGGACGACATGAACCGCGTGCAGCCGGGCGACGTGCTGATCGCCGACATGACCGATCCCGATTGGGAACCGGTGATGAAGCGCGCCTCGGCCATCGTCACCAACCGCGGTGGCCGCACCTGCCACGCCGCGATCATCGCGCGCGAGCTGGGCGTGCCAGCCGTGGTCGGTTCGGGCAACGCCACCCAGGTCATCGAGGATGGTCGGGAGGAGGGGAAGCGGCTWCSCCGACTRAGCCGGARTGAGCMCAACAASMSCGCGCTAAKMGKATCTGSCCTGCMCGAAGAGAAGTCGGGCC

*recA allel 79*

AAGAAGCGCGCCTTGGCTGCTGCTCTGGGCCAGATCGAGAAGCAGTTCGGCAAGGGCTCGGTGATGCGCATGGGCGACCGCGTGGTCGAGCCCGTCGAAGCCATCCCGACCGGTTCGCTGATGCTCGACATCGCACTGGGCATTGGCGGTCTGCCGAAGGGCCGTGTCGTTGAGATCTACGGGCCGGAATCCTCGGGCAAGACCACGCTGACCCTGCAGGCCATCGCCGAATGCCAGAAGATGGGTGGCACCGCCGCCTTCATCGATGCCGAGCACGCGCTGGACCCGATCTATGCCGCCAAGCTGGGCGTGAACGTGGACGACCTGCTGCTGTCGCAGCCGGATACCGGTGAGCAGGCGCTGGAAATCGCCGACATGCTGGTCCGTTCGGGCTCGGTCGACATCCTGGTGGTCGACTCGGTCGCCGCACTGACCCCGAAGGCCGAAATCGAAGGCGAGATGGGTGACCAGCTGCCGGGTCTGCAGGCCCGCCTGATGAGCCAGGCGCTGCGCAAGTTGACCGGCAACATCAAGCGCTCCAACACCCTGGTGATCTTCATCAACCAGCTGCGCATGAAGATCGGCGTGATGATGCCGGGCCAGAGCCCGGAAACCACCACGGGCGGCAACGCGCTGAAGTTCTATGCCTCGGTCCGCCTGGACATCCGCCGTATCGGCGCGATCAAGAAGGGTGACGAGATCATCGGCAACCAGACCAAGATCAAGGTGGTCAAGAACAAGCTGGCGCCTCCGTTCAAGC

**Strain no. 129**

*atpD allel 76*

GAACGCGGCCGCGGCATCTCCGTGCCGGTCGGCGCTGGCACCCTGGGCCGCATCATGGACGTGCTCGGCCGTCCGATCGACGAAGCCGGCCCGGTGGCCGCCAGCGACAGCTGGGAAATCCACCGTGCGGCCCCGTCGTACGAAGACCAGTCCCCGGCCACCGAACTGCTGGAAACCGGCATCAAGGTCATCGACCTGATGTGCCCGTTCGCCAAGGGCGGCAAGGTCGGCCTGTTCGGCGGCGCCGGCGTCGGCAAGACCGTCAACATGATGGAACTGATCAACAACATCGCCAAGGCGCACAGCGGTCTGTCCGTGTTCGCCGGCGTGGGTGAGCGTACCCGTGAGGGCAACGACTTCTACCACGAAATGAAGGACTCCAACGTCCTGGACAAGGTGGCGATGGTGTACGGCCAGATGAACGAGCCGCCGGGCAACCGTCTGCGCGTCGCCCTGACCGGCCTGACCATGGCCGAGTACTTCCGCGACGAGAAGGACGAAAACGGCAAGGGCAAGGACGTTCTGCTGTTCGTCGACAACATCTCCGTGCCGGTCGGCGCTGGCACCCTGGGCCGCATCATGGACGTGCTCGGCCGTCCGATCGACGAAGCCGGCCCGGTGGCCGCCAGCGACAGCTGGGAAATCCACCGTGCGGCCCCGTCGTACGAAGACCAGTCCCCGGCCACCGAACTGCTGGAAACCGGCATCAAGGTCATCGACCTGATGTGCCCGTTCGCCAAGGGCGGCAAGGTCGGCCTGTTCGGCGGCGCCGGCGTCGGCAAGACCGTCAACATGATGGAACTGATCAACAACATCGCCAAGGCGCACAGCGGTCTGTCCGTGTTCGCCGGCGTGGGTGAGCGTACCCGTGAGGGCAACGACTTCTACCACGAAATGAAGGACTCCAACGTCCTGGACAAGGTGGCGATGGTGTACGGCCAGATGAACGAGCCGCCGGGCAACCGTCTGCGCGTCGCCCTGACCGGCCTGACCATGGCCGAGTACTTCCGCGACGAGAAGGACGAAAACGGCAAGGGCAAGGACGTTC

*gapA allel 68*

AGGTTGGTATCAACGGTTTCGGTCGCATCGGGCGTAACGTCCTGCGCTCGGCGGTGCTGAACTTCGGCGACGACATCGAAATCGTGGCCATCAACGATCTGCTGGAGCCGGACTATCTGGCGTACATGCTCAAGTACGACTCCGTGCACGGTCGTTTCAAGGCCGACGTGGCGGTGCAGGGCAACGACCTGCTGGTCAACGGCAAGAAGATCCGCCTGACCCAGGAACGCGACCCGGCCAACCTGAAGTGGAACGAAGTGGACGTGGACGTGGTGATCGAGTCCACCGGCCTGTTCCTGACCAAGGAAACCGCGCAGAAGCACATCGATGCCGGCGCCAGGAAGGTCATCATGTCGGCCCCGTCGAAGGACGATACGCCGATGTTCGTCTACGGCGTGAACGACAAGACCTACGCCGGCCAGGCCATCATTTCCAACGCGTCGTGCACCACCAACTGCCTGGCCCCGCTGGCCAAGGTCATCAATGACAAGTGGGGCATCAAGCGTGGCCTGATGACCACCGTGCATGCGGCTACTGCCACCCAGAAGACCGTCGATGGCCCGTCCAACAAGGACTGGCGTGGTGGCCGCGGCATCCTGGAGAACATCATCCCGTCGTCCACCGGTGCGGCCAAGGCTGTCGGCGTGGTCATCCCGGAACTGAACAAGAAGCTGACCGGCATGAGCTTCCGTGTCCCGACCTCGGACGTGTCGGTGGTCGACCTGACCGTCGAGCTGGAAAAGGAAGCCACCTACGCCGAGATCTGCGCTGAAGTGAAGGCACAGAGCG

*guaA allel 105*

GCCGGATTCCGGAGTGACCACACCCTGCTGCTGCGCCGCTTCGTGGTGGATGTGTGCGGCTGCCAGACCCTGTGGACCGCCGCCAACATCATCGACGACCAGATCGCCCGCGTGCGCGAACAGGTGGGCGATGACGAAGTGATCCTGGGCCTGTCCGGCGGCGTCGATTCGTCCGTGGTGGCTGCGCTGCTGCACAAGGCCATCGGCGAGAAGCTGACCTGCGTGTTCGTGGATACCGGCCTGCTGCGCTGGCAGGAAGGCGACCAGGTGATGGCGATGTTCGCCGAGCACATGGGCGTGAAGGTGGTGCGCGTGAATGCCGCCGACCGTTACTTCAGCGCGCTGGAAGGCGTGAGCGACCCGGAAGCCAAGCGCAAGATCATCGGCAACCTGTTCGTTGAGATCTTCGACGAAGAGTCGAACAAGCTGAAGAACGCCAAGTGGCTGGCGCAGGGCACCATCTACCCGGACGTGATCGAGTCGGCCGGCAGCAAGACCGGCAAGGCGCATGTGATCAAGAGCCACCACAACGTGGGCGGCCTGCCGGAGCACATGAAGCTGGGCCTGGTGGAGCCGCAAGGCGCATGTGATCAAGAGCCACCACAACGTGGGCGGCCTGCCAGAGCACATGAAGCTGGGCCTGGTGGAGCCGCTGCGCGAGCTGTTCAAGGACGAAGTGCGCCGCCTGGGTGTTGAACTGGGCCTGCCGCGCACCATGGTCTCCGGCCCATTCMGAAA

*mutM allel 67*

AACTGCCCGAAGTCGAAAACCTGCAGGGTCGCCGCGTACACGGCGTGATCCTGCGCCGCGCCGATCTGCGCTGGCCGATTCCGCCGGAAGTGGCCGAGCTGCTGCCGGGGCAGCGCATCGAGGAGATCCGTCGCCGCGCCAAGTACCTGCTGCTGGATACCGCCATCGGCAGCGCGGTGCTGCACCTGGGCATGTCCGGCAGCCTCCGCGTGCTGCCTGGCGATACCCCGCTGCGCGCCCACGACCACGTCGACATCAGCCTGGACAACGGCCGCCTGCTGCGCTTCAACGACCCGCGCCGCTTCGGCAGCCTGCTGTGGCAGCCGGCCGGTGAGATCCATCCGCTGCTGCAGGGGCTGGGCCCGGAGCCGCTGGATGATGCCTTCGACGGGGACTACCTGTTCGCCCGCAGCCGCGGCCGCAGCGCGCCGGTGAAGACCTTCCTGATGGACCAGGCGGTGGTGGTGGGCGTGGGCATCTCCTTCACCGCATC

*nuoD allel 80*

ACCATGAACTTCGGCCCGCAGCATCCGGCCGCGCACGGTGTGCTGCGCCTGATCCTGGAGATGGACGGCGAAACCATCATGCGCGCCGACCCGCACGTGGGTCTGCTGCACCGTGGTACCGAAAAGCTGGCCGAGTCCAAGCCGTTCAACCAGTCGATCGGCTACATGGATCGCCTGGATTACGTGTCGATGATGTGCAACGAGCACGCCTACGTGCGCGCGATCGAGACCCTGATGGGCATCGAGGCGCCGGAGCGTGCGCAGTACATCCGCACCATGTACGACGAAATCACCCGCATCCTCAACCACCTGATGTGGCTTGGCTCCAATGCGCTCGATCTGGGTGCGATGGCGGTGATGCTGTACGCCTTCCGCGAGCGCGAAGAGCTGATGGACTGCTATGAAGCGGTCTCCGGCGCGCGCATGCACGCGGCGTACTACCGTCCGGGCGGTGTCTACCGCGACCTGCCGGACCACATGCCGAAGTACAAGA

*ppsA allel 93*

GTTACATGACCGTGCGCGGCGTAAGAGCTGCGCAACACCTTCTCGATCAGCGACGAGGACGTGCAGGAACTGTCCAAGCAGGCGCTGGTCATCGAAAAGCACTACGGCCGCCCGATGGACATCGAGTGGGCCAAGGACGGTGTCAGCGGTAAGCTGTTCATCGTGCAGGCGCGCCCGGAAACGGTGAAGTCGCGCAGCCACGCCACCCAGATCGAGCGCTTCGCGCTGACCGAAAAGGGCGGCAACGTGCTGGCCGAAGGCCGTGCCGTCGGTGCCAAGATCGGTTCGGGCGTGGCCCGCGTGGTCAAGACGCTGGACGACATGAACCGCGTGCAGCCGGGCGACGTGCTGATCGCCGACATGACCGACCCGGACTGGGAACCGGTGATGAAGCGCGCTTCGGCCATCGTCACCAATCGCGGTGGCCGTACCTGCCACGCCGCGATCATCGCGCGCGAGCTGGGCGTGCCGGCCGTGGTCGGTTCGGGCAACGCCACCAAGGTCATCGAAGATGTCAACAGAGAGCA

*recA allel 74*

GCGCGCCCTCGCTGCAGCTCTGGGCCAGATCGAAAAGCAGTTCGGCAAGGGCTCGGTGATGCGCATGGGCGACCGCGTGGTCGAGCCCGTCGAAGCCATCCCGACCGGTTCGCTGATGCTCGACATCGCACTGGGCATCGGCGGTCTGCCGAAGGGCCGTGTCGTCGAGATCTACGGGCCGGAATCCTCGGGCAAGACCACGCTGACCCTGCAGGCCATCGCCGAATGCCAGAAGATGGGCGGCACCGCGGCCTTCATCGACGCCGAGCACGCGCTGGACCCGATCTACGCCGCCAAGCTGGGCGTGAACGTGGACGACCTGCTGCTGTCGCAGCCGGATACCGGTGAGCAGGCGCTGGAAATCGCCGACATGCTGGTCCGTTCGGGTTCGGTCGACATCCTGGTGATCGACTCGGTCGCCGCGTTGACCCCGAAGGCTGAAATCGAAGGCGAGATGGGCGATCAGCTGCCGGGCCTGCAGGCCCGCCTGATGAGCCAGGCACTGCGCAAGCTGACCGGCAACATCAAGCGCTCCAACACCCTGGTGGTCTTCATCAACCAGCTGCGCATGAAGATCGGTGTGATGATGCCGGGCCAGAGCCCGGAAACCACCACCGGCGGCAACGCGCTGAAGTTCTATGCCTCGGTGCGCCTGGACATCCGCCGTATCGGCGCGATCAAGAAGGGCGACGAGATCATCGGCAACCAGACCAAGATCAAGGTCGTCAAGAACAAGCTGGCGCCTCCGTTC

**Strain no. 318 ST116**

*atpD allel 3*

TCGGCGCTGGCACCCTGGGCCGCAGGCATCTCGGTGCCGGTCGGCGCCGGCACCCTGGGCCGCATCATGGACGTGCTCGGTCGTCCGATCGACGAAGCCGGCCCGGTGGCCGCCAGCGACAGCTGGGAAATCCACCGTGCGGCCCCGTCGTACGAAGACCAGTCCCCGGCCACCGAACTGCTGGAAACCGGCATCAAGGTCATCGACCTGATGTGCCCGTTCGCCAAGGGCGGCAAGGTCGGCCTGTTCGGCGGCGCCGGCGTCGGCAAGACCGTCAACATGATGGAGCTGATCAACAACATCGCCAAGGCGCACAGCGGTCTGTCCGTGTTCGCCGGCGTGGGTGAGCGTACCCGTGAGGGCAACGACTTCTACCACGAAATGAAGGACTCCAACGTCCTCGACAAGGTGGCGATGGTGTACGGCCAGATGAACGAGCCGCCGGGCAACCGTCTGCGCGTCGCCCTGACCGGCCTGACCATGGCCGAGTACTTCCGCGACGAGAAGGACGAAAACGGCAAGGGCAAGGACGTCCTGCTGT

*gapA allel 1*

TTCGTAACTACCTGGCGTACATGCTCAAGTACGACTCCGTGCACGGCCGCTTCAAGGCCGACGTGGCAGTGCAGGGCAACGACCTGCTGGTCAACGGCAAGAAGATCCGCCTGACCCAGGAACGCGACCCGGCCAACCTGAGACTACCTGGCGTACATGCTCAAGTACGACTCCGTGCACGGCCGCTTCAAGGCCGACGTGGCAGTGCAGGGCAACGACCTGCTGGTCAACGGCAAGAAGATCCGCCTGACCCAGGAACGCGACCCGGCCAACCTGAAGTGGGATGAAGTCGGTGCCGACGTGGTGCTGGAAGCCACCGGCCTGTTCCTGACCAAGGAAACCGCGCAGAAGCACATCGATGCCGGCGCCAAGAAGGTCATCATGTCGGCGCCGTCGAAGGACGACACGCCGATGTTCGTCTACGGCGTGAACGACAAGACCTACGCCGGCCAGGCGATCATTTCCAACGCCTCGTGCACCACCAACTGCCTGGCCCCGCTGGCCAAGGTCATCAATGACAAGTGGGGCATCAAGCGCGGCCTGATGACCACCGTGCATGCGGCAACCGCCACCCAGAAGACCGTCGATGGCCCGTCCAACAAGGACTGGCGCGGTGGCCGTGGCATCCTGGAGAACATCATCCCGTCGTCCACCGGTGCGGCCAAGGCCGTCGGCGTGGTCATCCCGGAACTGAACAAG

*guaA allel 84*

CGCTGGTACGGCGTGCAGTTCCACCCGGAAGTGACCCACACCCTGCAGGGCCAGGCGCTGCTGCGCCGCTTCGTGGTGGATGTGTGCGGCTGCCAGACCCTGTGGACCGCCGCCAACATCATCGACGACCAGATCGCCCGCGTGCGCGAACAGGTGGGCGATGACGAAGTGATCCTGGGCCTGTCCGGTGGCGTCGATTCGTCCGTGGTGGCCGCGCTGCTGCACAAGGCCATCGGCGAGAAGCTGACCTGCGTGTTCGTGGATACCGGCCTGCTGCGCTGGCAGGAAGGCGACCAGGTGATGGCGATGTTTGCCGAGCACATGGGCGTGAAGGTCGTGCGTGTGAATGCCGCCGACCGTTACTTCGCCGCCCTGGAAGGCGTGAGCGACCCGGAAGCCAAGCGCAAGATCATCGGCAACCTGTTCGTTGAGATCTTCGACGAAGAGTCGAACAAGCTGAAGAACGCCAAGTGGCTGGCGCAGGGCACCATCTACCCGGACGTGATCGAGTCGGCCGGCAGCAAGACCGGCAAGGCGCATGTGATCAAGAGTCACCACAACGTGGGCGGCCTGCCGGAGCACATGAAGCTGGGCCTGGTGGAGCCGCTGCGCGAGCTGTTCAAGGACGAAGTGCGCCGCCTGGGCGTCGAGCTGGGCCTGCCGCGCGCCATGGTCT

*mutM allel 58*

AACTGCCACCTGCAGGGCCGCCGCGTGCATGGCGTGATCCTGCGCCGCGCCGACCTGCGCTGGCCGATTCCGACGGAAGTGGCCGAGCTGCTGCCGGGGCAGCGCATCGAGGACATCCGCCGTCGCGCCAAGTACCTGCTGCTGGACACCGCCATCGGCAGCGCCGTGCTGCACCTGGGCATGTCCGGCAGCCTGCGTGTGCTGCCCGGCGATACCCCGCTGCGCGCGCACGACCATGTGGATATCAGCCTGGACAACGGCCGCCTGTTGCGCTTCAACGACCCGCGCCGTTTCGGCAGCCTGCTCTGGCAGCCGGCCGGCGAAGTCCACCCGCTGCTGCAGGGGCTGGGCCCGGAGCCGCTGGACGATGCCTTCGACGGGGACTACCTGTTCGCCCGCAGCCGTGGCCGCAGCGCGCCGGTGAAGACCTTCCTGATGGACCAGGCGGTGGTGGTGGGCGTGGGCAACATCTC

*nuoD allel 25*

AACCATGAACTTCGGCCCGCAGCATCCGGCCGCTCACGGTGTGCTGCGCCTGATCCTGGAAATGGACGGTGAAACCATCATGCGCGCCGACCCGCACGTGGGTCTGCTGCACCGTGGTACCGAAAAGCTGGCCGAGTCCAAGCCGTTCAACCAGTCGATCGGTTACATGGATCGACTGGATTACGTGTCGATGATGTGCAACGAGCACGCCTACGTGCGCGCGATCGAGACCCTGATGGGCATCGAGGCGCCGGAGCGTGCGCAGTACATCCGCACCATGTACGACGAGATCACCCGCATCCTCAACCACCTGATGTGGCTGGGCTCCAACGCGCTCGACCTGGGTGCGATGGCGGTGATGCTGTACGCCTTCCGCGAGCGCGAAGAGCTGATGGATTGCTATGAAGCAGTCTCCGGCGCACGCATGCACGCGGCGTACTACCGTCCGGGCGGTGTCTACCGCGACCTGCCGGACCACATGCCGAAGTACAAGGGGTCGCGCTA

*ppsA allel 82*

AAGGTCGAACTGCGCAACACCTTCTCGATCAGCGACGAGGACGTGCAGGAACTGTCCAAGCAGGCGCTGGTCATCGAAAAGCACTACGGCCGCCCGATGGACATTGAGTGGGCCAAGGACGGTGTCAGCGGCAAGCTGTTCATCGTGCAGGCGCGCCCGGAGACGGTGAAGTCGCGCAGCCACGCCACCCAGATCGAGCGCTTCGCGCTGACCGAAAAGGGCGGCAACGTGCTGGCCGAAGGCCGCGCCGTGGGCGCCAAGATCGGTTCGGGCGTGGCCCGCGTGGTCAAGACGCTGGACGACATGAACCGCGTGCAGCCGGGCGACGTGCTGATCGCCGACATGACCGACCCGGACTGGGAACCGGTGATGAAGCGCGCTTCGGCCATCGTCACCAACCGTGGTGGCCGCACCTGCCACGCCGCGATCATCGCGCGCGAGCTGGGCGTGCCCGCCGTGGTCGGTTCGGGCAACGCCACCAAGGTCATTGAAGATGGCC

*recA allel 6*

GAAGCGCGCCCTCGCTGCAGCTCTGGGCCAGATCGAAAAGCAGTTCGGCAAGGGCTCGGTGATGCGCATGGGCGACCGCGTGGTCGAACCCGTCGAAGCCATCCCGACCGGTTCGCTGATGCTCGACATCGCGCTGGGCATTGGTGGTCTGCCGAAGGGCCGTGTCGTCGAGATCTACGGGCCGGAATCCTCGGGCAAGACCACCTTGACCCTGCAGGCCATCGCCGAATGCCAGAAGATGGGCGGCACCGCGGCCTTCATCGACGCCGAGCACGCGCTGGACCCGATCTACGCCGCCAAGCTGGGCGTGAACGTGGACGACCTGCTGCTGTCGCAGCCGGACACCGGTGAGCAGGCGCTGGAAATCGCCGACATGCTGGTCCGTTCGGGTTCGGTCGACATCCTGGTGATCGACTCGGTTGCCGCGCTGACCCCGAAGGCCGAAATCGAAGGCGAGATGGGCGACCAGCTGCCAGGCCTTCAGGCCCGCCTGATGAGCCAGGCGCTGCGCAAGCTGACCGGCAACATCAAGCGCTCCAACACCCTGGTGATCTTCATCAACCAGCTGCGCATGAAGATCGGCGTGATGATGCCGGGCCAGAGCCCGGAAACCACCACGGGTGGCAACGCGCTGAAGTTCTACGCCTCGGTCCGCCTGGACATCCGCCGTATCGGCGCGATCAAGAAGGGTGACGAGATCATCGGTAACCAGACCAAGATCAAGGTCGTCAAGAACAAGCTGGCACCTCCGTTCAAGA

**Strain no. 327**

*atpD allel 76*

CGCGGCCGCGGCATCTCCGTGCCGGTCGGCGCTGGCACCCTGGGCCGCATCATGGACGTGCTCGGCCGTCCGATCGACGAAGCCGGCCCGGTGGCCGCCAGCGACAGCTGGGAAATCCACCGTGCGGCCCCGTCGTACGAAGACCAGTCCCCGGCCACCGAACTGCTGGAAACCGGCATCAAGGTCATCGACCTGATGTGCCCGTTCGCCAAGGGCGGCAAGGTCGGCCTGTTCGGCGGCGCCGGCGTCGGCAAGACCGTCAACATGATGGAACTGATCAACAACATCGCCAAGGCGCACAGCGGTCTGTCCGTGTTCGCCGGCGTGGGTGAGCGTACCCGTGAGGGCAACGACTTCTACCACGAAATGAAGGACTCCAACGTCCTGGACAAGGTGGCGATGGTGTACGGCCAGATGAACGAGCCGCCGGGCAACCGTCTGCGCGTCGCCCTGACCGGCCTGACCATGGCCGAGTACTTCCGCGACGAGAAGGACGAAAACGGCAAGGGCAAGGACGTTCTGCTGTTCGTCGACAACATCTCCGTGCCGGTCGGCGCTGGCACCCTGGGCCGCATCATGGACGTGCTCGGCCGTCCGATCGACGAAGCCGGCCCGGTGGCCGCCAGCGACAGCTGGGAAATCCACCGTGCGGCCCCGTCGTACGAAGACCAGTCCCCGGCCACCGAACTGCTGGAAACCGGCATCAAGGTCATCGACCTGATGTGCCCGTTCGCCAAGGGCGGCAAGGTCGGCCTGTTCGGCGGCGCCGGCGTCGGCAAGACCGTCAACATGATGGAACTGATCAACAACATCGCCAAGGCGCACAGCGGTCTGTCCGTGTTCGCCGGCGTGGGTGAGCGTACCCGTGAGGGCAACGACTTCTACCACGAAATGAAGGACTCCAACGTCCTGGACAAGGTGGCGATGGTGTACGGCCAGATGAACGAGCCGCCGGGCAACCGTCTGCGCGTCGCCCTGACCGGCCTGACCATGGCCGAGTACTTCCGCGACGAGAAGGACGAAAACGGCAAGGGCAAGGACGTTCTGCTGTTCGT

*gapA allel 68*

CCGGCCTCGGCGTCGAACACCGAGGTGCAGGTCTCGCCGCGGAAATCGGTGGCCACGACCTTGTCTTCGGTGTAGCCGAGGATGCCCTTCAGCGGGCCTTCGCTCTGTGCCTTCACTTCAGCGCAGATCTCGGCGTAGGTGGCTTCCTTTTCCAGCTCGACGGTCAGGTCGACCACCGACACGTCCGAGGTCGGGACACGGAAGCTCATGCCGGTCAGCTTCTTGTTCAGTTCCGGGATGACCACGCCGACAGCCTTGGCCGCACCGGTGGACGACGGGATGATGTTCTCCAGGATGCCGCGGCCACCACGCCAGTCCTTGTTGGACGGGCCATCGACGGTCTTCTGGGTGGCAGTAGCCGCATGCACGGTGGTCATCAGGCCACGCTTGATGCCCCACTTGTCATTGATGACCTTGGCCAGCGGGGCCAGGCAGTTGGTGGTGCACGACGCGTTGGAAATGATGGCCTGGCCGGCGTAGGTCTTGTCGTTCACGCCGTAGACGAACATCGGCGTATCGTCCTTCGACGGGGCCGACATGATGACCTTCCTGGCGCCGGCATCGATGTGCTTCTGCGCGGTTTCCTTGGTCAGGAACAGGCCGGTGGACTCGATCACCACGTCCACGTCCACTTCGTTCCACTTCAGGTTGGCCGGGTCGCGTTCCTGGGTCAGGCGGATCTTCTTGCCGTTGACCAGCAGGTCGTTGCCCTGCACCGCCACGTCGGCCTTGAAACGACCGTGCACGGAGTCGTACTTGAGCATGTACGCCAGATAGTCCGGCTCCAGTTGATGGCC

*guaA allel 7*

CGCTGGTACGGCGTGCAGTTCCACCCGGAAGTGACCCACACCCTGCAGGGCCAGGCGCTGCTGCGCCGCTTCGTGGTGGACGTGTGCGGCTGCCAGACCCTGTGGACCGCCGCCAACATCATCGACGACCAGATCGCCCGCGTGCGCGAACAGGTGGGCGATGACGAAGTGATCCTGGGCCTGTCCGGCGGCGTCGATTCGTCCGTGGTGGCCGCGCTGCTGCACAAGGCCATCGGCGAAAAGCTGACCTGCGTGTTCGTGGATACCGGCCTGCTGCGCTGGCAGGAAGGCGACCAGGTGATGGCGATGTTCGCCGAACACATGGGCGTGAAGGTCGTTCGCGTGAATGCCGCCGACCGTTACTTCGCCGCGCTGGAAGGCGTGAGCGACCCGGAAGCCAAGCGCAAGATCATCGGTAACCTGTTCGTTGAGATCTTCGATGAAGAGTCGAACAAGCTGAGCAACGCCAAGTGGCTGGCGCAGGGCACCATCTACCCGGACGTGATCGAGTCGGCTGGCAGCAAGACCGGCAAGGCGCATGTGATCAAGAGCCACCACAACGTGGGCGGCCTGCCAGAGCACATGAAGCTGGGCCTGGTGGAGCCGCTGCGCGAGCTGTTCAAGGACGAAGTGCGCCGCCTGGGTGTTGAACTGGGCCTGCCGCGCACCATGGTCT

*mutM allel 7*

AACGTCCTGCGCTCGGCACCTGCAGGGCCGCCGCGTGCATGGCGTGATCCTGCGCCGCGCCGACCTGCGCTGGCCGATTCCGCCGGAAGTGGCCGAGCTGCTGCCGGGGCAGCGCATCGAGGACATCCGCCGTCGCGCCAAGTACCTGCTGCTGGACACCGCCATCGGCAGCGCCGTGCTGCACCTGGGCATGTCCGGCAGCCTGCGTGTGCTGCCCGGCGATACCCCGCTGCGCGCGCACGACCATGTGGATATCAGCCTGGACAACGGCCGCCTGTTGCGCTTCAACGACCCGCGCCGTTTCGGCAGCCTGCTCTGGCAGCCGGCCGGCGAAGTCCACCCGCTGCTGCAGGGGCTGGGCCCGGAGCCGCTGGACGATGCCTTCGACGGGGACTACCTGTTCTCCCGCAGCCGTGGCCGCAGCGCGCCGGTGAAGACCTTCCTGATGGACCAGGCGGTGGTGGTGGGCGTGGGCAACATCACGCCGACGCCGGTATCGCCCTG

*nuoD allel 80*

CTAACCATGAACTTCGGCCCGCAGCATCCGGCCGCGCACGGTGTGCTGCGCCTGATCCTGGAGATGGACGGCGAAACCATCATGCGCGCCGACCCGCACGTGGGTCTGCTGCACCGTGGTACCGAAAAGCTGGCCGAGTCCAAGCCGTTCAACCAGTCGATCGGCTACATGGATCGCCTGGATTACGTGTCGATGATGTGCAACGAGCACGCCTACGTGCGCGCGATCGAGACCCTGATGGGCATCGAGGCGCCGGAGCGTGCGCAGTACATCCGCACCATGTACGACGAAATCACCCGCATCCTCAACCACCTGATGTGGCTTGGCTCCAATGCGCTCGATCTGGGTGCGATGGCGGTGATGCTGTACGCCTTCCGCGAGCGCGAAGAGCTGATGGACTGCTATGAAGCGGTCTCCGGCGCGCGCATGCACGCGGCGTACTACCGTCCGGGCGGTGTCTACCGCGACCTGCCGGACCACATGCCGAAGTACAAGA

*ppsA allel93*

GTTACATGACCGTGCGCGGCGTAAGAGCTGCGCAACACCTTCTCGATCAGCGACGAGGACGTGCAGGAACTGTCCAAGCAGGCGCTGGTCATCGAAAAGCACTACGGCCGCCCGATGGACATCGAGTGGGCCAAGGACGGTGTCAGCGGTAAGCTGTTCATCGTGCAGGCGCGCCCGGAAACGGTGAAGTCGCGCAGCCACGCCACCCAGATCGAGCGCTTCGCGCTGACCGAAAAGGGCGGCAACGTGCTGGCCGAAGGCCGTGCCGTCGGTGCCAAGATCGGTTCGGGCGTGGCCCGCGTGGTCAAGACGCTGGACGACATGAACCGCGTGCAGCCGGGCGACGTGCTGATCGCCGACATGACCGACCCGGACTGGGAACCGGTGATGAAGCGCGCTTCGGCCATCGTCACCAATCGCGGTGGCCGTACCTGCCACGCCGCGATCATCGCGCGCGAGCTGGGCGTGCCGGCCGTGGTCGGTTCGGGCAACGCCACCAAGGTCATCGAAGATGTCAACAGAGAGCAA

*recA allel 74*

AAGAAGCGCGCCCTCGCTGCAGCTCTGGGCCAGATCGAAAAGCAGTTCGGCAAGGGCTCGGTGATGCGCATGGGCGACCGCGTGGTCGAGCCCGTCGAAGCCATCCCGACCGGTTCGCTGATGCTCGACATCGCACTGGGCATCGGCGGTCTGCCGAAGGGCCGTGTCGTCGAGATCTACGGGCCGGAATCCTCGGGCAAGACCACGCTGACCCTGCAGGCCATCGCCGAATGCCAGAAGATGGGCGGCACCGCGGCCTTCATCGACGCCGAGCACGCGCTGGACCCGATCTACGCCGCCAAGCTGGGCGTGAACGTGGACGACCTGCTGCTGTCGCAGCCGGATACCGGTGAGCAGGCGCTGGAAATCGCCGACATGCTGGTCCGTTCGGGTTCGGTCGACATCCTGGTGATCGACTCGGTCGCCGCGTTGACCCCGAAGGCTGAAATCGAAGGCGAGATGGGCGATCAGCTGCCGGGCCTGCAGGCCCGCCTGATGAGCCAGGCACTGCGCAAGCTGACCGGCAACATCAAGCGCTCCAACACCCTGGTGGTCTTCATCAACCAGCTGCGCATGAAGATCGGTGTGATGATGCCGGGCCAGAGCCCGGAAACCACCACCGGCGGCAACGCGCTGAAGTTCTATGCCTCGGTGCGCCTGGACATCCGCCGTATCGGCGCGATCAAGAAGGGCGACGAGATCATCGGCAACCAGACCAAGATCAAGGTCGTCAAGAACAAGCTGGCGCCTCCGTTCAA

**Strain no. 435**

*atpD allel 6*

TCGGCGCTGGCACCCTGGGCCGCAGGCATCTCGGTGCCGGTCGGCGCCCGCGGCATCTCCGTGCCGGTCGGTGCTGGCACCCTGGGCCGCATCATGGACGTGCTCGGCCGTCCGATCGACGAAGCCGGCCCGGTGGCCGCCAGCGACAGCTGGGAAATCCACCGTGCGGCCCCGTCGTACGAAGACCAGTCCCCGGCCACCGAGCTGCTGGAAACCGGCATCAAGGTCATCGACCTGATGTGCCCGTTCGCCAAGGGCGGCAAGGTCGGCCTGTTCGGCGGCGCCGGCGTCGGCAAGACCGTCAACATGATGGAACTGATCAACAACATCGCCAAGGCGCACAGCGGTCTGTCCGTGTTCGCCGGCGTGGGTGAGCGTACCCGTGAGGGCAACGACTTCTACCACGAAATGAAGGACTCCAACGTCCTCGACAAGGTGGCGATGGTGTACGGCCAGATGAACGAGCCGCCGGGCAACCGTCTGCGCGTCGCCCTGACCGGCCTGACCATGGCCGAGTACTTCCGCGACGAGAAGGACGAAAACGGCAAGGGCAAGGACGTCCTGCTGT

*gapA allel 1*

GCGTACGTCCTGCGCTCGGCGGTGCTGAACTTCGGCGACGACTCGAAATCGTGGCCATCAACGATCTGCTGGAGCCGGACTACCTGGCGTACATGCTCAAGTACGACTCCGTGCACGGCCGCTTCAAGGCCGACGTGGCAGTGCAGGGCAACGACCTGCTGGTCAACGGCAAGAAGATCCGCCTGACCCAGGAACGCGACCCGGCCAACCTGAAGTGGGATGAAGTCGGTGCCGACGTGGTGCTGGAAGCCACCGGCCTGTTCCTGACCAAGGAAACCGCGCAGAAGCACATCGATGCCGGCGCCAAGAAGGTCATCATGTCGGCGCCGTCGAAGGACGACACGCCGATGTTCGTCTACGGCGTGAACGACAAGACCTACGCCGGCCAGGCGATCATTTCCAACGCCTCGTGCACCACCAACTGCCTGGCCCCGCTGGCCAAGGTCATCAATGACAAGTGGGGCATCAAGCGCGGCCTGATGACCACCGTGCATGCGGCAACCGCCACCCAGAAGACCGTCGATGGCCCGTCCAACAAGGACTGGCGCGGTGGCCGTGGCATCCTGGAGAACATCATCCCGTCGTCCACCGGTGCGGCCAAGGCCGTCGGCGTGGTCATCCCGGAACTGAACAAGAAGCTGACCGGCATGAGCTTCCGCGTCCCGACCTCGGACGTGTCGGTGGTCGACCTGACCGTCGAACTGGAAAAGGAAGCCACCTACGCCGAGATCTGCGCGGAAGTGAAGGCACAGAGCGAAGGCCCGCTGAAGGGCATCCTGGGCTACACCGAAGACAAGGTGGTGGCCACCGATTTCCGCGGCGAAACCTGCACTTCGGTGTTCGACGCCGACGCTGGCATCGCCC

*guaA allel 39*

CCACACCCTGCAGGGACCAGGCGCTGCTGCGCCGCTTCGTGGTGGATGTGTGCGGTTGCCAGACCCTGTGGACCGCCGCCAACATCATCGACGACCAGATCGCCCGCGTAAGAGATCAAGTGGGCGATGACGAAGTGATCCTGGGCCTGTCCGGCGGCGTCGATTCGTCCGTCGTGGCTGCGCTGCTGCACAAGGCCATCGGCGAGAAGCTGACCTGCGTGTTCGTGGACACCGGCCTGCTGCGCTGGCAGGAAGGCGACCAGGTGATGGCGATGTTTGCCGAACACATGGGCGTGAAGGTCGTGCGCGTAAATGCCGCCGACCGCTACTTCAGCGCGCTGGAAGGCGTGAGCGACCCGGAAGCCAAGCGCAAGATCATCGGCAACCTGTTCGTTGAGATCTTCGACGAAGAGTCGAACAAGCTGAAGAACGCCAAGTGGCTGGCGCAGGGCACCATCTACCCGGACGTGATCGAGTCGGCCGGCAGCAAGACCGGCAAGGCGCATGTGATCAAGAGCCACCACAACGTGGGCGGCCTGCCGGAACACATGAAGCTGGGCCTGGTGGAGCCGCTGCGCGAGCTGTTCAAGGACGAAGTGCGCCGCCTCGGCGTTGAGCTGGGCCTGCCGCGCACCATGGTCTC

*mutM allel 19*

AACTGCCACGCCGTCGCGTGCATGGCGTGATCCTGCGCCGCGCCGATCTGCGCTGGCCGATCCCGCCGGAAGTGGCCGAGCTGCTGCCGGGACAGCGCATCGAGGAGATCCGCCGGCGCGCCAAGTACCTGTTGCTGGATACCGCCATCGGCAGCGCGGTGCTGCACCTGGGCATGTCCGGCAGCCTGCGTGTGCTGCCCGGTGATACGCCGGTGCGCGCCCACGACCACGTCGACATCAGCCTGGACAACGGCCGCCTGCTGCGCTTCAACGACCCGCGCCGCTTCGGCAGCTTGCTCTGGCAGCCGGCCGGCGAGATCCACCCGCTGTTGCAGGGGCTGGGCCCGGAGCCACTGGACGACGCGTTCGACGGCGACTACCTGTTCGCCCGCAGCCGTGGCCGCAGCGCGCCGGTGAAGACGTTCCTGATGGACCAGGCGGTGGTGGTGGGGGTGGGCAATC

*nuoD allel 95*

AACCATGAACTTCGGCCCGCAGCATCCGGCCGCGCACGGTGTGCTGCGCCTGATCCTGGAAATGGACGGCGAAACCATCATGCGCGCCGACCCGCACGTGGGTCTGCTGCACCGTGGTACCGAAAAGCTGGCCGAGTCCAAGCCGTTCAACCAGTCGATCGGCTACATGGATCGCCTGGACTACGTGTCGATGATGTGCAACGAGCACGCCTACGTGCGCGCGATCGAGACCCTGATGGGCATCGAGGCGCCGGAGCGTGCGCAGTACATCCGCACCATGTACGACGAAATCACCCGCATCCTCAACCACCTGATGTGGCTGGGTTCCAACGCGCTCGACCTGGGTGCGATGGCGGTGATGCTGTACGCCTTCCGCGAGCGCGAAGAGCTGATGGACTGCTATGAAGCGGTCTCCGGCGCGCGCATGCACGCGGCGTACTACCGTCCGGGCGGTGTCTACCGCGACCTGCCGGACCACATGCCGAAGTACAAGA

*ppsA allel 33*

AGACAGAACTGCGCACCACCTTCTCGATCAGCGACGAGGACGTGCAGGAACTGTCCAAGCAGGCGCTGGTCATCGAAAAGCACTACGGCCGCCCGATGGATATCGAGTGGGCCAAGGACGGTGTCAGCGGCAAGCTGTTCATCGTGCAGGCGCGCCCGGAGACGGTGAAGTCGCGCAGCCATGCCACCCAGATCGAGCGCTTCGCGCTGACCGAAAAGGGCGGCAACGTGCTGGCCGAGGGCCGTGCCGTTGGTGCCAAGATCGGCTCGGGCGTGGCCCGCGTGGTGAAGACGCTGGACGACATGAACCGCGTGCAGCCGGGCGACGTGCTGATCGCCGACATGACCGACCCGGACTGGGAACCGGTGATGAAGCGTGCCTCGGCCATCGTCACCAATCGCGGTGGCCGTACCTGTCATGCCGCGATCATCGCGCGCGAGCTGGGCGTGCCGGCCGTGGTCGGTTCGGGCAACGCCACCAAGGTCATCGAAGGGCACCCGAAAGAGTGCAGAACTCGG

*recA allel 78*

GCGCGCCCTCGCTGCAGCTCTGGGCCAGATCGAAAAGCAGTTCGGCAAGGGCTCGGTGATGCGCATGGGCGACCGCGTGGTCGAGCCCGTCGAAGCCATCCCGACCGGTTCGCTGATGCTCGACATCGCGCTGGGCATTGGCGGTCTGCCGAAGGGCCGTGTCGTTGAAATCTACGGGCCGGAATCCTCGGGCAAGACCACGCTGACCCTGCAGGCCATCGCTGAATGCCAGAAGATGGGCGGCACCGCGGCCTTCATCGACGCCGAGCACGCGCTGGACCCGATCTACGCTGCCAAGCTGGGCGTGAACGTGGACGACCTGCTGCTGTCGCAGCCTGATACCGGCGAGCAGGCGCTGGAAATCGCCGACATGCTCGTCCGTTCGGGTTCGGTGGACATCCTGGTGATCGACTCGGTCGCCGCGCTGACCCCGAAGGCCGAAATCGAAGGCGAGATGGGCGACCAGCTGCCGGGCCTGCAGGCCCGCCTGATGAGCCAGGCGCTGCGCAAGCTGACCGGCAACATCAAGCGCTCCAACACCCTGGTGGTCTTCATCAACCAGCTGCGCATGAAGATCGGCGTGATGATGCCGGGCCAGAGCCCGGAAACCACCACCGGCGGCAACGCGCTGAAGTTCTACGCTTCGGTGCGCCTGGACATCCGCCGTATCGGCGCGATCAAGAAGGGCGACGAGATCATTGGCAACCAGACCAAGATCAAGGTCGTCAAGAACAAGCTGGCGCCTCCGTT

**Strain no. 6840 ST4**

*atpD allel 1*

AGKTTTSTKCACTCTACGCTACACCCTGGCCGGTACCGAAGTGTCGGCACTGCTGGGCCGTACGTGGCATCTCGGTGCCGGTCGGCGCCGGCACCCTGGGCCGCATCATGGACGTGCTCGGCCGTCCGATCGACGAAGCCGGCCCGGTGGCCGCCAGCGACAGCTGGGAAATCCACCGTGCGGCCCCGTCGTACGAAGACCAGTCCCCGGCCACCGAACTGCTGGAAACCGGCATCAAGGTCATCGACCTGATGTGCCCGTTCGCCAAGGGCGGCAAGGTCGGCCTGTTCGGCGGCGCCGGCGTCGGCAAGACCGTCAACATGATGGAGCTGATCAACAACATCGCCAAGGCGCACAGCGGTCTGTCCGTGTTCGCCGGCGTGGGTGAGCGTACCCGTGAGGGCAACGACTTCTACCACGAAATGAAGGACTCCAACGTCCTCGACAAGGTGGCGATGGTGTACGGCCAGATGAACGAGCCGCCGGGCAACCGTCTGCGCGTCGCCCTGACCGGCCTGACCATGGCCGAGTACTTCCGCGACGAGAAGGACGAAAACGGCAAGGGCAAGGACGTCCTGCTGTTCGTCGACAATGCCGTC

*gapA allel 4*

TCCTGCGCTCGGCGGTGCTGAACTTCGGCGACGACTCGAAATCGTGGCCATCAACGATCTGCTGGAGCCGGACTACCTGGCGTACATGCTCAAGTACGACTCCGTGCACGGCCGCTTCAAGGCCGACGTGGCGGTGCAGGGCAACGACCTGCTGGTCAACGGCAAGAAGATCCGCCTGACCCAGGAACGCGACCCGGCCAACCTGAAGTGGGATGAAGTCGGTGCCGACGTGGTGCTGGAAGCCACCGGCCTGTTCCTGACCAAGGAAACCGCGCAGAAGCACATCGATGCCGGCGCCAAGAAGGTCATCATGTCGGCGCCGTCGAAGGACGACACGCCGATGTTCGTCTACGGCGTGAACGACAAGACCTACGCCGGCCAGGCGATCATTTCCAACGCCTCGTGCACCACCAACTGCCTGGCCCCGCTGGCCAAGGTCATCAATGACAAGTGGGGCATCAAGCGCGGCCTGATGACCACCGTGCATGCGGCAACCGCCACCCAGAAGACCGTCGATGGCCCGTCCAACAAGGACTGGCGCGGTGGCCGTGGCATCCTGGAGAACATCATCCCGTCGTCCACCGGTGCGGCCAAGGCCGTCGGCGTGGTCATCCCGGAACTGAACAAGAAGCTGACCGGCATGAGCTTCCGCGTCCCGACCTCGGACGTGTCGGTGGTCGACCTGACCGTCGAACTGGAAAAGGAAGCCACCTACGCCGAGATCTGCGCGGAAGTGAAGGCACAGAGCGAAGGCCCGCTGAAGGGCATCCTGGGCTACACCGAAGACAAGGTGGTGGCCACCGATTTCCGCGGCGAAACCTGCACTTCGGTGTTCGACGCCGACGCTGGTATCG

*guaA allel 7*

CCGGATTCCGGAGTGACCACACCCTGCAGGGCCAGGCGCTGCTGCGCCGCTTCGTGGTGGACGTGTGCGGCTGCCAGACCCTGTGGACCGCCGCCAACATCATCGACGACCAGATCGCCCGCGTGCGCGAACAGGTGGGCGATGACGAAGTGATCCTGGGCCTGTCCGGCGGCGTCGATTCGTCCGTGGTGGCCGCGCTGCTGCACAAGGCCATCGGCGAAAAGCTGACCTGCGTGTTCGTGGATACCGGCCTGCTGCGCTGGCAGGAAGGCGACCAGGTGATGGCGATGTTCGCCGAACACATGGGCGTGAAGGTCGTTCGCGTGAATGCCGCCGACCGTTACTTCGCCGCGCTGGAAGGCGTGAGCGACCCGGAAGCCAAGCGCAAGATCATCGGTAACCTGTTCGTTGAGATCTTCGATGAAGAGTCGAACAAGCTGAGCAACGCCAAGTGGCTGGCGCAGGGCACCATCTACCCGGACGTGATCGAGTCGGCTGGCAGCAAGACCGGCAAGGCGCATGTGATCAAGAGCCACCACAACGTGGGCGGCCTGCCAGAGCACATGAAGCTGGGCCTGGTGGAGCCGCTGCGCGAGCTGTTCAAGGACGAAGTGCGCCGCCTGGGTGTTGAACTGGGCCTGCCGCGCACCATGGTCTCCGGCCCATTCGAAA

*mutM allel 7*

TAACGTCCTGCGCTCGGCACCTGCAGGGCCGCCGCGTGCATGGCGTGATCCTGCGCCGCGCCGACCTGCGCTGGCCGATTCCGCCGGAAGTGGCCGAGCTGCTGCCGGGGCAGCGCATCGAGGACATCCGCCGTCGCGCCAAGTACCTGCTGCTGGACACCGCCATCGGCAGCGCCGTGCTGCACCTGGGCATGTCCGGCAGCCTGCGTGTGCTGCCCGGCGATACCCCGCTGCGCGCGCACGACCATGTGGATATCAGCCTGGACAACGGCCGCCTGTTGCGCTTCAACGACCCGCGCCGTTTCGGCAGCCTGCTCTGGCAGCCGGCCGGCGAAGTCCACCCGCTGCTGCAGGGGCTGGGCCCGGAGCCGCTGGACGATGCCTTCGACGGGGACTACCTGTTCTCCCGCAGCCGTGGCCGCAGCGCGCCGGTGAAGACCTTCCTGATGGACCAGGCGGTGGTGGTGGGCGTGGGCAACATCACGCCGACGCCGGTATCGCCCTG

*nuoD allel 28*

TAACCATGAACTTCGGCCCGCAGCATCCGGCCGCTCACGGTGTGCTGCGCCTGATCCTGGAAATGGACGGTGAAACCATCATGCGCGCCGACCCGCACGTGGGTCTGCTGCACCGTGGTACCGAAAAGCTGGCCGAGTCCAAGCCGTTCAACCAGTCGATCGGCTACATGGATCGCCTGGATTACGTGTCGATGATGTGCAACGAGCACGCCTACGTGCGCGCGATCGAGACCCTGATGGGCATCGAGGCGCCGGAGCGTGCGCAGTACATCCGCACCATGTACGACGAGATCACCCGCATCCTCAACCACCTGATGTGGCTGGGCTCCAACGCGCTCGACCTGGGTGCGATGGCGGTGATGCTGTACGCCTTCCGCGAGCGCGAAGAGCTGATGGACTGCTATGAAGCAGTCTCTGGCGCACGCATGCACGCGGCGTACTACCGTCCGGGCGGTGTCTACCGCGACCTGCCGGACCACATGCCGAAGTACAAGA

*ppsA allel 19*

AAGGTCGAACTGCGCAACACCTTCTCGATCAGCGACGAGGACGTGCAGGAACTGTCCAAGCAGGCGCTGGTCATCGAAAAGCACTACGGCCGCCCGATGGACATCGAGTGGGCCAAGGACGGTGTCAGCGGCAAGCTGTTCATCGTGCAGGCACGCCCGGAAACGGTGAAGTCGCGCAGCCACGCCACCCAGATCGAGCGCTTCGCGCTGACCGAAAAGGGCGGCAACGTGCTGGCCGAAGGTCGTGCCGTCGGCGCCAAGATCGGTTCGGGCGTGGCCCGCGTGGTCAAGACGCTGGACGACATGAACCGCGTGCAGCCGGGCGACGTGCTGATCGCCGACATGACCGACCCGGACTGGGAACCGGTGATGAAGCGCGCCTCGGCCATCGTCACCAATCGTGGTGGCCGCACCTGCCACGCCGCGATCATCGCGCGCGAGCTGGGCGTGCCCGCCGTGGTCGGTTCGGGCAATGCCACCAAGGTCATTGAAGATGGCTGGCC

*recA allel 6*

GGGGGGACGCGTCTCTGGGGACAGATCGAGAAGCAGTTCGGCAAGGGCTCGGTGATGCGCATGGGCGACCGCGTGGTCGAGCCCGTCGAAGCCATCCCGACCGGTTCGCTGATGCTCGACATCGCACTGGGCATTGGCGGTCTGCCGAAGAACCCGTCGAAGCCATCCCGACCGGTTCGCTGATGCTCGACATCGCGCTGGGCATTGGTGGTCTGCCGAAGGGCCGTGTCGTCGAGATCTACGGGCCGGAATCCTCGGGCAAGACCACCTTGACCCTGCAGGCCATCGCCGAATGCCAGAAGATGGGCGGCACCGCGGCCTTCATCGACGCCGAGCACGCGCTGGACCCGATCTACGCCGCCAAGCTGGGCGTGAACGTGGACGACCTGCTGCTGTCGCAGCCGGACACCGGTGAGCAGGCGCTGGAAATCGCCGACATGCTGGTCCGTTCGGGTTCGGTCGACATCCTGGTGATCGACTCGGTTGCCGCGCTGACCCCGAAGGCCGAAATCGAAGGCGAGATGGGCGACCAGCTGCCAGGCCTTCAGGCCCGCCTGATGAGCCAGGCGCTGCGCAAGCTGACCGGCAACATCAAGCGCTCCAACACCCTGGTGATCTTCATCAACCAGCTGCGCATGAAGATCGGCGTGATGATGCCGGGCCAGAGCCCGGAAACCACCACGGGTGGCAACGCGGTCCGCCTGGACATCCGCCGTATCGGCGCGATCAAGAAGGGTGACGAGATCATCGGCAACCAGACCAAGATCAAGGTGGTCAAGAACAAG

**Strain no. 11865 ST15**

*atpD allel 10*

GAGKGCGGCATTTCGGTGCCGGTCGGCGCCGGCACCCTGGGCCGCATCATGGACGTGCTGGGCCGTCCGATCGACGAAGCCGGCCCGGTCGCTGCCAGCGACAACTGGGAAATCCACCGCGCTGCGCCGTCGTATGAAGACCAGTCCCCGGCCACCGAGCTGCTGGAAACCGGCATCAAGGTCATCGACCTGATGTGCCCGTTCGCCAAGGGCGGCAAGGTCGGCCTGTTCGGCGGCGCCGGCGTCGGCAAGACCGTCAACATGATGGAGCTGATCAACAACATCGCCAAGGCGCACAGCGGCCTGTCCGTGTTCGCCGGCGTGGGTGAGCGTACCCGTGAGGGCAACGACTTCTACCACGAAATGAAGGACTCCAACGTCCTGGACAAGGTGGCGATGGTGTACGGCCAGATGAACGAGCCGCCGGGCAACCGCCTGCGCGTCGCGCTGACCGGCCTGACCATGGCCGAGTACTTCCGCGACGAGAAGGACGAAAACGGCAAGGGCAAGGACGTCCTGTTGTTCGTCGACAA

*gapA allel 29*

TAACGTCCTGCGCTCGGCGGTGCTGAACTTCGGCGACGACTCGAAATCGTGGCCATCAACGATCTGCTGGAGCCGGATTACCTGGCCTACATGCTCAAGTACGACTCCGTGCACGGCCGCTTCGAGGCCGACGTGGCGGTGCAGGGCAACGACCTGCTGGTCAACGGCAAGAAGATCCGCCTGACCCAGGAGCGCGACCCGGCCAACCTGAAGTGGGACGAAGTCGGCGCCGACGTGGTGCTGGAAGCCACCGGCCTGTTCCTGACCAAGGAAACCGCGCAGAAGCACATCGATGCCGGCGCCAGGAAGGTCATCATGTCGGCCCCGTCGAAGGACGACACGCCGATGTTCGTGTTCGGCGTGAACGACAAGACCTATGCTGGCCAGGCAATCATCTCCAACGCCTCGTGCACCACCAACTGCCTGGCCCCGCTGGCCAAGGTCATCAACGACAAGTGGGGCATCAAGCGCGGCCTGATGACCACCGTGCATGCGGCCACCGCCACCCAGAAGACCGTCGATGGCCCGTCCAACAAGGACTGGCGCGGTGGCCGTGGCATCCTGGAAAACATCATTCCCTCGTCCACCGGTGCGGCCAAGGCCGTCGGCGTGGTCATCCCGGAACTGAACAAGAAGCTGACCGGCATGAGCTTCCGCGTCCCGACCTCGGACGTGTCGGTGGTCGACCTGACCGTCGAGCTGGAGAAGGAAGCCACCTACGCCGAGATCTGCGCTGAAGTGAAGGCACAGAGCGAAGGCGCGCTGAAGGCGCGCTGAAGGGCATCCTGGGCTACACCGAAGACAAGGTGGTGGCCACCGATTTYCGCGGCGAGACCTGCACCTCGGTGTTYGACGCCGACGCCGGTATCGCCCTG

*guaA allel 21*

GCCGGATTGCGCCGCTTCGTGGTGGATGTGTGCGGCTGCCAGACCCTGTGGACCGCCGCCAACATCATCGACGACCAGATCGCCCGCGTGCGCGAGCAGGTGGGCGATGACGAAGTGATCCTGGGCCTGTCCGGCGGCGTCGATTCGTCCGTGGTGGCTGCGCTGCTGCACAAGGCCATCGGCGAAAAGCTGACCTGCGTGTTCGTGGATACCGGCCTGCTGCGCTGGCAGGAAGGCGACCAGGTGATGGCGATGTTCGCCGAGCACATGGGCGTGAAGGTGGTGCGCGTGAATGCCGCCGACCGCTACTTCGCCGCGCTGGAAGGCGTGAGCGACCCAGAAGCCAAGCGCAAGATCATCGGCAACCTGTTCGTTGAGATCTTCGACGAAGAGTCGAACAAGCTGAAGAACGCCAAGTGGCTGGCGCAGGGCACCATCTACCCGGACGTGATCGAGTCGGCCGGCAGCAAGACTGGCAAGGCGCATGTGATCAAGAGCCACCACAACGTGGGCGGCCTGCCGGAGCACATGAAGCTGGGCCTGGTGGAGCCGCAAA

*mutM allel 21*

AACGCAGGGCCGGCGCGTGCATGGCGTGATCCTGCGCCGCGCCGACCTGCGCTGGCCGATTCCACCGGAAGTGGCCGAGCTGCTGCCGGGGCAGCGCATCGAGGACATCCGCCGGCGCGCCAAGTACCTGCTGCTGGATACCGCCATCGGCAGCGCCGTGCTGCACCTGGGCATGTCCGGCAGCCTGCGCGTGCTGCCCGGTGATACCCCGCTGCGGGCGCACGACCACGTCGACATCAGCCTGGACAACGGCCGCCTGCTGCGCTTCAACGACCCGCGCCGCTTCGGCAGCCTGCTCTGGCAGCCGGCTGGGGAGATCCACCCGCTGCTGCAGGGGCTGGGCCCGGAGCCACTGGACGACGCGTTCGACGGCGACTACCTGTTCGACCGCAGCCGGGGCCGCAGCGCGCCGGTGAAGACCTTCCTGATGGACCAGGCGGTGGTGGTGGGCGTGGGCAACATC

*nuoD allel 32*

AACCATGAACTTCGGCCCGCAGCATCCGGCCGCTCACGGTGTGCTGCGCCTGATCCTGGAAATGGACGGCGAAACCATCATGCGCGCCGACCCGCACGTGGGTCTGCTGCACCGTGGTACCGAAAAGCTGGCCGAGTCCAAGCCGTTCAACCAGTCGATCGGCTACATGGATCGACTGGACTACGTGTCGATGATGTGCAACGAGCACGCCTACGTGCGCGCGATCGAGACCCTGATGGGCATCGAGGCGCCGGAGCGTGCCCAGTACATCCGCACCATGTACGATGAAATCACCCGCATCCTCAACCACCTGATGTGGCTGGGCTCCAACGCGCTCGACCTGGGCGCGATGGCGGTGATGTTGTACGCCTTCCGCGAACGCGAAGAGCTGATGGACTGCTACGAAGCGGTTTCCGGCGCGCGCATGCACGCGGCGTACTACCGTCCGGGCGGTGTCTACCGCGACCTGCCGGACCACATGCCGAAGTACAAGA

*ppsA allel 32*

GACAGAACTGCGCACCACCCGAACTGCGCACCACCTTCTCGATCAGCGACGAGGACGTGCAGGAACTGGCCAAGCAGGCGCTGGTCATCGAAAAGCACTACGGCCGCCCGATGGACATCGAGTGGGCCAAGGACGGTGTCAGCGGCAAGCTGTTCATCGTGCAGGCGCGCCCGGAAACGGTGAAGTCGCGCAGCCACGCCACCCAGATCGAGCGCTTCGCGCTGACCGAGAAGGGCGGCAAGGTGCTGGCCGAGGGCCGCGCCGTCGGCGCCAAGATCGGTTCGGGCGTGGCCCGCGTGGTGAAGACGCTGGACGACATGAACCGCGTGCAGCCGGGCGACGTGCTGATCGCCGACATGACCGATCCCGATTGGGAACCGGTGATGAAGCGCGCCTCGGCCATCGTCACCAACCGCGGTGGCCGCACCTGCCACGCCGCGATCATCGCGCGCGAGCTGGGCGTGCCGGCCGTGGTCGGTTCGGGCAACGCGACCCAGGTCATCGAGCGAAAGAGTGCAGA

*recA allel 10*

AAGAAGCGCGCCTTGGCTGCTGCTCTGGGCCAGATCGAGAAGCAGTTCGGCAAGGGCTCGGTGATGCGCATGGGCGACCGCGTGGTCGAGCCCGTCGAAGCCATCCCGACCGGTTCGCTGATGCTCGACATCGCACTGGGCATTGGCGGTCTGCCGAAGGGCCGTGTCGTTGAGATCTACGGCCCGGAATCCTCGGGCAAGACTACCCTGACCCTGCAGGCCATCGCCGAATGCCAGAAGATGGGCGGTACCGCGGCCTTCATCGACGCCGAGCACGCGCTTGACCCGATCTACGCCGCGAAGCTGGGCGTGAACGTGGACGACCTGCTGCTGTCGCAGCCGGACACCGGTGAGCAGGCGCTGGAAATCGCCGACATGCTGGTCCGTTCGGGCTCGGTTGACATCCTGGTGGTCGACTCGGTCGCCGCGCTGACCCCGAAGGCCGAAATCGAAGGCGAAATGGGTGACCAGCTGCCGGGCCTGCAGGCCCGCCTGATGAGCCAGGCGCTGCGCAAGCTGACCGGCAACATCAAGCGTTCCAACACCCTGGTGATCTTCATCAACCAGCTGCGCATGAAGATCGGCGTGATGATGCCGGGCCAGAGCCCGGAAACCACCACCGGTGGCAACGCGCTGAAGTTCTATGCCTCGGTCCGCCTGGACATCCGCCGTATCGGTGCGATCAAGAAGGGCGATGAGATCATCGGCAACCAGACCAAGATCAAGGTCGTCAAGAACAAGCTGGCGCCTCCGTTCAAGCA
